# The medium-size noncoding RNA transcriptome of *Ostreococcus tauri*, the smallest living eukaryote, reveals a large family of small nucleolar RNAs displaying multiple genomic expression strategies

**DOI:** 10.1093/nargab/lqaa080

**Published:** 2020-10-09

**Authors:** Laurie Bousquet, Claire Hemon, Paul Malburet, François Bucchini, Klaas Vandepoele, Nigel Grimsley, Hervé Moreau, Manuel Echeverria

**Affiliations:** Sorbonne Université, CNRS, Laboratoire de Biologie Intégrative des Organismes Marins , UMR7232, F-66650 Banyuls sur Mer, France; Sorbonne Université, CNRS, Laboratoire de Biologie Intégrative des Organismes Marins , UMR7232, F-66650 Banyuls sur Mer, France; Sorbonne Université, CNRS, Laboratoire de Biologie Intégrative des Organismes Marins , UMR7232, F-66650 Banyuls sur Mer, France; Department of Plant Systems Biology,VIB, 9052 Ghent, Belgium; Department of Plant Biotechnology and Bioinformatics, Ghent University, 9052 Ghent, Belgium; Department of Plant Systems Biology,VIB, 9052 Ghent, Belgium; Department of Plant Biotechnology and Bioinformatics, Ghent University, 9052 Ghent, Belgium; Bioinformatic Institute Ghent, Ghent University, 9052 Ghent, Belgium; Sorbonne Université, CNRS, Laboratoire de Biologie Intégrative des Organismes Marins , UMR7232, F-66650 Banyuls sur Mer, France; Sorbonne Université, CNRS, Laboratoire de Biologie Intégrative des Organismes Marins , UMR7232, F-66650 Banyuls sur Mer, France; Sorbonne Université, CNRS, Laboratoire de Biologie Intégrative des Organismes Marins , UMR7232, F-66650 Banyuls sur Mer, France; Département de Biologie, Université de Perpignan via Domitia, 66860 Perpignan Cedex, France

## Abstract

The small nucleolar RNAs (snoRNAs), essential for ribosome biogenesis, constitute a major family of medium-size noncoding RNAs (mncRNAs) in all eukaryotes. We present here, for the first time in a marine unicellular alga, the characterization of the snoRNAs family in *Ostreococcus tauri*, the smallest photosynthetic eukaryote. Using a transcriptomic approach, we identified 131 *O. tauri* snoRNAs (Ot–snoRNA) distributed in three classes: the C/D snoRNAs, the H/ACA snoRNAs and the MRP RNA. Their genomic organization revealed a unique combination of both the intronic organization of animals and the polycistronic organization of plants. Remarkably, clustered genes produced Ot–snoRNAs with unusual structures never previously described in plants. Their abundances, based on quantification of reads and northern blots, showed extreme differences in Ot–snoRNA accumulation, mainly determined by their differential stability. Most of these Ot–snoRNAs were predicted to target rRNAs or snRNAs. Seventeen others were orphan Ot–snoRNAs that would not target rRNA. These were specific to *O. tauri* or Mamiellophyceae and could have functions unrelated to ribosome biogenesis. Overall, these data reveal an ‘evolutionary response’ adapted to the extreme compactness of the *O. tauri* genome that accommodates the essential Ot–snoRNAs, developing multiple strategies to optimize their coordinated expression with a minimal cost on regulatory circuits.

## INTRODUCTION

Small nucleolar RNAs (snoRNAs), found in the nucleolus, represent a major family of medium-size noncoding RNAs (mncRNAs, 50–300 nt) in all eukaryotes, playing essential roles in ribosome biogenesis (1). Most snoRNAs are either C/D box snoRNAs, that guide *2′−O−ribose* methylation, or H/ACA box snoRNAs that guide pseudouridylation of a specific residue on an RNA target. C/D box snoRNAs have conserved terminal C (RUGAUGA) and D (CUGA) boxes flanked by short inverted repeats (IR) at their 5′ and 3′ends, pairing to form a terminal stem of usually 2–5 bp (2,3). Internal C’ and D’ boxes with less conserved C and D box consensus sequences can be found toward the middle of the snoRNA. Adjacent to the terminal D box and/or to the internal D’ box, an AntiSense Element (ASE) guides the modification of the RNA target residue. H/ACA box snoRNAs are characterized by an ACA box positioned 3 nt upstream from the 3′end of the RNA, and an H (hinge) box (ANANNA) separating two stem structures. These stems contain a bulge with a bipartite ASE that can pair with their cognate RNA target nucleotides. The C/D box and H/ACA box snoRNAs assemble with 4 different nucleolar proteins forming two distinct C/D and H/ACA RiboNucleoProteins (snoRNPs) including fibrillarin, the RNA methylase and dyskerin/Nap57/Cbf5p, the pseudouridine synthase, respectively (2).

Most snoRNAs target ribosomal RNAs (rRNAs), thereby modifying specific residues, for example, 90 residues in yeast and 210 residues in human rRNAs. These modifications are necessary for the correct folding and stability of rRNA scaffolds in functional ribosomes (1,4). SnoRNAs also have additional functions besides post-transcriptional modification. A few highly conserved and essential snoRNAs, like U3, U14 or the MRP RNA, direct cleavage of the 35S/45S pre-rRNA precursors (1,5). Recently, snoRNAs targeting specific acetylation of 18S rRNA residues in yeast, have also been identified (6). Unrelated to ribosome biogenesis, the scaRNAs, located in nuclear Cajal bodies, are structurally similar to snoRNAs but direct modification of small nuclear RNAs (snRNAs) implicated in splicing (7). SnoRNAs also target methylation of mRNA and control alternative splicing, for example of mRNA encoding the serotonin receptor in human brain (8). Very recently a snoRNA targeting tRNA^Met^ methylation has been found that controls its cleavage in response to stress (9), and furthermore snoRNA can be processed into smaller fragments, some of which could have a microRNA (miRNA)-like function (10,11). Finally, ‘orphan’ snoRNAs, for which no target has been identified and could have functions unrelated to ribosome biogenesis, are usually found in different species (12,13).

Remarkably, snoRNAs exhibit very diverse modes of expression, according to their eukaryotic origin (14). In yeast, most snoRNAs are encoded by monocistronic genes driven from their own promoter. In animals most snoRNAs are nested in the introns of protein coding genes and are produced by debranching and processing of the spliced intron. In plants most snoRNAs are encoded by gene clusters, either independently driven from a common Pol II promoter, or as intronic snoRNAs nested in a host gene. In both cases the polycistronic precursors are subsequently processed into mature snoRNAs units (14).

In unicellular algae, the identification and characterization of the snoRNA family has been restricted to the freshwater *Chlamydomonas reinhardtii* (15) and the evolutionary distant *Euglena gracilis* (16). In both species snoRNAs genes were found to be mainly in organized in polycistronic gene clusters, usually producing multiple snoRNA isoforms, much like in higher plants. In unicellular marine algae, 11 C/D box snoRNAs were identified by RNA-seq analysis in the nucleomorph of a Cryptophyte alga (17) and 20 snoRNAs were predicted in *Ostreococcus lucimarinus* by phylogenomic tracing of the origin of eukaryotic snoRNAs (18).

We present here the characterization of the snoRNA family in *Ostreococcus tauri*, a green unicellular marine alga, in the class Mamiellophyceae, an early branch of the green lineage and an important component of marine phytoplankton. *Ostreococcus tauri* is the smallest living eukaryote, with ∼1 μm diameter cells containing a single chloroplast and a single mitochondrion. Its haploid, compact, completely sequenced and annotated nuclear genome of ∼13 Mb has 20 chromosomes (19,20). *Ostreococcus tauri* lacks homologs of Dicer and AGO proteins and consequently, differs from the majority of eukaryotes, including diatoms and *C. reinhardtii*, where RNAi-directed silencing has been shown (21). In *O. tauri*, only highly conserved ncRNAs, namely the rRNAs, the tRNAs, the snRNAs and a single snoRNA, U3, have so far been predicted and their genes annotated in the genome (22). To identify the snoRNA family we designed a transcriptomic approach to clone and sequence the mncRNAs expressed in *O. tauri* cells. In parallel we produced the corresponding small RNA transcriptomes, to clearly delimit the 5′ and 3′ extremities of our snoRNA candidates and evaluate their relative stabilities. Applying stringent criteria for selection of candidates we report here the identification of 131 *O. tauri* snoRNAs (Ot–snoRNAs) distributed in three classes: the C/D box snoRNAs, the H/ACA box snoRNA and the MRP RNA. We describe their very diverse modes of genomic organization, present their conservation across species and compare their abundances, corroborating their transcriptomic quantification by northern blot analyses of representative candidates. Overall these results present the first and most comprehensive view of a snoRNA family in a marine alga, revealing unique features that distinguish it from animal, plant, yeast and *Chlamydomonas* model systems.

## MATERIALS AND METHODS

### Culture conditions and RNA extractions

**Figure 1.**
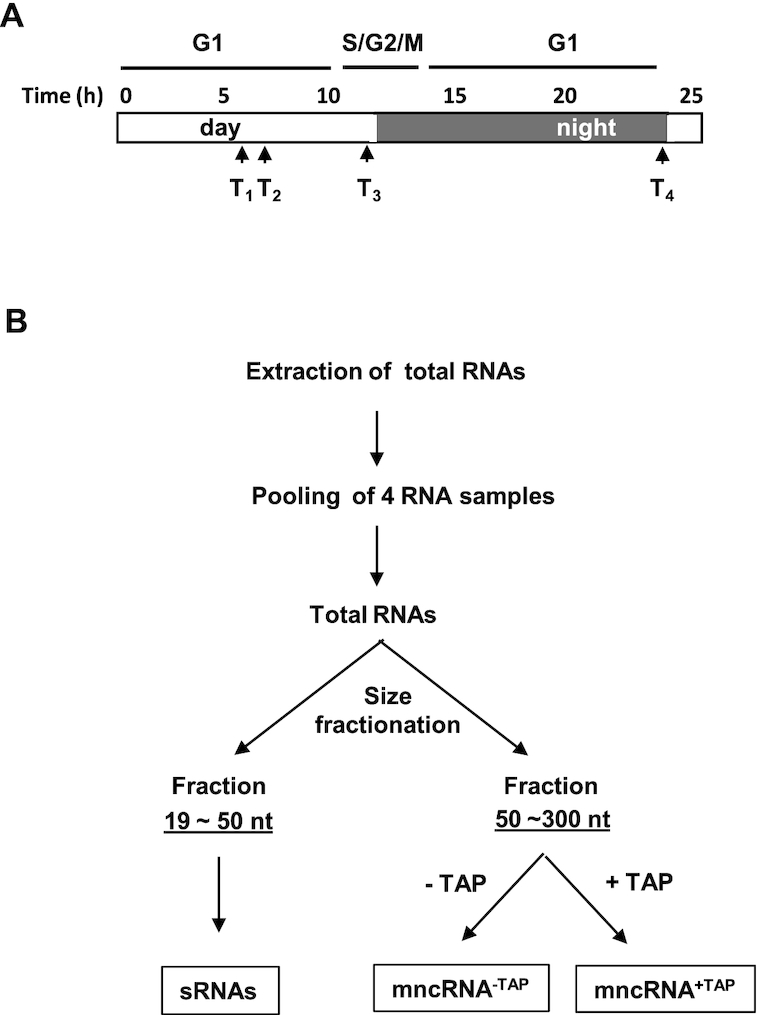
Experimental design to produce the targeted sRNA and mncRNA libraries. (**A**) Clonal cell cultures were grown under a 12 h light/12 h dark regime. T1 to T4 indicate the 4 sampling times taken over a day. Time (h) indicates hours since the beginning of the light period. G1 and G2/M indicate the approximate length of the cell-cycle phases. (**B**) Protocol for production of mncRNA and sRNA libraries (see ‘Materials and Methods’ section). +TAP and −TAP indicate treated or not treated with Tobacco Acid Phosphatase.

Cell cultures of *O. tauri* RCC4221 strain were grown in L1 medium under a 12/12 h light and night cycle inducing a partial synchronization of the cell cycle, as previously described (23). Total RNA fractions were produced from 100 ml of three independent exponentially growing cell cultures. RNA fractions were extracted from cells harvested at four different sampling times (Figure 1A) using the Direct-zol RNA kit (Zymo Research), according to the manufacturer's protocol.

### Production of sRNAs, mncRNA^+TAP^ and mncRNA^−TAP^ transcriptomes

Construction of the three libraries was done commercially by Fasteris (https://www.fasteris.com). Essentially, this included isolation of the small RNA (sRNA, 19–50 nt) and the mncRNAs (50–300 nt) fractions by size fractionation on denaturing polyacrylamide gel electrophoresis and subsequent treatment of half of the mncRNA fraction with Tobacco Acid Pyrophosphatase (TAP). Unfragmented RNA libraries (sRNA, mncRNA^+TAP^ and mncRNA^−TAP^) were sequenced on the Illumina Hi-Seq 2500 platform using a small RNA High-Output protocol and by multiplexing samples. Small RNA libraries (19–50 nt) were produced from single-end reads of 50 bp and middle size RNA libraries of 50–300 nt (mncRNA^+TAP^ and mncRNA^−TAP^) were generated by paired-end reads of 2 × 125 bp. The three libraries (sRNA, mncRNA^+TAP^ and mncRNA^−TAP^) were done in biological duplicates.

The two biological replicates of the sRNA libraries were sequenced to read depths of 21 and 19 million, respectively. The mncRNA^+TAP^ and the mncRNA^−TAP^ libraries replicates were sequenced to reads depths varying between 35 and 45 million.

### Mapping, screening and selection of mncRNA candidates

The quality of reads was checked using FastQC and contaminating adapters were removed using FASTQ−MCF (24). Multimapped reads were dealt with STAR program (25). All parameters used for these programs are given in Supplementary File 1. Reads were mapped on the *O. tauri* nuclear genome of the RCC4221 strain (NC_014426.2–NC_014445.2), the chloroplast (NC_008289.1) and the mitochondrial (NC_008290.1) genomes. Annotations of the nuclear genome were retrieved from the genome Version 2 available in ORCAE database (22). Screening for candidates was done manually, by visualizing the read profiles along the nuclear chromosome sequences using IGV browser (26) and comparing profiles from the mncRNA^+TAP^, mncRNA^−TAP^ and sRNA libraries (Supplementary Figure S1B). Potential candidates were retained when mapping on annotated intergenic or intronic genomic sequences. Retained candidates had:

1. At least two reads with a clear cut common extremity and at least one read with 100% match to the genome sequence.
2. A significant level of accumulation i.e. >0.1 transcripts per million (TPM), representing a number of raw reads much above the background level.

Candidates were discarded when:

1. Mapping on annotated ncRNAs: rRNAs, tRNAs, snRNAs and U3 snoRNA.
2. Mapping on or close to CDS regions. However, a few exceptions were considered, occurring on mis-annotated genes.
3. Mapping on repeated sequences, usually corresponding to 10–15 copies of truncated ‘snoRNAs that might represent ‘dead snoRNAs,’ products of retrotransposition (27).

### Quantification of mncRNA abundance

FeatureCounts (28) was used to quantify reads mapping on our candidates. Parameters are given in Supplementary File 1. Raw count values were normalized into TPM using the equation:

$$\begin{eqnarray*}{\rm TPM} = {10^6} \times \frac {{\rm reads\, mapped\, to\, transcript/\,transcript\, length}} {{\rm Sum\, ( {reads\, mapped\, to\, transcript\, /\,transcript\, length}} )} \end{eqnarray*}$$

### Identification of Ot–snoRNAs and their targets

The mncRNA candidates were screened to identify the Ot–snoRNAs. In a first approach we used Infernal 1.1 program (29) to identify conserved snoRNAs families by searching for RNA homologies in the Rfam database (30).

In a second approach we used snoScan and snoGPS programs (31) accessible in the corresponding servers that identify C/D and H/ACA box snoRNAs predicting their rRNA or snRNA targets (default parameters were used). We also used SnoReport (32) that predict snoRNAs structures, but do not predict targets. All H/ACA box snoRNAs predicted by snoReport were further checked for their secondary structures using RNA folding form (version 2.3 energies) available in the UNAFold web server (33) using default parameters.

In a third approach we made a visual analysis of all our candidate mncRNA sequences that were not recognized by the previous approach, checking for the basic feature of a C/D box snoRNA: the presence of terminal C- and D-like boxes flanked by strong terminal IR. These IR are usually truncated by trimming of 5′ and 3′ ends during snoRNA maturation and were fully visible only when considering the genomic sequences. Using a similar approach, we searched for H/ACA elements, the ANANNA hinge box and a terminal ACA located 3 nt from the 3′end of the Ot–ncRNA, and verified the predicted secondary structure, i.e. two stem H/ACA box snoRNA folds using RNA folding form program with default parameters (33).

Ot–snoRNA target residues on *O. tauri* rRNA or snRNAs were identified by three distinct approaches:

- Using snoScan and snoGPS programs predicting C/D box and H/ACA box snoRNA structures and their rRNA or snRNA targets (31), with default parameters. For snoScan, we allowed for one base divergence from the strict C or D box RUGAUGA or CUGA consensus.
- Identification of functional orthologs in other species with similar ASE targeting the corresponding rRNA or snRNA residues reported in the snOPY orthological database (34).
- Finally, for the remaining orphan C/D box snoRNAs, we made a manual search for a potential ASE, with a minimum of 10 bases complementary to an *O. tauri* rRNA or snRNA sequence adjacent to D or D’ boxes.

### Analysis of Ot–snoRNA conservation

We used Infernal program to find structural homologs in the Rfam database (30). Functional orthologs in distant species were found by BLASTN of Ot–snoRNAs versus human, yeast and plant snoRNAs using the snOPY database (34), to identify those with a similar ASE sequence to the *O. tauri* ASE targeting the corresponding rRNA or snRNA residue. Finally, we used BLASTN to identify orthologs in several algal genomes showing a significant level of conservation with the Ot−ncRNAs. Sequence similarities were identified by BLASTN with an *e*−value threshold of 10^−5^.

### Northern blot analyses

Northern Blot analysis were performed by loading 2–6 μg of total RNA on 10% polyacrylamide gels (7 × 11 cm). DNA oligonucleotides of 25, 50, 75 and 100 nt, and U1 (163 nt) were used as size markers. RNAs were subsequently transferred to NX membranes (GE−Healthcare) by electrophoretic transfer using the Mini *Trans*−Blot tank from Bio-Rad's modular Mini−PROTEAN Tetra system. Membranes were hybridized overnight with oligonucleotides probes (Supplementary Table S7) in ULTRAhyb^®^−Oligo Buffer (Applied Biosystems). Probes were labeled with γ−^32^P−ATP (Perkin−Elmer) using T4 Polynucleotide Kinase (Promega^®^). Membranes were then washed twice at 50°C for 15 min, first in low stringency buffer (2× SSC (saline-sodium citrate)—0.1% sodium dodecyl sulphate (SDS)) and second in High Stringency Buffer (0.1% SSC—0.1% SDS), and exposed to X-ray films (Amersham). All films were exposed 15 h without intensifying screen, unless otherwise indicated.

## RESULTS

### Production of ncRNAs and sRNAs transcriptomes

Exponentially growing *O. tauri* cultures partially synchronized by a light/dark culture cycle (35) were used to prepare the size-selected RNA fractions. To be exhaustive and include ncRNAs that could be differentially expressed at different stages of cell division, cells were sampled at four different times corresponding to cell populations enriched in distinct phases of the cell cycle (Figure 1A). Subsequently, these four samples were pooled together, producing a single batch of total RNAs, which was then run on polyacrylamide gels to recover 50−300 bases and 19−50 bases long RNAs. These RNA fractions were subsequently used for further cloning steps and identification of mncRNA and sRNAs, respectively (Figure 1B, see ‘Materials and Methods’ section).

Eukaryotic ncRNAs have different 5′ and 3′ termini depending on their biosynthetic pathways: they can have a 5′ CAP or 5′P and a 3′polyA tail or 3′OH (36,37). Construction of transcriptome libraries from ncRNAs requires RNA ligation of adaptors to their 5′P and 3′OH termini. Capped RNAs, mostly synthesized by RNA pol II, but some by RNA pol III (see below), cannot be used directly for cDNA synthesis. They first need to be treated with Tobacco Acid Phosphatase (TAP), which cleaves off the 5′ CAP leaving a 5′P RNA end (38). The mncRNA fraction was therefore divided in two: a mncRNA^+TAP^ fraction that was treated with TAP, and a mncRNA^−TAP^ fraction that was not treated with TAP (Figure 1B). Paired-end-orientated cDNA libraries were thus produced and sequenced to produce three transcriptomes: the mncRNA^+TAP^, the mncRNAs^−TAP^ and the single-end read library of sRNAs. Two biological replicates were done for each library.

### Screening of the libraries and selection of Ot−ncRNA candidates

Reads were mapped to the *O. tauri* genome (see ‘Materials and Methods’ section). As controls we inspected the reads mapped to the annotated mncRNA genes, including 41 tRNAs, the snRNAs U1, U2, U5 and U6 and the snoRNA U3, the latter being the only snoRNA annotated in *O. tauri* genome. All of them produced distinctive read profiles on the annotated genes, as shown for the snRNAs and U3 (Supplementary Figure S1A). However, U4, an essential snRNA for splicing, was absent from our analysis because it was not present in the assembly of the publicly available V2 of the genome (22). We found it recently in a Pac Bio assembly of the *O. tauri* genome which is currently being analyzed (unpublished data). The sequence of all *O. tauri* snRNAs, including U4, is given in Supplementary File 2.

Given that the genome of *O. tauri* is very small and compact, screening for the candidates was possible by ‘walking’ along the chromosomes, visualizing all the reads profiles. Comparison of mncRNA^+TAP^ versus mncRNA^−TAP^ distinguished capped from non-capped RNAs. Comparison with sRNA libraries, usually mapped on the 5′ and/or 3′ end of the mature mncRNA candidates gave strong support and gave important information on the relative stability of the mncRNA candidates (Supplementary Figure S1B).

Using this approach and applying stringent criteria for the choice of read profiles to be studied (see ‘Materials and Methods’ section) we finally retained 264 candidates displaying clear read profiles. Using tRNAScan-SE web server (39) and BLASTN to align candidates against the plant tRNAs reported in PlantRNA database (40), five other candidates representing non-annotated tRNAs (41) were discarded, reducing the final list to 259 novel mncRNA candidates (Supplementary Table S1).

### Identification and characterization of the Ot–snoRNA family

Within the 259 mncRNA candidates, we identified 131 snoRNAs, which we called Ot–snoRNAs (Supplementary Table S2). Using softwares dedicated to prediction of C/D box and H/ACA snoRNAs (see ‘Materials and Methods’ section), coupled to a visual inspection of all the candidate sequences, we identified 95 C/D box snoRNAs, and 35 H/ACA box snoRNAs (Supplementary Table S3 and Files 3–4). Using different approaches to predict their targets (Supplementary Table S4, ‘Materials and Methods’ section) 111 Ot–snoRNAs were predicted to target rRNA residues, including five which target both an rRNA and an snRNA residue (Supplementary Table S4). Five other Ot–snoRNAs were found to target only snRNA residues (Supplementary Table S4) and therefore could be considered as potential scaRNAs (7). No targets could be predicted for the 17 other candidates, which are therefore collectively called ‘orphan’ snoRNAs (12,13).

Finally, using the Infernal software (29) we searched for conserved RNA families reported in the Rfam database (30) and Ot−snoR109, hereafter called Ot–MRP, was identified as the homolog of the MRP snoRNA (Supplementary Table S3). This RNA is a highly conserved snoRNA implicated in pre-rRNA processing in all eukaryotes, distinct from C/D box and H/ACA box snoRNAs (42).

### Genomic organization of Ot–snoRNA genes

Mapping the 131 Ot–snoRNA candidates on the genome revealed very diverse modes of genomic organization (Table 1 and Supplementary Table S3). A first group of 72 snoRNAs were intronic, hosted in protein coding genes. A large fraction of the Ot–snoRNAs were hosted in genes encoding proteins implicated in ribosome biogenesis (33%) or RNA processing (18%) (Supplementary Table S3). All intronic snoRNAs were uncapped in agreement with their processing from excised introns (13,43). The second group includes 49 snoRNAs encoded in 12 polycistronic clusters (Table 1 and Supplementary Table S3). Ten were independent intergenic clusters, including some overlapping small CDS, which were probably wrongly annotated. Two other clusters (clusters 3 and 5), were intronic (Supplementary Figure S2). These clusters would produce a polycistronic snoRNA precursor (pre-snoRNA), processed to liberate the mature snoRNAs (14). In agreement with this mechanism, all *O. tauri* polycistronic snoRNA candidates were uncapped (Supplementary Table S3). Finally, 10 snoRNAs were encoded by monocistronic genes located in intergenic regions, likely expressed from their own promoters. Eight of them were uncapped (Supplementary Table S3) so they were either processed from a pre-snoRNA precursor produced by RNA Pol II or they were transcribed by RNA pol III. Two others, Ot−CDsno68 and Ot−CDsno69, were capped (Supplementary Table S3). It is likely that Ot−CDsno68 is transcribed by RNA pol II. In contrast, the presence of a T−stretch, an RNA pol III terminator (44), at the 3′end of the Ot−CDsno69 gene locus (Supplementary Table S3) strongly suggests that this is transcribed by RNA pol III in a similar way to human snRNA U6 and the plant snoRNA U3, which both have a 5′ cap structure and are transcribed by RNA pol III (45,46).

**Table 1.** Genomic organization of Ot–snoRNAs

Genomic organization	Number of snoRNAs
Intronics	72
Clustered	49*
Intergenics	10
Total	131

(*): Indicates Ot–snoRNAs found in 12 distinct polycistronic clusters.

### Comparative analysis of Ot–snoRNA accumulation

**Figure 2.**
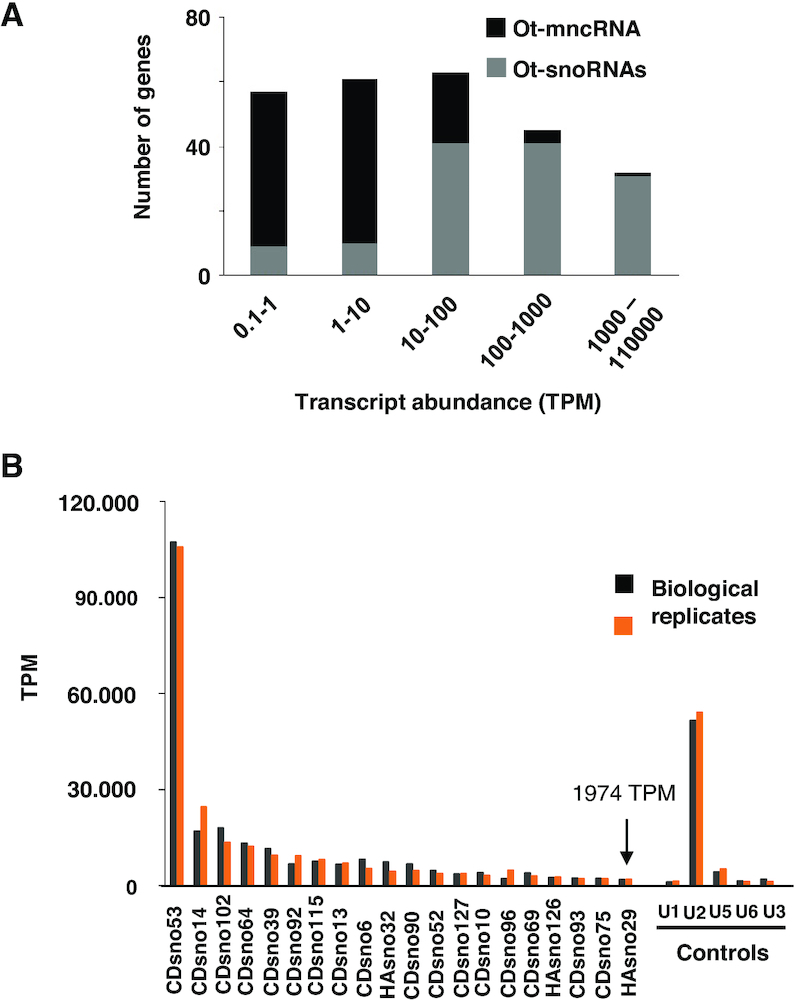
Global analysis of mncRNA abundance. (**A**) Transcript abundance is expressed in TPM. The number of TPM represents the average of replicates 1 and 2 from mncRNA+TAP libraries. (**B**) The top 20 most highly expressed mncRNAs, all corresponding to Ot–snoRNAs. TPM are given for each biological replicate individually. 1974 TPM indicates the average of both replicates for the last member of the top 20 list (black arrow). U1, U2, U5 and U6 are conserved snRNAs implicated in splicing. U3 is the only snoRNA annotated in *Ostreococcus tauri* genome.

We estimated the levels of accumulation of Ot−ncRNAs by comparing normalized read counts expressed in TPM (Supplementary Table S1). Ot–snoRNAs were clearly the most highly expressed candidates (Figure 2A) and included the top 20 most abundant ncRNAs (Figure 2B). The latter appeared to accumulate even more than the snRNAs implicated in splicing (Figure 2B) which are considered to be among the most abundant ncRNAs in eukaryotic cells, together with tRNAs (47). Remarkably, this analysis revealed very large differences in the accumulation levels of snoRNAs. An extreme case was Ot−CDsno53, a C/D box snoRNA, with more than 100,000 TPM, in large excess compared to all other snoRNAs (Figure 2B).

**Figure 3.**
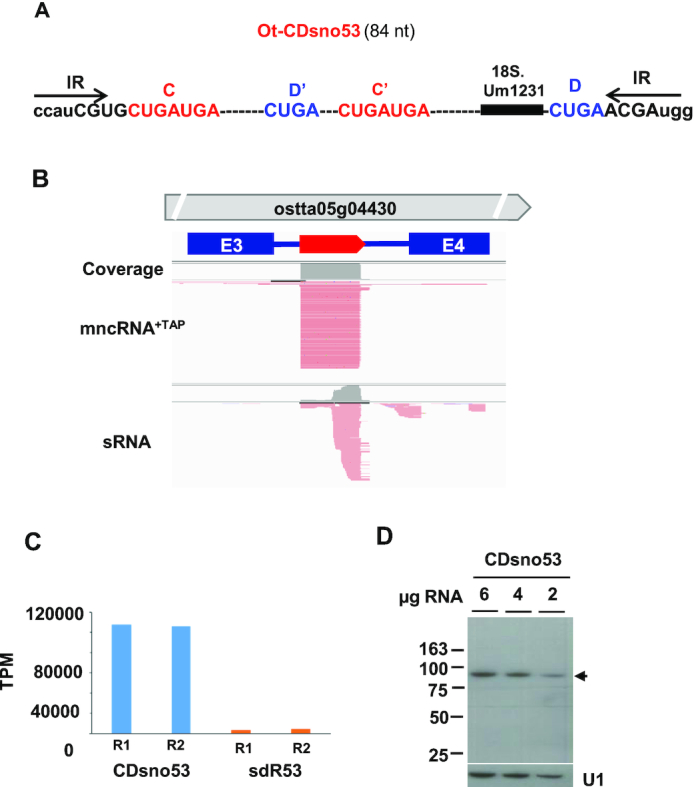
Genomic organization, structure and abundance of Ot−CDsno53. (**A**) Ot−CDsno53 structure. C, D, C’ and D’ boxes elements are indicated in red and blue capital letters. Inverted repeat sequences (IR) are indicated by black arrows. Upper case letters represent the sequences covered by the reads. Lower case letters indicate nucleotides absent from the mature end of the snoRNA. The black bar shows the antisense rRNA element including the indicated target residue. (**B**) Genomic organization and read profile of Ot−CDsnoR53. The thick red arrow indicates the position and the orientation of Ot−CDsnoR53. The thick gray arrow indicates the host gene. Blue rectangles E3 and E4 indicate the third and fourth exons flanking the third intron of this gene. The reads from the mncRNA+TAP and sRNA libraries were visualized with IGV (25). (**C**) Normalized read counts (TPM) of Ot−CDsnoR53 and sdR53 corresponding to replicates (R1 and R2) from mncRNA+TAP (blue bars) and sRNA (orange bars) libraries, respectively. (**D**) Northern blot analysis of Ot−CDsnoR53 abundance. The amount of total RNA loaded on the different lanes are shown at the top of each track. The blot was exposed with an autoradiographic film for 15 h. The snRNA U1, detected with a specific probe, was used as a control. The black arrow indicates the expected position of Ot−CDsno53. Length markers in nucleotides are indicated on the left.

We strove to explain the excessive levels of Ot−CDsno53 compared to all other C/D box snoRNAs. Ot−CDsno53 is an intronic snoRNA hosted in the ostta05g04430 gene which encodes a protein with a predicted MATH/TRAFvdomain (22). It is an 84 nt snoRNA with a canonical C/D box structure predicted to target methylation of the 18S.U1231 (Figure 3A). Its read profile precisely delimits the 5′ and 3′ ends of Ot−CDsno53 mapping on the terminal IR (Figures 3A and B), as expected for the mature ends on the C/D box snoRNAs (2,13).

The profile of sRNAs reads mapping on Ot−CDsno53 locus, here called sdR53, (for sno-derived R53) revealed the accumulation of 3′ end fragments, likely produced by Ot−CDsno53 degradation. We observed that the ratio of Ot−CDsno53 versus sdR53, a relative measure of its susceptibility to degradation, was very high (27.7) (Figure 3C), indicating that Ot−CDsno53 is highly stable.

**Figure 4.**
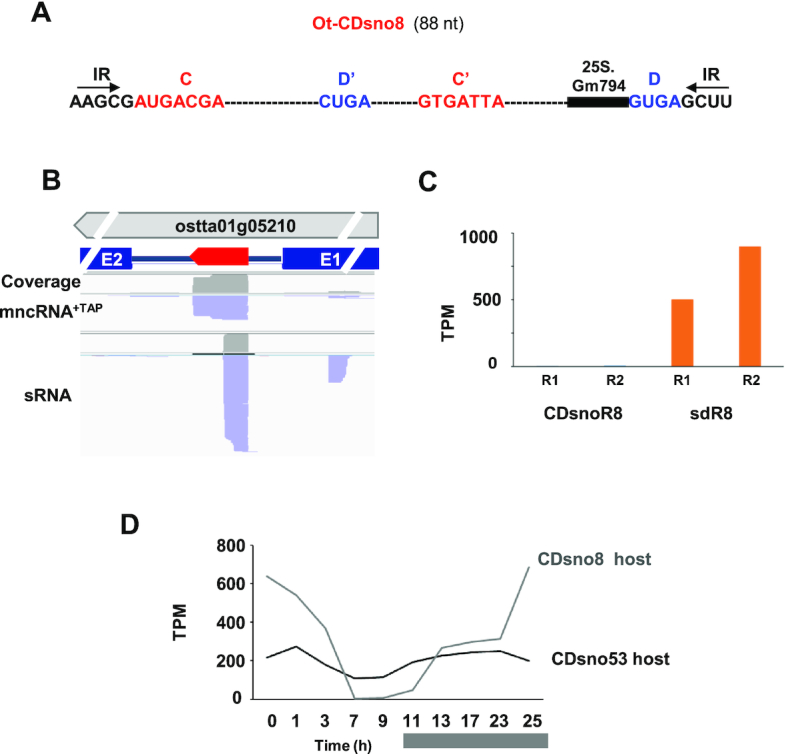
Genomic organization, structure and abundance of Ot−CDsno8. (**A**) Ot−CDsno8 structure. (**B**) Genomic organization and read profile of Ot−CDsno8 (thick red arrow). The thick gray arrow indicates the host gene. Blue rectangles indicate the exons flanking the intron. (**C**) Normalized read counts (TPM) of Ot−CDsno8 and sdR8 replicates (R1 and R2) from mncRNA+TAP (blue bar) and sRNA (orange bar) libraries, respectively. (**D**) Ot−CDsno53 and Ot−CDsno8 host gene mRNA expression during a 24 hr cell culture growth cycle. The dark bar indicates the night period (data from reference 23).

In stark contrast to Ot−CDsno53, Ot−CDsno8, an intronic CD snoRNA nested in the ostta05g05210 gene (Figure 4A and B) was accumulated at a very low level (∼3.5 TPM) (Figure 4C). Notably the number of sdR8 reads was much higher than sdR53 and the ratio of Ot−CDsno8 versus sdR8 was very low (0.005) (Figure 4C), revealing Ot−CDsno8 to be unstable.

Ot−CDsno8 has the features of C/D box snoRNAs, i.e. the C and D boxes flanked by the terminal IR where the 5′ and 3′ ends were clearly mapped, as internal C’ and D’ boxes and an ASE element targeting an rRNA residue (Figures 4A and B). However, we note that the terminal stem of Ot–CDsno53 of 7 bp, is very strong, compared to most of C/D box terminal stems, which is the 4 bp stem of Ot–CDsno8. And terminal stems are important structural elements for assembly of the snoRNP, that protects the snoRNA from 5′ and 3′ exonucleolytic degradation (3). This suggests that small structural differences between these two intronic C/D box snoRNAs, of similar sizes and both targeting rRNA residues, have a critical impact on their differential stability.

**Figure 5.**
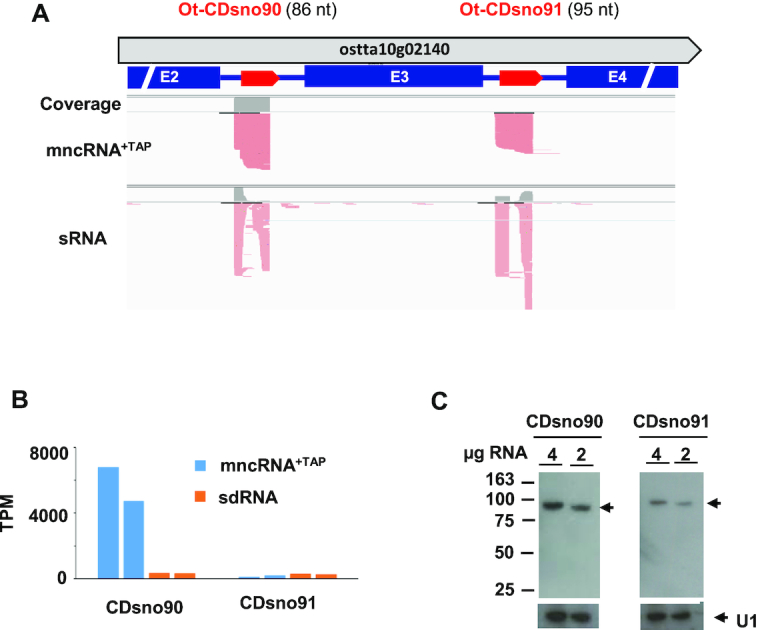
Genomic organization and abundance of Ot−CDsno90 and Ot−CDsno91. (**A**) Intronic localization of Ot−CDsno90 and Ot−CDsno91. The thick red arrows indicate the snoRNAs. The thick gray arrow indicates the host gene. Blue rectangles indicate the exons flanking the introns. (**B**) Normalized read counts (TPM) of Ot−CDsno90 and Ot−CDsno91 replicates (blue bars) and to sdR90 and sdR91 (orange bars) respectively. (**C**) Northern blots analysis using specific probes to assess the abundance of Ot−CDsno90 and Ot−CDsno91, respectively (See Figure 3D for details of labeling).

**Figure 6.**
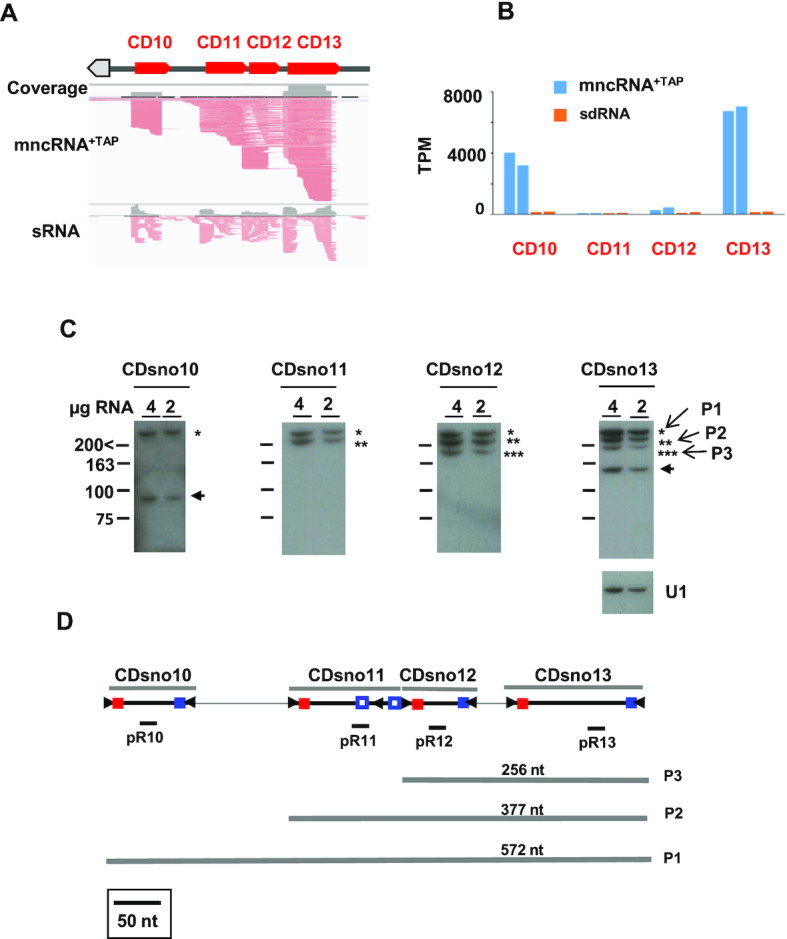
Genomic organization and expression of polycistronic cluster 1. (**A**) Organization and read profile of cluster 1. The thick red arrows indicate the position and the orientation of the clustered Ot–snoRNA. (**B**) Normalized read counts of the 4 Ot–snoRNAs predicted in cluster 1. TPM corresponding to replicates from mncRNA+TAP (blue bars) and sRNA (orange bars) libraries are shown, respectively. (**C**) Northern blot analysis of Ot–snoRNAs from cluster 1. Conditions were similar to Figure 3D, with the following exceptions. The blots were hybridized with four different probes (indicated in panel 3D) to detect the predicted mature Ot–snoRNAs. The black arrows indicate the position of the predicted mature Ot–snoRNAs. P1 (*), P2 (**) and P3 (***) indicate the intermediate products, schematized in panel **D**. The length marker 200 < indicates that the length of size bands longer than 200 nts could not be determined precisely in the 10% polyacrylamide gel used here (see ‘Materials and Methods’ section). All northern blots were exposed for 15 h without intensifying screening except for Ot−CDsno10 which was exposed with an intensifying screen. (**D**) Detailed structure of cluster 1 drawn to scale. The position of mature Ot−CDsno10 to Ot−CDsno13 predicted from their read profiles are indicated. Red and blue filled squares indicate C and D boxes, respectively. Open squares represent divergent D’ boxes. Black filled arrowheads represent Inverted Repeats flanking terminal C and D boxes. Note that Ot−CDsno11 has a divergent CD−box snoRNA structure. The positions of the different probes (pR10–pR13) are indicated. P1, P2 and P3 represent the predicted ‘intermediates’ revealed by the northern blot.

The cellular abundance of ncRNAs based on transcriptomic data can in some cases substantially differ from their abundance measured by northern blot due to low processivity of reverse transcriptase on highly structured RNA templates affecting cDNA synthesis (47). We assessed the level of accumulation of Ot−CDsno53 and Ot−CDsno8 in total RNA extracts by northern blots using specific probes. A very strong signal of the expected size was detected with Ot−CDsno53 probe (Figure 3D). It confirmed the very high accumulation of this candidate. However, its level was comparable, but not higher than the level of accumulation of other snoRNA candidates, like Ot−CDsno90 (Figure 5C) and Ot−CDsno13 (Figure 6C), both included in the top 20 most abundant snoRNAs (Figure 2B). This indicates that the level of Ot−CDsno53 was overestimated by transcriptomic quantification, may be due to ‘PCR overamplification’ produced by some transcripts (48). No signal was detected with a specific probe for Ot−CDsno8, even after several days of exposure with an intensifying screen in concordance with its low level of expression (result not shown).

U1, a highly structured snRNA, is among the most abundant ncRNAs in eukaryotic cells (36,47). We compared the level of Ot−CDsno53 to *O. tauri*’s U1, of 163 nt in length. The northern blot showed that U1 was also highly abundant, the signal intensity being even slightly stronger than Ot−CDsno53 (Figure 3D), contradictory to U1 quantification of reads (Figure 2B) and clearly showed that its level of accumulation was underestimated, as it occurs for highly structured RNAs with strong secondary structures (47).

Northern blot analysis confirmed however the very high abundance of Ot−CDsno53, contrasting with the low level of Ot−CDsno8. Considering that the expression of an intronic snoRNA depends on the expression of its host gene, we compared the levels of expression of Ot−CDsno53 and Ot−CDsno8 host genes respectively, using published *O. tauri* mRNA transcriptomic data (23). Cultures and RNA extractions for the mRNA transcriptomes were done in the same conditions as those used to prepare Ot−mncRNA transcriptomes. Expression profiles, analyzed over 25 h, including a day and night period, revealed they were both expressed at a moderate and comparable level, even though these genes had different expression profiles (Figure 4D). Clearly, no correlation could be made between the high accumulation Ot−CDsno53 and the expression level of its host gene, which is only moderately expressed. Overall, these data suggest that high stability is important for Ot−CDsno53 abundance. This hypothesis was reinforced by our observation that globally, most abundant C/D box Ot–snoRNAs (more than 500 TPM) have the highest snoRNA/sdRNAs ratios (>5-fold), while the bulk of C/D box Ot– snoRNAs have lower snoRNA/sdRNAs ratios (Supplementary Figure S3).

### Expression of intronic snoRNAs

We extended this analysis by comparing the accumulation levels of different Ot–snoRNA candidates hosted in consecutive introns of the same host gene. Ot−CDsno90 and Ot−CDsno91 are two C/D box snoRNAs nested, respectively, in the second and third introns of ostta10g02140, a gene predicted to encode a nucleoporin (Figure 5A). Remarkably, although both are encoded at equimolar levels in the pre-mRNA precursor, Ot−CDsno90 was highly accumulated (∼5733 TPM) whereas Ot−CDsno91 was much less abundant (161 TPM) (Figure 5B). Northern blot with specific probes to each of them gave signals from single bands at their expected sizes (Figure 5C). Based on signal intensities Ot−CDsno90 would be much more abundant than Ot−CDsno91 (Figure 5C). Differences of abundance between those two snoRNAs correlates with a high ratio (17.4) of Ot−CDsno90/sdR90 and a low ratio (0.5) Ot−CDsno91/sdR91 (Figure 5B).

A similar result on the differential accumulation of all other snoRNAs (two or three) located on consecutive introns of a host gene was observed. In all cases a higher abundance of a particular intronic snoRNA was correlated with a high ratio of snoRNA over sdRNAs (Supplementary Figure S4).

These data reinforce the hypothesis that the stability snoRNAs is a major determinant of their abundance and is not correlated with the level of expression of the host mRNA.

### Expression of clustered snoRNAs

Next, we compared the abundance of snoRNAs encoded by gene clusters. In plants, yeast and other species transcription of these snoRNA clusters produces a polycistronic snoRNA precursor (pre-snoRNA) which is rapidly processed to liberate the individual snoRNAs (49–51). In *O. tauri*, final mature snoRNAs often exhibited large differences in their accumulation even though they were present at equimolar ratio in the pre-snoRNAs. Cluster 1 encoded four distinct C/D box snoRNAs (Ot−CDsno10 to Ot−CDsno13) predicted on the basis of their read profiles together with the position of C/D boxes flanked by the terminal IRs in the cluster genomic sequence (Figure 6A and D). Ot−CDsno13 is the homolog of U14 which is conserved in all eukaryotes (1,4). Both Ot−CDsno13 (6872 TPM) and Ot−CDsno10 (3593 TPM) were highly accumulated, whereas a lower level was recorded for Ot−CDsno12 (359 TPM) and Ot−CDsno11 (58 TPM) (Figure 6B). The high levels of Ot−CDsno13 and Ot−CDsno10 also correlated high ratios of Ot−CDsno13/sdR13 (45.7) and Ot−CDsno10/sdR10 (23.1), while these ratios were much lower for Ot−CDsno12/sdR12 (3.1) and Ot−CDsno11/sdR11 (0.7) (Figure 6B). A northern blot with specific probes (pR10–pR13) showed a strong signal migrating at the expected size for mature Ot−CDsno13 (Figure 6C). Additional fragments of larger size hybridized with probe pR13, as well as the other probes, and represent additional snoRNA forms produced by cluster 1 (see below).

Ot−CDsno10’s probe also detected a signal at the expected size (Figure 6C). However, no signal was detected for the predicted Ot−CDsno11 and Ot−CDsno12, even after long autoradiogram exposure (Figure 6C). These results confirmed the differential accumulation of Ot−CDsno10 and Ot−CDsno13, in agreement with transcriptomic quantifications. We cannot exclude that Ot−CDsno11 and Ot−CDsno12 also accumulate individually, as suggested by transcriptomic read profiles, but their levels would be below the sensitivity of the northern blot.

A similar result was obtained for other clusters, showing large differences in the accumulation of the different snoRNAs produced from the same polycistronic precursor (Supplementary Figures S5 and 6, data not shown). A global analysis of all Ot–snoRNA abundances versus Ot–snoRNA/sdRNA ratios clearly shows a significant positive correlation (*ρ* = 0.55, *P*-value < 2.2e^−16^) (Supplementary Figure S7A) specially at higher levels of Ot–snoRNAs (>100 TPM) (*ρ* = 0.66, *P* -value < 2.2e^−16^) (Supplementary Figure S7B).

These data revealed the differential accumulation of snoRNAs when nested in pre-mRNA introns or in polycistronic precursors, and further suggested that stability is an important determinant of their abundance in the cells.

### Unusual snoRNAs produced from polycistronic clusters

Northern blot analysis with the pR13 probe (Figure 6D) revealed three larger RNA forms in addition to mature Ot−CDsno13 (Figure 6C). These larger bands could correspond to different snoRNA precursor intermediates, P1, P2 and P3 (Figure 6D). This hypothesis was confirmed by hybridization with Ot−CDsno10, Ot−CDsno11 and Ot−CDsno12 specific probes (Figure 6D). The pR12 probe specifically hybridized to the P1, P2 and P3 RNA forms recognized by pR13 (Figure 6C). The pR11 probe recognized only P1 and P2, while pR10 recognized only P1 and the mature Ot−CDsno10 (Figure 6C). This pattern of hybridization for the different probes fitted perfectly with the snoRNA structural organization predicted by the position of the terminal C/D boxes flanked by the IRs, as proposed for cluster 1 (Figure 6D).

These data confirmed that P1, P2 and P3 may represent distinct polycistronic precursor intermediates of the mature Ot−CDsno16. However, this was surprising given that pre-snoRNA processing is extremely rapid and intermediates of processing do not accumulate in the cells (49–51). In *O. tauri* cells, predicted intermediates were extremely abundant, nearly as much as the mature Ot−CDsno13, which is highly expressed (Figure 6C), revealing that these ‘intermediate precursors’ were stable products.

A similar situation was observed for snoRNAs from other polycistronic clusters. One example was the C/D box snoRNAs Ot−CDsno19 and Ot−CDsno24 encoded by cluster 2 (Supplementary Figure S5A). The northern blot with a specific probe for Ot−CDsno19 (a canonical C/D box snoRNA targeting the rRNA), did not detect a signal at the expected size of 114 nt, but detected a larger fragment (>200 nt) (Supplementary Figure S5C). This could not be predicted based on its read profile (Supplementary Figure S5A) and indicated that Ot−CDsno19 would be rather a longer snoRNA with 5′ or/and 3′ extension, beyond the terminal C/D boxes.

In the case of Ot−CDsno24, a C/D box targeting rRNA, northern blot detected three major products, two large fragments (>200 nt) and a smaller fragment of ∼75−80 nt (Supplementary Figure S5C). One of the large fragments likely represented the mature Ot−CDsno24, which is 208 nt long (Supplementary Figure S5A and D). We could not determine the size of these large fragments precisely due to the low resolution of the upper part of the gel, precluding resolution of size differences. The ∼75 fragment could be a smaller C/D box snoRNA which can be predicted by the reads profile, produced by processing of Ot−CDsno24 (Supplementary Figure S5A). The weaker signal at ∼163 nt corresponds to U1, used as a control (see legend of Supplementary Figure S5D).

A third example is shown for cluster 10. Six Ot–snoRNAs were initially predicted based on the read profile of this cluster (Supplementary Figures S5A and B). However additional ‘subproducts’ were produced by processing of some of the predicted Ot–snoRNAs. One example was Ot−CDsno103 where 3 C/D box snoRNAs were produced according to their reads’ profiles, fitting perfectly with the position of the terminal IRs and C/D boxes in the parent Ot−CDsno103 (Supplementary Figure S6C).

These data clearly showed that polycistronic clusters produce long snoRNAs which accumulate at high levels in *O. tauri*, that can in turn be processed to produce shorter forms of Ot–snoRNAs which accumulate at variable levels.

### The MRP snoRNA

Ot−MRP (246 nt) is the homolog of MRP RNA, a unique snoRNA distinct from C/D and H/ACA boxes snoRNAs. It is an essential subunit of the MRP endonuclease implicated in processing of pre-rRNA precursors (42). Ot−MRP is located in an intergenic region (Supplementary Figure S7A and 8) and it is not capped (Supplementary Table S3) suggesting that it is transcribed by RNA pol III, as in mammals. This is supported by the presence of a T−stretch terminator (44), at the 3′end of the gene (Supplementary Table S3).

The transcriptomic data suggested that Ot−MRP had a low level of accumulation (∼5–10 TPM) (Supplementary Figure S8B) which would be hardly detected by northern blot. This was surprising because this snoRNA is abundant in eukaryotic cells, as most RNA pol III transcripts (47). To resolve this conflict, we made a northern blot of total RNA with a specific probe for Ot−MRP, revealing a strong signal at the expected size (Supplementary Figure S8C). Clearly, the abundance of Ot−MRP is largely underestimated by the transcriptomic quantification, likely due to the strong secondary structure of this conserved snoRNA (42), as previously observed for U1 snRNA (Figure 4A).

### Conservation and predicted functions of *O. tauri* snoRNAs

The extreme differences in Ot–snoRNA abundances might be linked to their relative conservation in other species or to their functions. We thus searched for homologs of Ot–snoRNAs, either by identification of functional homolog in distant species, targeting the same rRNA or snRNA residues reported in snOPY, a snoRNA orthological database (34) or by identification of structural homologs by Infernal/Rfam analysis and by BLASTN alignment of Ot–snoRNA sequences with genomic sequences from algal species (see ‘Materials and Methods’ section).

**Figure 7.**
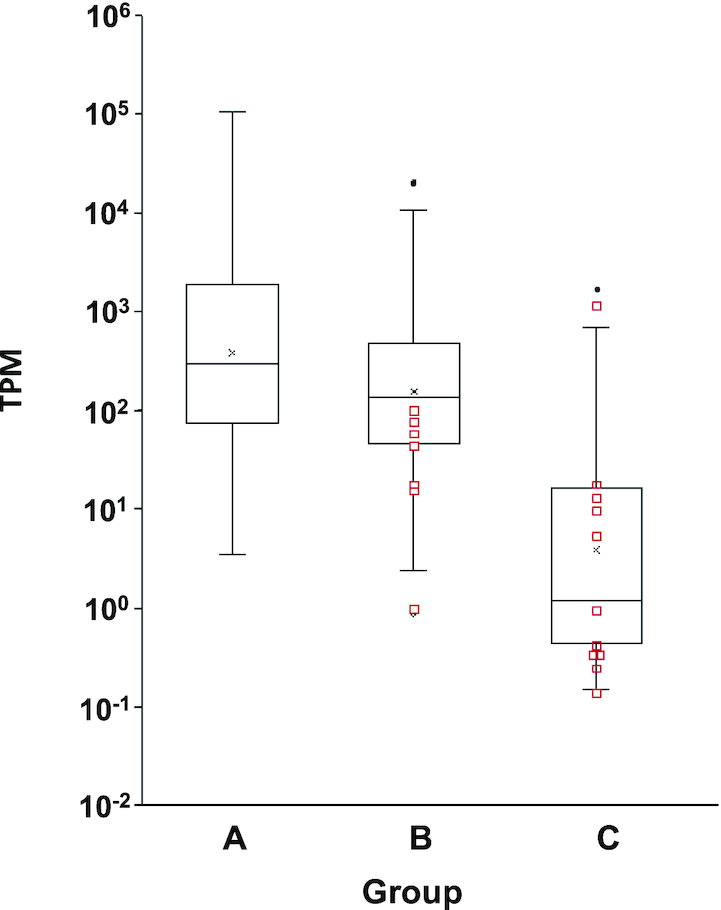
Conservation and abundances of Ot–snoRNAs. Group A: Ot–snoRNAs conserved in distant species (human, yeast and plants) Group B: Ot–snoRNAs conserved in Mamiellophyceae Group C: Ot–snoRNAs specific to *Ostreococcus tauri*. Red small squares indicate position of orphan Ot–snoRNAs. This figure was produced using data extracted from Supplementary Tables S4–6

Three groups, A, B and C, could be distinguished based on their conservation (Figure 7). Group A was composed of 54 Ot–snoRNAs homologs of snoRNAs found in mammals, yeast or plants (Supplementary Table S4). All of them were predicted to target the corresponding rRNA or snRNA residues which are conserved in these species (Supplementary Table S6). This group had the highest levels of accumulation, including 13 belonging to the top 20 most abundant snoRNAs (Figure 7 and Supplementary Table S6). Group B (Figure 7) was composed of 55 Ot–snoRNAs which had homologs in other species of Mamiellophyceae (Supplementary Table S5). Group B has a similar level of accumulation to group A (Figure 7). Among them 7 belonged to the top 20 most abundant Ot–snoRNAs (Supplementary Table S6). Notably, within this group of Mamiellophyceae-specific snoRNAs, seven were orphans, including the abundant Ot–CDsno19 (273 TPM) (Supplementary Table S6). Group C, was composed of 22 Ot–snoRNAs specific to *O. tauri* (Supplementary Tables S5 and 6). Twelve of them were predicted to target rRNA or snRNA and 10 were orphan snoRNAs (Supplementary Table S6). These had a much lower level of accumulation than the conserved Ot–snoRNAs (Figure 7). However, five of them accumulated much above the average level of this group (Supplementary Table S6). In particular, we note the high abundance of the orphan snoRNA Ot–CDsno131, a C/D box snoRNA with an unusual structure (Supplementary File 3).

Taken together, these data revealed a revealed clear differences in the abundances of Ot–snoRNAs that are conserved with other species compared with the Ot–snoRNAs that were found only in *O. tauri*. In addition, it revealed some orphan Ot–snoRNAs specific to Mamiellophyceae or to *O.tauri*, with a high levels of accumulation, suggesting that they may have important roles in these species.

## DISCUSSION

### The snoRNA gene family in *Ostreococcus tauri*

We presented here the first study of the snoRNA family in a marine unicellular alga. We identified 131 novel Ot–snoRNAs, to which must be added U3, the only snoRNA that had been annotated in the *O. tauri* genome (22). Hence, at present Ot–snoRNAs represent the largest family of ncRNAs identified in a marine alga.

Most of Ot–snoRNAs were predicted to target modification of rRNA nucleotides, which are essential for ribosome biogenesis. Their expression must be coordinated with rRNA synthesis. Eukaryotes use different strategies to achieve this coordination. In mammals, most snoRNAs are intronics, with an important fraction nested in genes related to ribosome biogenesis (14). In plants and in the unicellular alga *C. reinhardtii*, most snoRNAs are encoded by polycistronic gene clusters which ensure their coordinated expression (14,15). In yeast, which also has a small compact genome with few introns, most snoRNAs are encoded by intergenic genes driven from their own promoter (14). The coordinated expression of these genes is ensured by the Tbf1, a transcriptional regulator that binds to their promoter (52). Therefore, the organization of the *O. tauri* snoRNAs genes appears to be quite unique among eukaryotes as it combines the intronic organization of animals with the clustered organization of plants (Table 1).

Another distinctive feature of Ot–snoRNAs is that, apart from 3 exceptions, they are encoded by single copy genes (Supplementary Table S2). This substantially differs from plants and *Chlamydomonas reinharditii*, where most snoRNAs are encoded by multigenic families. These arose by extensive gene duplication during evolution, producing multiple snoRNA isoforms targeting new rRNA methylation sites (15,53). In *O. tauri* this did not occur, probably because snoRNA gene duplications were constrained by its small and highly compact genome, nevertheless preserving the ‘essential’ snoRNA complement required to produce the rRNA modification profile necessary for proper ribosome biogenesis. Indeed, the genome size seems to be an important issue in *O. tauri*, the smallest living eukaryote. This might be an important evolutionary adaptation for reduction of its overall cell size, to optimize the surface to volume ratio in this marine phytoplankter. The genome of *O. tauri* is roughly same size as yeast's genome, but has more than 8000 genes, including all the photosynthetic components, versus ∼6000 genes in the yeast genome. This notion is further suggested by the observation that in contrast to most other eukaryotes, where rDNA genes encoding the cytoplasmic rRNA are present in hundreds of copies, more than 150 rDNA copies in yeast (1), in *O. tauri* there are only 3 copies of rDNA genes (22).

In sum, the combination of intronic snoRNAs, many of them nested in genes implicated in ribosome biogenesis or RNA processing (Supplementary Table S3) together with the presence of numerous clustered snoRNAs controlled by a common promoter, was an ‘evolutionary trick’ to optimize the coordinated expression of this essential snoRNA complement at a minimal cost for the development of complex regulatory circuits.

### Beyond the canonical snoRNAs

Northern blots revealed unusual snoRNA structures produced by polycistronic loci, that could not be predicted solely based on their transcriptomic profiles. This was shown for cluster 1 which produces large P1, P2 and P3 forms that accumulate to very high levels (Figure 5). Multiple forms were also observed arising from other clusters (Supplementary Figures S5 and 6)

In yeast and plants, clustered snoRNA genes are transcribed by RNA pol II producing a single ‘polycistronic’ precursor, rapidly processed into individual units (49–51). The high levels of accumulation of P1, P2 and P3 snoRNA precursors suggest they are stable products. This suggest that these longer structures including multiple snoRNAs could have additional functions, beyond the canonical rRNA modification function of the individual snoRNAs released form the polycistronic transcript.

Long C/D and H/ACA boxes snoRNAs, as well as fusion of C/D and H/ACA boxes sno/scaRNAs, with unusual structures, exerting very different functions have been described in animals (12,54). Another remarkable example is the human telomerase RNA which participates in telomere synthesis. This is not a snoRNA, but its 3′end is characterized by a perfect H/ACA box RNA fold which is assembled into an H/ACA snoRNP (55).

The unusual snoRNA forms described here are, to our knowledge, the first examples reported in the green lineage. They reveal the plasticity of the polycistronic genes to generate a highly versatile group of snoRNAs which might have important functions in *O. tauri* and related species.

### Different levels of abundance of Ot–snoRNAs

Quantification of Ot–snoRNA reads revealed an extremely large range in their levels of accumulation (Figures 2A and B). Globally, northern blot analysis supported the transcriptomic data and confirmed the large difference of abundance of the Ot–snoRNAs. Given that snoRNAs have a common basic C/D box or H/ACA box structure, such large differences in transcript abundance of some snoRNAs is puzzling.

The results presented here, focusing on the differential accumulation of C/D box snoRNAs expressed from the same gene host (Figure 5 and Supplementary Figure S4) or from the same polycistronic precursor (Figure 6 and Supplementary Figures S5-6) strongly suggest that stability is an important determinant in the abundance of the C/D box snoRNAs. The large majority of the small RNAs derived from Ot–snoRNAs are likely produced by non-specific degradation of the parent snoRNAs. However, as discussed below, we cannot exclude that a few of them could have a biological function (discussed below).

The differential stability among C/D box snoRNAs is most likely determined by differential rate on snoRNP assembly on the nascent snoRNAs (3,49). Intronic snoRNAs are produced by processing of the intron released by splicing. This process includes 5′ and 3′ exonucleolytic trimming that produce the mature extremities of the snoRNAs (43,56). The nascent snoRNAs are protected from further trimming by its co-transcriptional assembly with the four nucleolar proteins (2–3,13). In the case of C/D box snoRNAs, whether intronic or polycistronic, this process is initiated by binding of the 15.5 kd protein (Snu13p in yeast) to the K-turn motif formed by C and D elements and the terminal stem (2,57). Thus, the final level of the mature snoRNA is established by a kinetic competition between snoRNP assembly and exonucleolytic degradation on the nascent snoRNA (49). A similar process would occur in *O. tauri* which encodes conserved homologs to all 4 proteins forming the C/D and H/ACA boxes snoRNPs, respectively (Supplementary File 5).

### The Ot–snoRNAs and their function in ribosome biogenesis

Most of Ot–snoRNAs were predicted to target modification of rRNA residues. In this context, the excessive expression of some of them, including the top 20 Ot–snoRNAs, is puzzling when compared to most of the other snoRNAs that also target the rRNAs (Figures 2B and 7).

Indeed, a similar observation has been reported for the human snoRNAome, revealing that in human cells the pool of snoRNAs is dominated by a few snoRNAs which are highly abundant (58). Remarkably, seven of the top 20 Ot–snoRNAs (Ot–HAsno1, Ot–HAsno20, Ot–HAsno23, Ot–CDsno44, Ot–CDsno46, Ot–HAsno100 and Ot–HAsno108) are homologous to some of those found in the list of the most abundant snoRNAs in mammals (58).

In humans, 110 residues are modified by *2*′*-O-ribose* methylation and 100 by pseudouridylation, targeted by snoRNAs. In yeast, with a very compact genome, 55 residues are modified by methylation and 44 by pseudouridylation (4). The number of rRNA nucleotides modified in any marine algal species is unknown, as they have not been mapped. However, the Ot–snoRNAs were predicted to target modification of 112 rRNA residues: 101 by methylation and 11 by pseudouridylation. The low number of pseudouridylated rRNA residues is likely not representative, as usually they are similar to the number of methylated residues. This is due to the low number of H/ACA snoRNAs that we identified, as we discarded several potential candidates because their structures were divergent from canonical features and snoGPS, which has stringent criteria, did not recognized them. Anyway, overall these results suggest that the level of rRNA modification in *O. tauri* is considerably higher than in yeast's rRNAs. Indeed, a similar result was predicted in *C. reinharditii*, where 74 C/D box snoRNAs were predicted to target 2′-O-ribose methylation of 96 rRNA residues (15) much higher than the 55 residues methylated in yeast rRNAs (4).

Considering these observations, one possibility is that differential accumulation of Ot–snoRNAs could contribute to ribosome heterogeneity in *O. tauri* cells. Differential expression of snoRNAs in different tissues, or responding to different stimuli, has been observed in higher eukaryotes (13,58). In addition, it has been shown that nucleotide modifications can modulate translation capacity and are an important source for ribosome heterogeneity (1). Furthermore, such rRNA modifications are highly responsive to environmental changes and in response to diseases (4). The scaRNAs targeting modifications of snRNAs, which direct splicing, also show this heterogeneity in their level of modification (59). The differential expression of snoRNAs that target rRNA might therefore be related to regulating the levels of different modifications in the ribosomes of *O. tauri* cells, thereby enabling reactivity to subtle changes in the marine environment.

Finally, an additional explanation would be that the highly abundant snoRNAs (predicted to target rRNAs or scaRNAs) might have additional functions, unrelated to rRNA or snRNA modifications (12,13). One example is human SNORD27, a C/D box snoRNA. This snoRNA has a dual function, targeting both rRNA methylation and regulation of alternative splicing in a pre-mRNA encoding a transcription factor (60). Another important function could be that, in addition to their canonical function, they could be precursor producing small RNAs with regulatory functions. This has been shown in human cells for some snoRNAs that produce miRNAs with regulatory functions (10,11). *Ostreococcus tauri* lacks Dicer and Ago homologs, and hence has no canonical miRNA pathways. However, we cannot exclude that certain small RNAs produced by a Dicer-independent processing of some Ot–snoRNAs, as reported in eukaryotes (12,61–62), might have a regulatory function.

### The orphan Ot–snoRNAs

Similar questions arise concerning the role of the 17 orphan Ot–snoRNAs, for which no rRNA or snRNA predicted target was found. Ten of them are specific to *O. tauri*, while the seven others are found in other species of the class of Mamiellophyceae (Supplementary Table S6). Orphan snoRNAs have been found in several species, and could have functions unrelated to rRNA methylation or pseudouridylation. In the last few years, this was shown for some cases in human or in yeast. Some examples of new functions include targeting acetylation of rRNAs (6), regulation of mRNA alternative splicing (8,60) and control of 3′ end mRNA processing (63). Very recently, in human cells the orphan C/D box snoRD97 and SCARNA97 were found to target methylation of elongator tRNA^Met^, despite their apparent intranuclear localization. Remarkably, this methylation prevented the site-specific cleavage of a tRNA^Met^ which is induced by the stress-responsive endoribonuclease angiogenin, protecting tRNA^Met^ integrity in response to stress (9).

Orphan Ot–snoRNAs that accumulate at significant levels could therefore have important functions in these marine algae, perhaps unrelated to rRNA or snRNA modifications. In particular, this could be the case of Ot–CDsno131, specific to *O. tauri*, which is very highly expressed (Supplementary Table S6).

In summary, the identification and characterization of the Ot–snoRNA family in *O. tauri* has revealed new features of eukaryotic snoRNA expression that permit different levels of accumulation in a minimum of space, thus well adapted to its very small genome. The work presented here will provide a solid base that should be useful for assessing the impacts of environmental changes and viral infections on a major family of ncRNAs marine microalgae.

## DATA AVAILABILITY

The sequence data for the RNA-seq analysis are available in the NCBI sequence read archive with the provisional accession BioProject number PRJNA624281.

## Supplementary Material

lqaa080_Supplemental_Files
