# Supplementary material for: The medium-size noncoding RNA transcriptome of Ostreococcus tauri, the smallest living eukaryote, reveals a large family of small nucleolar RNAs displaying multiple genomic expression strategies
Source: NAR Genom Bioinform. 2020 Oct 9;2(4):lqaa080. doi: 10.1093/nargab/lqaa080 (PMC7671301; doi:10.1093/nargab/lqaa080)
Supplement: lqaa080_Supplemental_Files [file lqaa080_supplemental_files.zip › Supplementary figures S1 to S8.pdf]

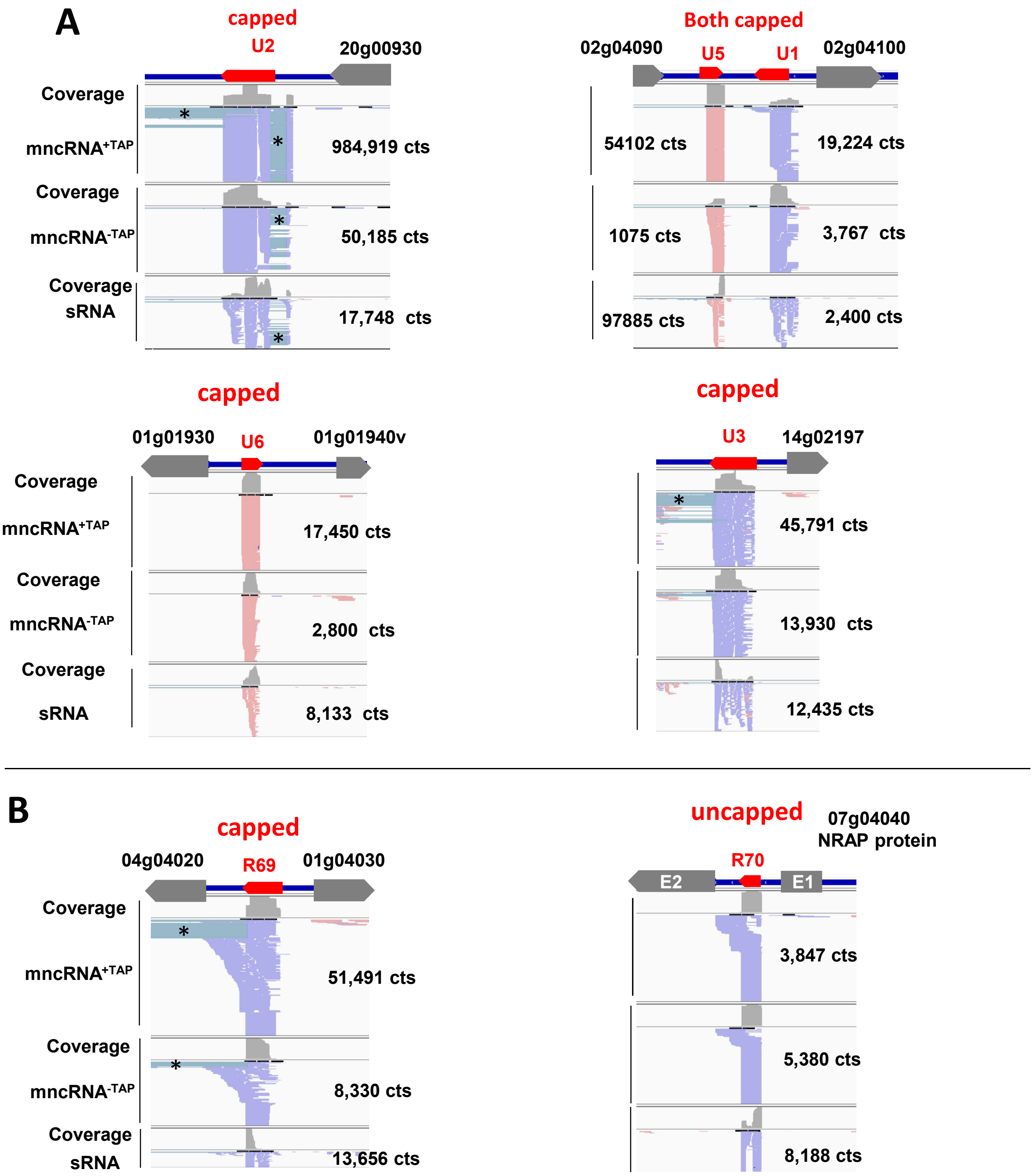

Figure S1: Comparative visualisation of the read profiles and coverages of the 3 libraries used to find mncRNA candidates by chromosome walking.

Flanking genes are shown as thick grey arrows. mncRNA candidates are shown as thick red arrows. Red and blue reads show forward and reverse orientations, respectively. Light blue lines marked with * indicate reads predicted by IGV to span splice junctions separating two reads, likely wrong predictions.

A) Read profiles of control snRNAs and U3 snoRNA.

B) Typical profiles of a capped and an uncapped candidates

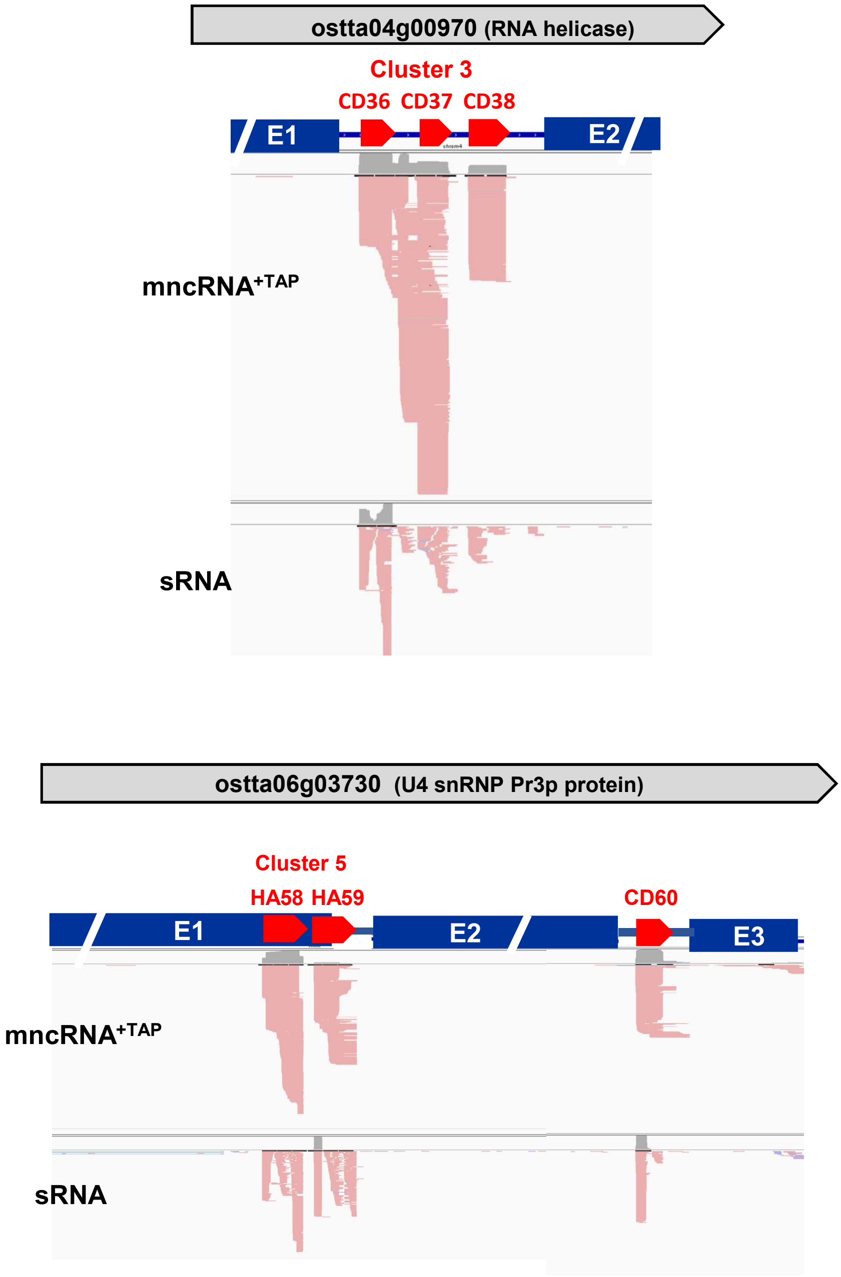

Figure S2: The intronic clusters

The red arrow indicates the predicted Ot-snoRNAs. The grey arrows indicate the host genes. Blue rectangles indicate the exons flanking the introns.

Cluster 3, is hosted in the unique intron of the host gene.

Cluster 5 overlaps with the CDS and the first intron of the host gene. The 2nd intron of this gene encodes Ot-CDsno60.

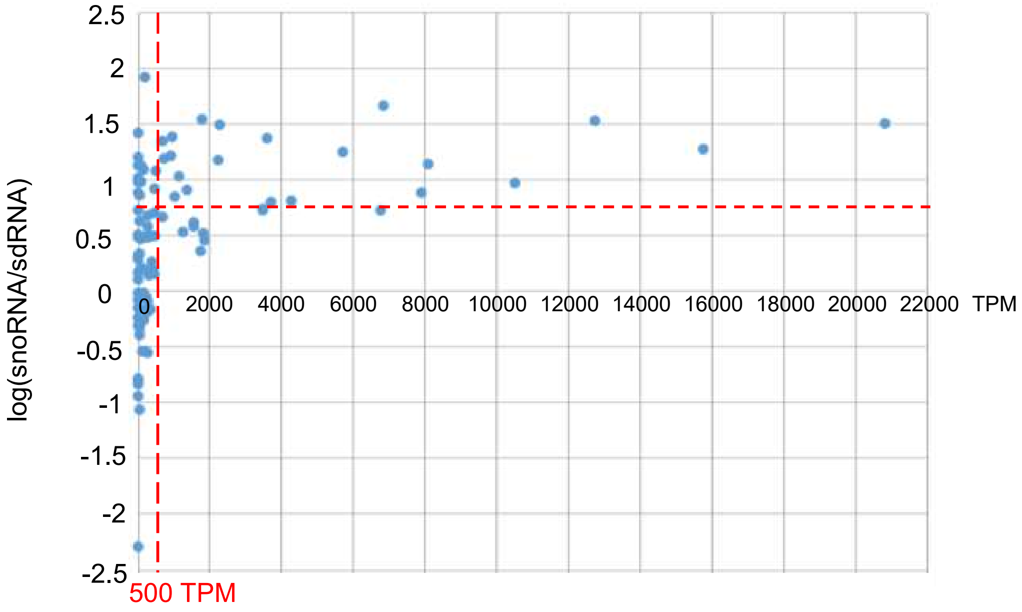

Figure S3: Semi-logarithmic plot of the snoRNA/sdRNA ratios visualised on a $\log_{10}$ scale versus the abundances of C/D box Ot-snoRNAs. The figure shows the same data presented on Figure S6, but focusing on the C/D box Ot-snoRNAs. Ot-CDsno53, (100.000 TPM) is not included in the graph, due to its overestimation by RNA seq (see text). The vertical red dashed line represents 500 TPM. Overall, the most abundant C/D box Ot-snoRNAs have high ratios of snoRNA/sdRNA (Log ratio > 0,7) *i.e.* a 5 fold ratio, indicated by horizontal red dashed line.

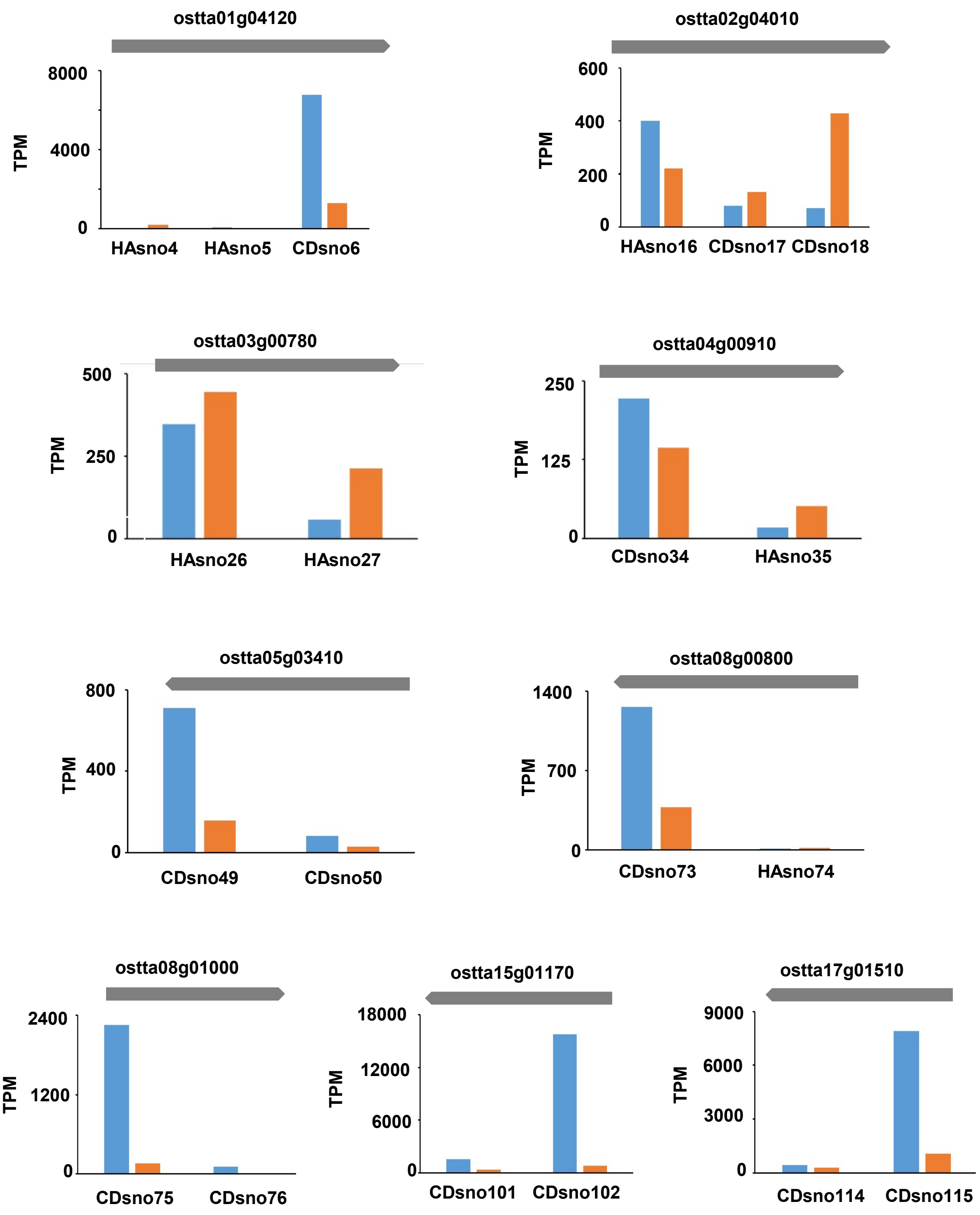

Figure S4: Survey of the abundance of all intronic snoRNAs nested in consecutive introns.

■ **mncRNA^{+TAP}** ■ **sdRNA**

The abundance of reads, expressed normalised in TPM, is reported for all Ot−snoRNAs encoded by consecutive introns in host genes. The grey arrows indicate the orientation of the host genes. Normalised TPM are averages for biological replicates from mncRNA^{+TAP} libraries.

.

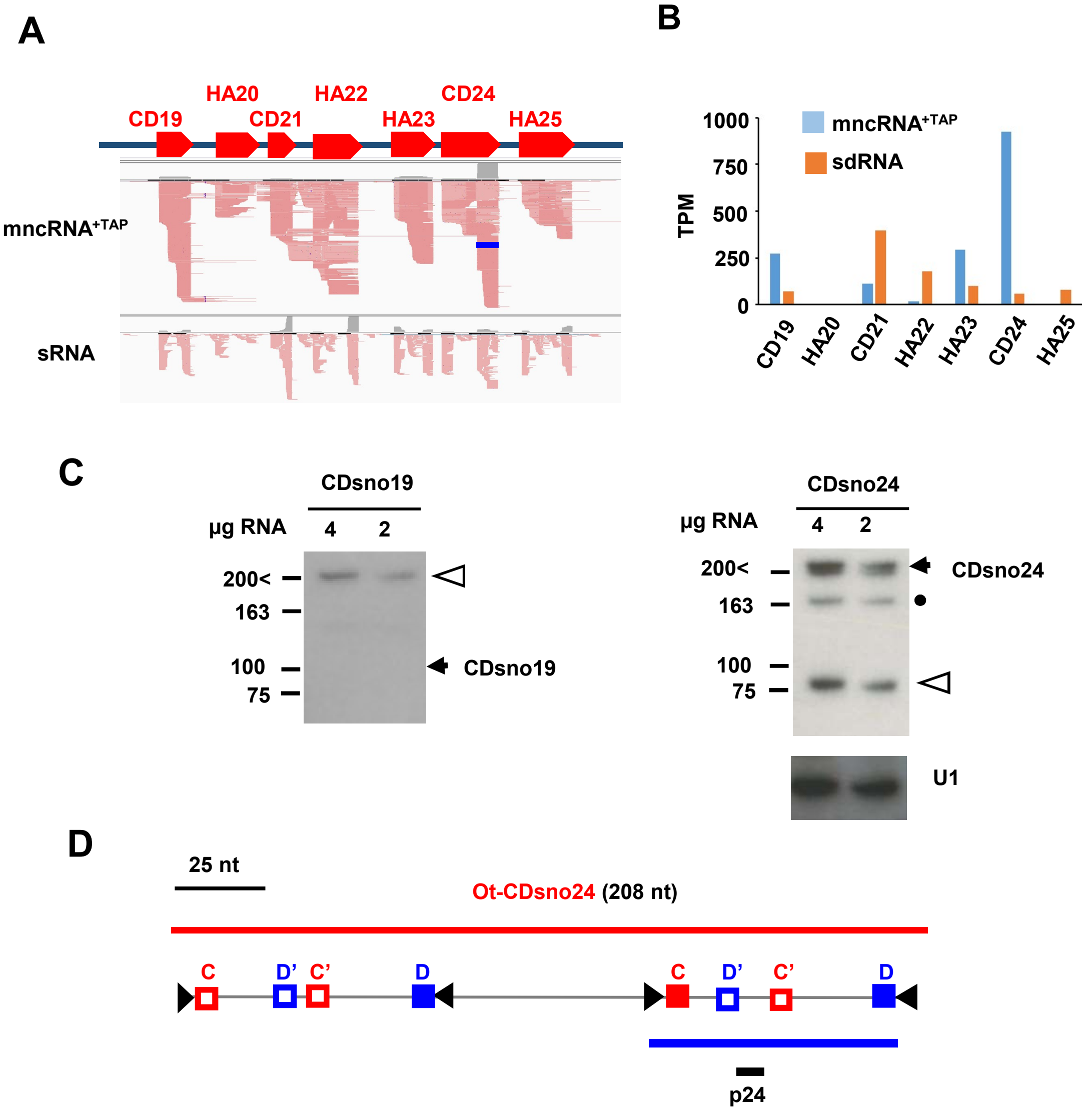

Figure S5. Genomic organization and expression of cluster 2.

(A) Organisation and read profile of cluster 2. The blue bar drawn among the piles of reads on Ot−CDsno24 read profile indicates a snoRNA sub−structure observed in panel D. For more details, see legend of Figure 3B. **(B)** Normalised read counts of Ot−mncRNAs predicted in cluster 2. For details see legend Figure 3C. **(C)** Northern blot analysis of Ot−mncRNAs from cluster 2. For details see legend Figure 3D. Empty arrowheads represent stable products produced from cluster 2, in addition to the predicted snoRNAs. The black dot represents a U1 signal remaining from previous hybridisation of this membrane with a U1 probe. **(D)** Schematic structure of Ot−CDsno24 presenting all C, D and C' D' putative boxes and IRs. The figure is drawn to scale. Red and blue filled squares indicate C and D boxes respectively. Open square represent divergent C, C' and D' boxes. Black arrowheads represent Inverted Repeats flanking terminal C and D boxes, which determine the mature ends of CD-box snoRNAs. The predicted Ot−CDsno24 (thick red line) and short form (thick blue line) correspond perfectly to the red and blue schematised in the read profile visualised in panel A. The position of the probe pR24 is indicated (black line).

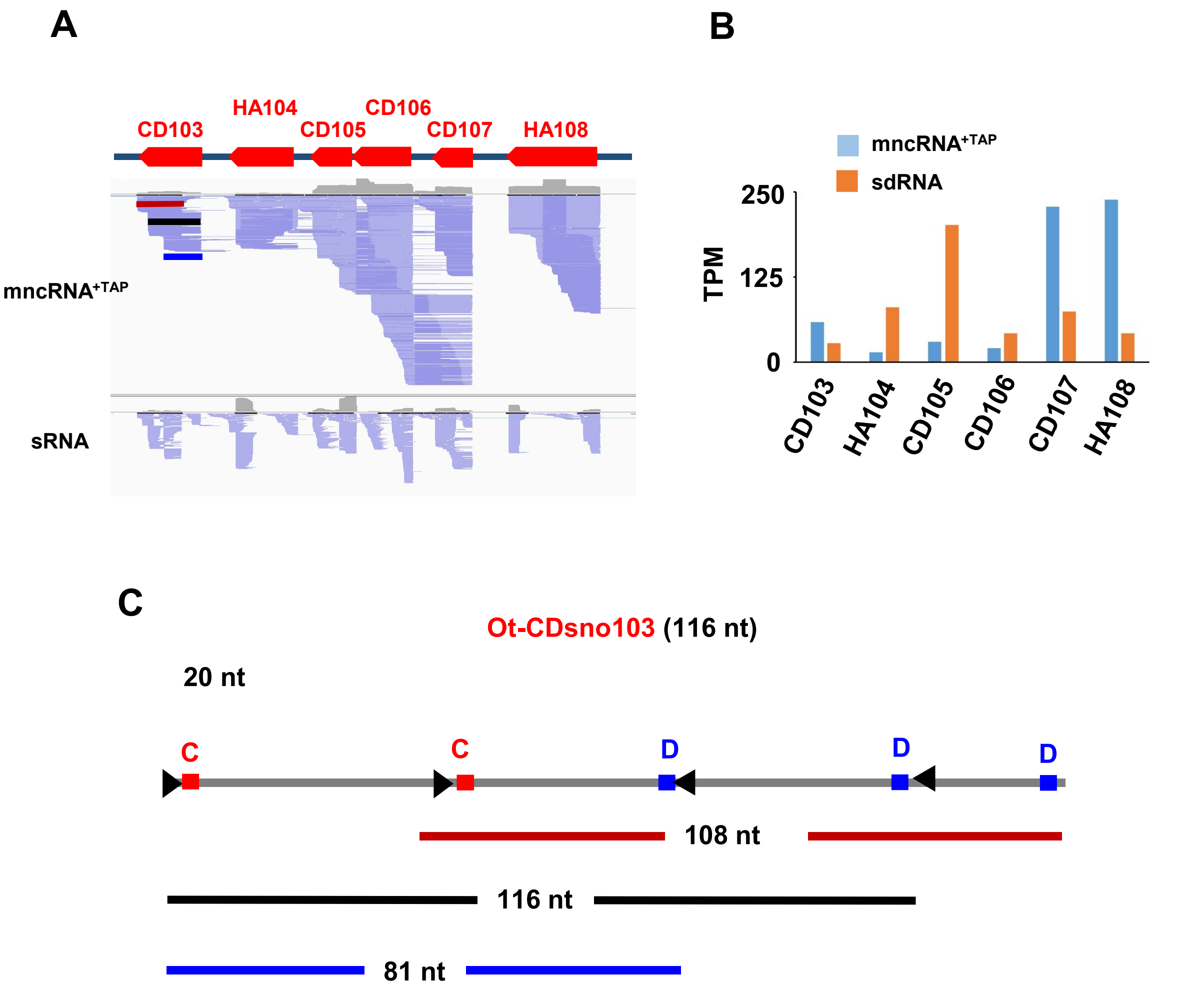

Figure S6: **Genomic organisation and expression of cluster 10**

(A) Organisation and read profile of cluster 10. The red, black and blue bars drawn on the Ot−CDsno103 read profile shows structures derived from this snoRNA presented in panel C. For more details see the legend of Figure 3B. **(B)** Normalised read counts of the Ot−mncRNAs and corresponding sdRNAs predicted in cluster 10. For details see legend Figure 3C. (**C)** Schematic structure of Ot−CDsno103. The black, red and blue lines correspond to read profiles lines shown in panel A.

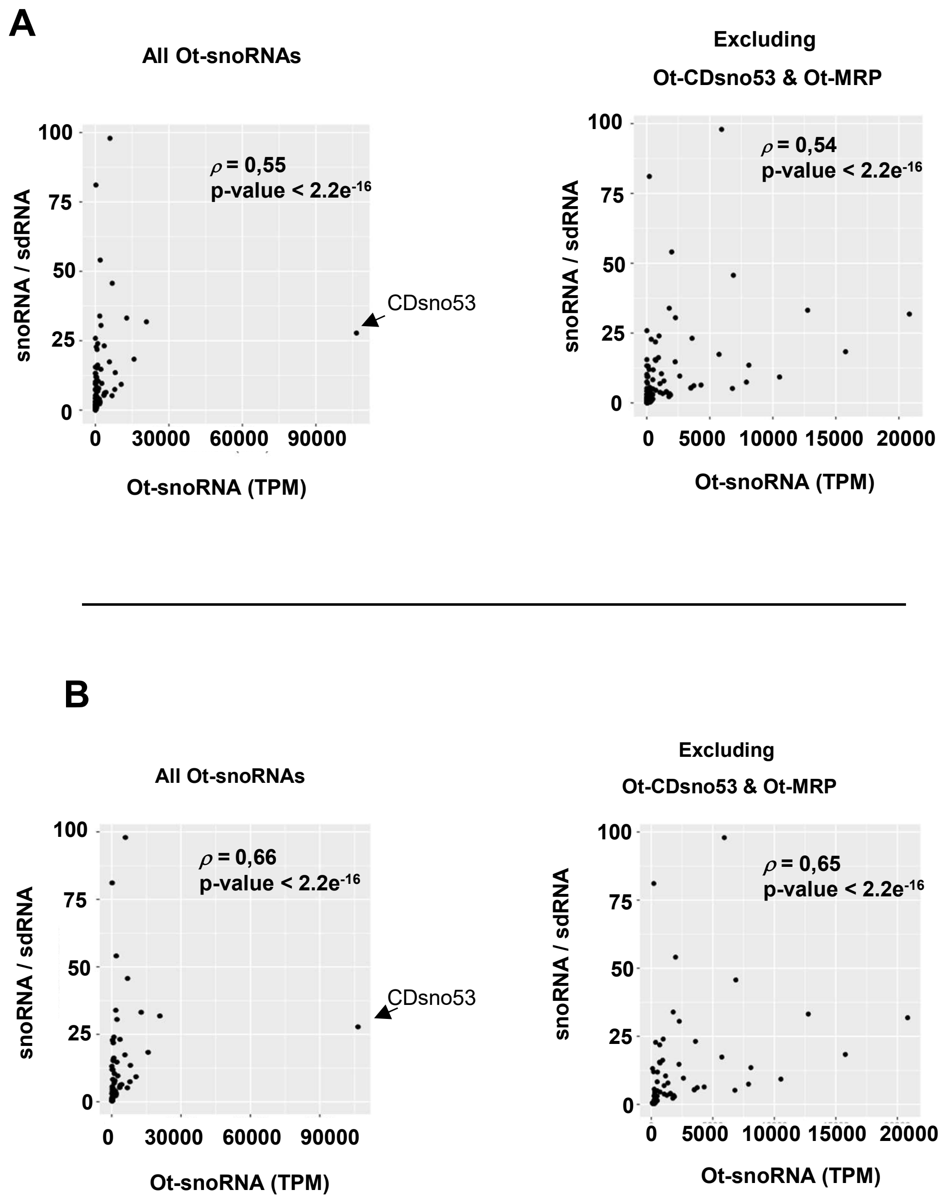

Figure S7 : Correlation of Ot-snoRNA abundances versus the ratio OtsnoRNA / sdRNA.

Ot-snoRNA abundances and ratios Ot-snoRNA/sdRNAs represent averages of both replicates..

A : All Ot-snoRNAs, **B**: Ot-snoRNAs > 100 TPM.

As indicated, left panels include all Ot-snoRNAs and right panels exclude Ot-CDsno53 and Ot-MRP, which we know were erroneously quantified by transcriptomic data (see text).

Spearman's ρ and associated p-value are indicated on each panel.

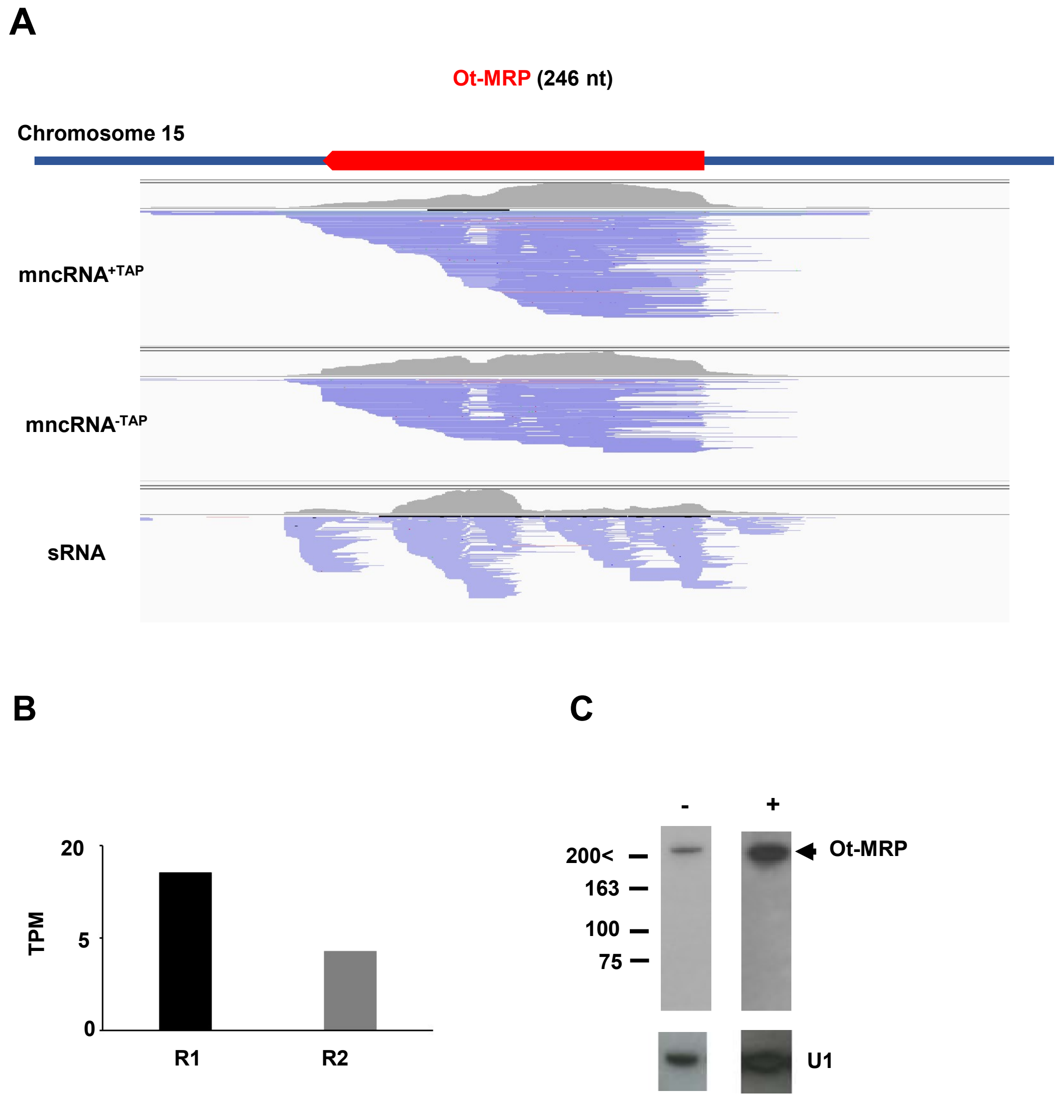

Figure S8: The Ot−MRP, homolog of the MRP RNA

(**A**) Genomic organisation and read profile of Ot−MRP. The red arrow shows Ot−MRP position and the orientation of this candidate. Details in legend from Figure 3B. (**B)** Normalised read counts of Ot−MRP for individual biological replicates R1 and R2 from mncRNA^{+TAP} libraries. (**C)** Northern blot analysis of Ot−MRP expression. Lanes were loaded with 4 µg of total RNAs. (−) and (+) correspond to a 15h film exposure of the blot without and with intensifying screen.
