# Supplementary material for: The medium-size noncoding RNA transcriptome of Ostreococcus tauri, the smallest living eukaryote, reveals a large family of small nucleolar RNAs displaying multiple genomic expression strategies
Source: NAR Genom Bioinform. 2020 Oct 9;2(4):lqaa080. doi: 10.1093/nargab/lqaa080 (PMC7671301; doi:10.1093/nargab/lqaa080)
Supplement: lqaa080_Supplemental_Files [file lqaa080_supplemental_files.zip › Supplementary Files 1 to 5.pdf]

Supplementary File 1: Programs and parameters used in this study

Quality check performed with fastQC
```module load bioinfo/FastQC_v0.11.7``````
fastqc *fastq.gz```

Adapters trimming
Reads were cleaned with Fastq-MCF (from ea-utils, see github repository here: https://github.com/ExpressionAnalysis/ea-utils/tree/wiki).
Example command-lines used:

For HCX 16 & 20
```fastq-mcf small_rna_adapters.fa
150421_SND104_A_L004_HCX-16_R1.fastq.gz -o
150421_SND104_A_L004_HCX-16_R1_Trimmed.fastq.gz```

### For HCX 21,25 & 26, 30
```fastq-mcf small_rna_adapters.fa
150423_SND405_A_L002_HCX-21_R1.fastq.gz
150423_SND405_A_L002_HCX-21_R2.fastq.gz -o
150423_SND405_A_L002_HCX-21_R1_Trimmed.fastq.gz -o
150423_SND405_A_L002_HCX-21_R2_Trimmed.fastq.gz -C 1000000 -q 20 -p 10
-u -x 0.01```

## Read mapping. Performed with STAR (v2.5.2b)

### Genome indexing
``` STAR --runThreadN ${task.cpus} --runMode genomeGenerate --
genomeSAindexNbases 11 --genomeDir STAR_genome --genomeFastaFiles
${genome_file} --outFileNamePrefix STAR_genome --sjdbGTFfile
${annotation_file} --sjdbGTFtagExonParentTranscript Parent```

### Read mapping
#### multimapping reads:  --outMultimapperOrder Random option --> when a read can be aligned to multiple locations on the genome, alignments are reported in random order, and the primary alignment is chosen randomly among the highest scoring alignments. The aim is to filter out multimapping reads, by keeping only the primary alignments.
``` STAR --genomeDir ${STAR_genome} --outFileNamePrefix ${pair_id}_ --
runThreadN ${task.cpus} --readFilesCommand zcat --readFilesIn ${reads}
--outSAMtype BAM Unsorted --outFilterMultimapNmax 12 --
outFilterMismatchNoverLmax 0.2 --outMultimapperOrder Random --
alignIntronMin 15 --alignIntronMax 4000```

### Filtering of multimapped reads with samtools (V1.6)
#### produced alignments files were filtered to keep primary alignments only. To do so:
``` samtools view -@ 3 -b -F0x100 -o ${alignment_file.primary_only.bam}
${alignment_file.bam}```

### sort alignment files prior to quantifications and visualizations (IGV)
### for each .bam file (HCX16,20,21,25,26,30)

``` module load bioinfo/samtools-1.8```
```samtools sort /home/lbousquet/work/RNA/alignments/21_Aligned.out.bam -o 21_posSorted.bam```
```samtools sort -n /home/lbousquet/work/RNA/alignments/21_Aligned.out.bam -o 21_nameSorted.bam```

## Search for unannotated tRNA amongst ncRNA candidate sequences
### using tRNAscan-SE (v.1.3.1) and tRNAScan-SE-On line
```tRNAscan-SE -G -o tRNA.out -f tRNAstructure.out -m stat.out -M missed /home/lbousquet/work/RNA/snoRNA/seq_cand.fa```
### using Plant-tRNA database online
http://seve.ibmp.unistra.fr/plantrna/blast/

## Search for snoRNA (H/ACA et C/D type) amongst ncRNA candidate sequences using snoReport (V1.3)
```module load bioinfo/SnoReport-1.3```
```snoReport --CDonly < /home/lbousquet/work/RNA/snoRNA/seq_cand.fa > CD_unknown``````snoReport --HACAonly < /home/lbousquet/work/RNA/new_try/snoRNA/seq.fa > HA_unknown```
## Check for secondary structures
### RNA folding form (version 2.3 energies)
### UNAfold online server
#### default parametershttp://unafold.rna.albany.edu/?q=mfold/RNA-Folding-Form

## Identification of snoRNA and their targets
### Infernal
These results were generated using the *O.tauri* genome and RFAM V12 covariance models.
```cmsearch -E 0.001 --cpu ${NSLOTS} --tblout ${TMP_DIR}/${TMP_DIR}_${CM_BASE}_infernal.tbl ${INFERNAL_DB}/${CM} ${SEQ} >> ${TMP_DIR}_infernal.txt```

We filtered Infernal results using the following criteria:
0.8 minimum coverage
0.001 e-value threshold
In the case of overlapping features (coordinates and strand), we only kept the one with lowest e-value.

### Snoscan Server (V1.0) "Search for C/D box methylation guide snoRNA genes in a genomic sequence" Default parameters were used, with O.tauri rRNA and snRNA sequences used as targets.
http://lowelab.ucsc.edu/snoscan
```

snoGPS Server (V1.0) "Search for H/ACA snoRNA genes in a genomic sequence" Default parameters were used, with O.tauri rRNA and snRNA sequences used as targets. http://lowelab.ucsc.edu/snoGPS/

BLASTN against snOPY orthological database (http://snoopy.med.miyazaki-u.ac.jp/)http://snoopy.med.miyazaki-u.ac.jp/snorna_db.cgi?mode=blast_form (BLASTN 2.2.26)

Conservation of snoRNA sequences across algal genomeshttp://bioinformatics.psb.ugent.be/blast/moderated/?project=orcae_OsttaV2

Quantifications were performed with FeatureCount from Subread package (V1.6.0)

quantifications were run on the final list of candidates.```module load bioinfo/subread-1.6.0```

middle-size RNA (50-300 bp, paired-end, stranded)
```featureCounts -f -M -O -B -C -p -s1 --fracOverlap 0.8 --largestOverlap -a /home/lbousquet/work/RNA/osttaV2_cand_new.gtf -o /home/lbousquet/work/RNA/count_ostta_cand_new.tab /home/lbousquet/work/RNA/alignments/21_nameSorted.bam /home/lbousquet/work/RNA/alignments/25_nameSorted.bam /home/lbousquet/work/RNA/alignments/26_nameSorted.bam /home/lbousquet/work/RNA/alignments/30_nameSorted.bam ```

small RNA (19-50 bp, single-end)
```featureCounts -f -O -M -s 1 --minOverlap 19 --fracOverlap 0.8 -a /home/lbousquet/work/RNA/annotation_files/osttaV2_cand_new.gtf -o /home/lbousquet/work/RNA/count/small_80p_20190704.tab /work/lbousquet/RNA/reads_fastq/HCX_16_name_sorted.bam /work/lbousquet/RNA/reads_fastq/HCX_20_name_sorted.bam```

Supplementary File 2 : The O. tauri snRNA and snoRNA U3 sequences

>Ot-U1

ATACTTACCTGTCCGGGACGCCTGCCTGATCACGAAGGGGCAGCGTCGGGCGAGGTCGTCGC
GCCTTGCACTTCGCGCGTACGTAATCGCTTCGTTCGTCGCCTAACGGGGCGAACGACGGAAC
AATTTATTCCAGGGTGAGACCGGCGTGCGCGCCACTCGCCATTGCT

>Ot-U2

CGTCCGTACTCACACACGTTTTCGCAGATCAAGTGTAGTATCTGTTCTATAATCCATAATCG
GATTATTCTCATCATTGTATGAGATTAAGATTATGACAATTTCTTTTGGAGGTTCTCGTATT
CAAGCCGTGCTCGCACGGTCACGAGAACGAACATGGGCTTGGCCTTGCACTTCGCCTCGCAC
GTGTGCTCCATG

>Ot-U4

ATCTTTGCGCATGGGGTTGTTTCCTTGAGAGTGAAGTCTTGACTGACTCGGGAAGACTGCTG
GTTGAAAACTTATAATCCATCCCCAAGCGCGCGTTCTCGGACGCGCGCTTAATTTCTGGAGC
CTCGACGACGCGTCGGGGCCTTC

>Ot-U5

TCCCGAGGCGGACGTACGCGAAGCCTATACGCTGCCTTTTACCGCATATAGCTCTGCGTTCA
CCTCACTCAAAGGGACGTTCATCTAATTTTTCTCGAACGTGTTGTTCACAACAAAAACCCCT

>Ot-U6

GTATTTCCTTCGGGATTTACATTTGCAAAACTGGATAAAAATACAGAGAAGATTAGCATGGC
CCCTGCGCAAGGATGACACTCAACAAATCGTGAAAAGTCACATGTTTTT

>Ot-U3

AGGATCGTATTCTACATTTCGTACACCTAATCAGTTTTCTCCAAATGAAAACAAGCTTCTTA
CCTCAGACGACGACCGAGAAGGCGGCTCGCCCGAGCTCGACAATCCGCTTCGCGCTCGCGTC
GGCGACGCCACGCGTCGCCCGCGTCGCGCTAGCTTGATGAAGGCCCTGTGAGACACATTCCG
ATCACGGCGGACGGCGAGTCGTCAATTCTGATGAGT

Supplementary File 3: Ot- snoNA sequences and conserved structure elements

Capital letters correspond to reads sequences. Minor letters correspond to adjacent sequences in the genome, not included in the mature CD snoNAs. Consensus C, D, C' and D' boxes, and H and ACA elements in CD box and H/ACA box CD snoNAs are labelled in red. Terminal Inverted Repeats are underlined, including the few nucleotides present in precursor of CD snoRNAs corresponding to minor letters, absent in the mature C/D box snoRNA. Green and yellow highlight, indicate ASE in C/D or H/ACA box snoRNAs complementary to rRNA or snRNA targets. Blue highlight in Ot-CDsno13, the homolog of U14, indicates the rRNA complementary sequence of this conserved snoRNA which is implicated in pre-rRNA processing in all eukaryotes.

>Ot-HAsno1
ACACTGCCACTGTTAATCCCTTCACTGGACTTTCAATGTCCTTGGAGTTTCAAATGTGGCAAACATCA
ACTCGGGAGTTTCGCTGACAGCACTCGCACGTTTCGGCGGGCAATGAGTGTCATCTGATAGTAGCTC
TCGCACATTT

>Ot-CDsno2
TGACGGATGATGGCGTTGAAACGCGTCGAGCGCGTGACGAGAGGGCGATGTACTGTTGAGAATCAA
CCATTCGCGTGGTGCGTGATCTCGAGACTGACGTCG

>Ot-CDsno3
TGACAGATGATGAATTATGATCACGCAACACTCACTGATTTCCGTGAGGATCCACAATCATATACGAG
CTTTTTAACACTGATGTCAC

>Ot-HAsno4
TCCCCGTCGTTCACGTTCTCGAGATCCCAATTCATGGACTGCGAGGATGGGCGTTTTCACGCCAACG
ACGGCACAACCATCCGGGAAAACAATACCTATTCCAACTCTGATTTCTGAGGTTTTGGAAGATGCTTT
TTCTACATTT

>Ot-HAsno5
TTCCCGGCCGCTAGTTCGTTAGCCGTCTTCCCTACTCCATGGGTCTGGGGGCTCGGAAAGCGGCCG
AACATAAACGTGGTACTCTTTCACGTGCGCGTCGCCGAAAGCTGGCGTAGAACCCGGTAACCAGGA
CGCGCACAGATTGAGGCCCGAAATTT

>Ot-CDsno6
AGGTGTGATGATGCTTCGTTGTACCTGGTAAGTTTCCGAGCTTCGGTACTGAGAGAAAATATTTTCAC
AACCCTGAACACC

>Ot-CDsno7
gATCCGATGACTATACATAAATATATGCAGTAATGTGAGACTCGTCGCACTGCTGTTATTGCCCCTGA
CTTTCAGTCGGACGTCGGCGGTATCGCAACGGCGTGTCGTTCAACATGAACAGTGAAGGCAACTATG
AGGATC

>Ot-CDsno8
AAGCGATGACGACAAACAAGAACTTACTACTTCACTGATATCCGTGATTATTGTTTTTGCAAATGTCTT
TCGCTCCTATCGTGAGCTTC

>Ot-CDsno9
AAGTCGCTGATGAACAATTACTCGATTCCCGACACTCGTGCGTGAGGGGACAATCAATCACTCTAATT
TTTTACTGAGACtt

>Ot-CDsno10

TGCGGATGACGATCAAACTGTTATCCCTGTCTTACGCACCAAGGCCGGTGTTAGTTTGATGATCAAGT
TATTAGCTCTAACCGACGCA

>Ot-CDsno11
TTGCCGATGAGGAACCAATCTTATCTTAGCGGATTTCTCTGATTCTTCGTGAGGAAACTCAAGACACT
GTTACACATCACCAAGGCAACGTTGCTCGGCTCGAAGACGTGTCAGGAACAAACGTG

>Ot-CDsno12
aaaCGTGAACGATGACAATTTACATTTTCGGATTCCCTCAGAGCATGAGCAGTGATCGAACATGTACC
AACCTGACACGTttt

>Ot-CDsno13
CTCCGAGGATGAAGACAGCAAGGGCGTTTCTCAAAGAAACCATTCGCAGTGCCCGAACTTAAGAGTT
TTCGCTACCGATTCGTCGGATGCCTAGCTTGAGAACTTGTGCAGGCCCCTCCTTCCTTGGATGTCTG
AGGAG

>Ot-CDsno14
GCGCAGAAGACGACAACCGAACAACAAATGCACAAGTTCCATCTGACTTCGGTGAAGAAACGAACAC
GTTTACCGATGCGC

>Ot-CDsno15
GATGTTGATGATGATTTTGACATCCCCGGAACTATCGTGGACACCACCACGGGTGAGTGCCAAAAGG
CTCAATGGGTCCGAGGCACGAAGAAAGGATCGTTCGACGCGTACGCGTTCGCGACGTATCTGAGAG
ATCATC

>Ot-CDsno16
GCGGTGGTGATGATATGCACACAGCATTTGGCCTGACTACTCACTGACGGTTTTTCAGAACATCTAA
GGGACTGAGCCG

>Ot-HAsno17
CGAATGCCAGGTCACCTACGACGTCTCTTTCAATGAGACGCTCGAGGCCTTCACTCAGTGGAGACTG
GCAGACAACACACAGACCAAATCTTGGGCAGGGTGCCGCGCGTCTCCCGTCGGCGAACATCCAATC
AAGAGCTGGACATT

>Ot-HAsno18
ACTTGCATCATCATATCGAAGACTCGGGTGTTGTCCTTTCCATGATCGTCCGGGGTCAAAATTGATGC
TACATCAATACCGTTCGCATCGTAATGCTTGTCGGCGTCGATATCGCCGTCGGGCACGCGAAGCGAC
CGGGACACAC

>Ot-CDsno19
ACGTCGAGGACGAGTTCATTGACGCTTGGCTCCTGAGACGGTCGCGCGGCCGGGGTAGTTGCCGG
GCACACCCGCGCGATGCGTCGTGATTAGAGATAAGCTGTTCAGACCGAGA

>Ot-HAsno20
AAAGGGCGTCTGTGTGTACGGATCGCGGGGCGGCGCGAGCCGCTATCCCGCGAGCCCGAAGGGAT
TTCGCGCCCGATAGTGAAGGGATCGATCATTGTCACGCGCGCGAGCGGGAACGTGACTCGCCGTGC
GCCCTTATTCGGTCCCACATTT

>Ot-CDsno21
GTTCGGGGATGTAAACGTGTCCACATGGCTGATCACCGTGAGGAATCAAAACATAATATTCACCATCT
TTCGACTGAGACGA

>Ot-HAsno22
GAAAGCCTTCGCTGCAGAGCTGCGCGCCGAGAGTTGGGGACGTAGAACACCCCCGATTCGATTCGT
TGTGTCAGTTTTGAGCGGAGGCGAAAATAAAGGGGATGGGCTCTCCGTGGAGCCTTGGCGTTTCTAT
AAGTCGTCGAGGTTCCTCCGTAGTTCCACCACAAC

>Ot-HAsno23
TTTCCTTGACAAAGCAGAGGGCTGATCAGGCTTAGGCCGCATGTGCTGCTCTCCCGTCGTTCTAAGG
TACAGCCGATAGCGTCGGCATGGGTGAGTAGGGGGCTGCCTTTCCACAGTTCTCTGCTCAATCGTC
GTCGCGACATTT

>Ot-CDsno24
TTGGCGGTGATTCCAAAAATCTGATGCTGATTATCTATGAGGCTTAATCAAACCTCTAAGAAGTAATGA
AGCCAACATCGTGCGACGAAGTGGATGAATGAGTGGACGACATCATTCAGCGACGAAGGCAAAGTC
AATGACGAATATCTCTTGACTTAAGACGTCGTGAATGATCACCAAATTATATTGCTTCAAACTTCCCGA
GGCTT

>Ot-HAsno25
TTTTGCGACTTTGCCTAAGGCGCTCCTCACTTAACGGTGGAGATGATCTTTTTACTTCCGTCGCAAAC
ATCAATCTTTTTCGGTCCGATGACTCCACGACGACCGAGCCACTTCATCGGCTCGTCCGTCACTACAT
TAAGGACTACATCT

>Ot-HAsno26
ATTGCGCTCTACCGATACGCCGTAACCGTTTCTCTCGAGAATTTGTTGGTTGCGGCTCAACTGCGAG
CGCATATAAAAACGTGAATGCTTTGAACGGGCGACCAGCCCACCTTGGTTTCGCAGCCGAGTACTTC
TGGACCCGCCCTTTGCACGCATTGTAAATTC

>Ot-HAsno27
AAAAGCCAGGTCCTTAAGAACGCTCTCCGTCGCATCGCTTCCGTGATGTATGGAGAGAGATCGAGTC
ATAACTTGGCAACATTAAAACGCATCGCGACACGTATTGTGTCGCTTCGTGCGTTAGCGTAATCGCTC
GTTTTCGTGAAGTGTGGGAGATTCGTCGAGCTGACCTGCTTCACTCAGAGCGTGCCCGCGGACATTT
T

>Ot-CDsno28
gaGCCGATGATGCACACACAGCTACTAGAACCGAATTTCGCGAGCCGAAGAGGTCGTCTTCGCGACG
CCTCGACGGACGCTTGAGGCCAATCTGTTCGATATAGATGAGGCTC

>Ot-HAsno29
AACTCGCGAGTTTACCCCCGCCCTGGCGCTCACATCGCCTCCGGCGGTCCTCAACACCTCGCTACA
ATAAAGATGGACTTTTTAGGACACCTCCGAGAAACACTTCGGAGCCTTTCCCCGTTAAACACCGGGT
CGGCGACGTCTCACGCCTGCGCGTTGTGCCAAGGGGCTACAATTCAC

>Ot-CDsno30
CGCCGGTGATGACGAAATCACGCACCCAGCCAATCCGAGGACGTTTCGGCGTTCGCTGGAGACAAG
CGTACACAGTTACCGTTTGGTATATCGACTGAGGCGTTGGATACGGATCGCGATCGCGATCGAGACG
TGAATGAGGACTGACGCAACACTTA

>Ot-CDsno31
GGGAGGTGATGACAGAATACAGATACTTCTGAGCGCGCGCAACGCGTGTCGACATCGTTGGCCCAG
CGACGGCCGCTCCGTGAGCGAGTATGGACATAAGTTTTCAATCTGATCCCA

>Ot-HAsno32
TCTCCCGGCGTATCTATATTACCGTCTCACCCGTGGCGGTCGTACTCATGATCCGGGGTACATCGAA
CAGCTCGTCGGCTTGCGACGAGCGCGCGGCGCGAGCCACGACGCGCGCTCTACTTTTTGCCACGA
ACATTT

>Ot-HAsno33
GTACGTCGACGCGCACGACCTTCCGAGCGACGCTTCGCGTGGGGTGTGGGTTTAGAATAGGGTTTT
AGAAGACGCGAACGCGAGGCCTCGCGCCGACGTACGTCGATGACGAGAGAACGAAAGTACTGACTG
TGAACGCGCGTTTTATTTTACGAACA

>Ot-CDsno34
CGCCAGTGACGATCATCCATAAGGATCAACCAACCGAACATCCCTGTGTGGATTTTTTGCTAGTACTG
TCTGAGGCG

>Ot-HAsno35
AAAGCGCCTCTTTAGAGACTCGACGAGCCCCTCACTCGTTTTGTTTTATGCTGGGGCGCGAAATAAA
CGCGCGATCGCGATTGGATGGTGACCTCTTTCGCGGTCTCTCTTCCGCTCGAGGTCGCTACAATTT

>Ot-CDsno36
ATCGTCGATGACGAACAAAACTTAAGGGATTTATGAGATTGGATTCATTCCATGTTCGAACCAAAGAC
TACAATTCTCCGAGACGAT

>Ot-CDsno37
GTTCCAGATGATTGCTAACATTACCTACATTCCCTGAGAGAAGACGGTGATTAAAACTCTTATCGATAA
CCGTTGATGGAAc

>Ot-CDsno38
tGGCGATGAAGATACCACTCTTATCACATGTTAGACTGTCGAGATCGAAGGGTGAGGATCCTTCGATC
GATGTGCACAACCAATGAGCGAG

>Ot-CDsno39
ACACAGTGATTATTAACTCTCTACGGATCCTTCCTTGGTGAATGCCGAGAGTCTATGAAGATTTCTTGT
TCGCTATTTTTGTATGAGTGT

>Ot-CDsno40
GCGGTGATGTGACCTTTTAGGTCTCGTTCGATTGAGCTCCAGCGCGTCACGACGGGTTGGTGGCCT
GAAAAGAATAACGGAAACTGAGCCATCTGAGCTGTGT

>Ot-CDsno41
cgGTGGATGGATGAATTGGACGCCGACGGGGGCGCGCGAGCGCGTTCACGAGACGACGACGCGCG
AAAAATACGCCTCGACGCGTGTTCACTGACGa

>Ot-CDsno42
tTGCGATGATGACGAAAATATTAGATGACGAATCTGACAGCTTGCGCCATCGCGGTCTTCGCGACTGC
AAGCGCCGTTTCGACGGCACATGATCCGACAAACGAGGCATTTGTCTGCAGCAA

>Ot-CDsno43
ACCCTCTCACAGTGTCAGAGTGTCAAGATTCTTCGTGATTGCCACCTTGGGTGACGAGGGAAATTGA
ACGTCTCGGTACGGCGTTACCGCGCTCGTCTTCACGACGGCGTCGGGGCGACGGGCGAGTGACAT
TGT

>Ot-CDsno44
CGCGCGTCGAGATGAGACCGTACCGCAAACACGCCAGTTCTGCTTCTGATTGAACGGTGAAGAATG
GTATGGATCTGAACGCGCG

>Ot-CDsno45
TGCGTCGTTGATGGTTGAGTGATCGCGCTGAACGTTGCGCGAGGGATTGGGATCGAAGCGATGGGC
GACTGACGGTGTCCGCGACGGGCGCA

>Ot-CDsno46
GTCGTCAAGGATTATACACCTTCAAAAACACCATCTTTCGGATCTGAAAGTCATTGAAGAGAAACGAA
TACCATTGAATGAGACGACGGTAGCTTTGTGGCGAGAGTGAAGACATGTAGAACGAGCGAACTGACC
ACGCC

>Ot-CDsno47

CGCACGCGCGTGGAGTGGGAGAATTCGATCGCGATCGTCGGGCGGCGGCGCGAATTCAATGCACT
CGAAGAAAACGAAACGCGCGCGACGCCTTGGTGTGACACACGGAGACTGACAGCGTGGA

>Ot-CDsno48
AAAGGAATGATGATTCTCAATTAGATGTTTGACGACGCACTGTTGACGGTCATCGTCGGCGCGCGTC
GCTGATACCACAAAATTGAGGCATATCCATTCTTAAACTGACCTTTGAGTGGCTGACTTG

>Ot-CDsno49
ttCGGCGATGACGAATCGAATATCAACCAGGTAGCGGAACATCCCTATGTCGAGAAACATATCGCTGC
GTTCTTACTGAGCCGAAGA

>Ot-CDsno50
TGGAGGTGATAGACAACAACAAAGTTCCAGAATTTTTCGCTCTCTGGCGCCACCGTTTAGGCGGGGT
TATAAGTCAGAGAGTCCTATGTTGGTAACTAAGCTCAACAGGCCTGATCCAT

>Ot-CDsno51
tGGATGTGATGGAGAGAACGTCGCGCCGCGCGACGAATCGCAGCGAACGCGCATCGACCGAGCTGA
CCCT

>Ot-CDsno52
GCGACGGTGATGACGAAAATATCCGCGCTTCTGATGAGCCTCGGCTCGTGAGGTTAACGATATATTA
TGGTTGAATTTCTTCGGCTGAGTCGCTATGACGTGGTTCCCATCGTCTGAGCG

>Ot-CDsno53
ccatcGTGCTGATGAAAACCTTTATCAAGCATTTCTCGTTCTGAGCAATATATTCTGATGAACATCTATCA
CCCATAGAATCTGAACGAtgg

>Ot-HAsno54
aaagCGCGTGGGACGGGTTCATTGATCGAGGCCGGCTTGATACCCCGGCTGTGCTCTGAGCCATCAA
TTTTTTAAACGCGCGACATTAACACCTGGGAATATATCGGGGTTCCCGCCGTATCAGTCGATGTCGG
AACCGTGAGCGATCCTTGGGACATTTtttt

>Ot-CDsno55
GGGGGTGATGAGAGCGCTCTTTTTGCTAATCTTCTCGCCGAGCGCGTCCGCGGTCGACCGCGGTGG
TGGACCGGGAGACGGGCGCTGAGGAGGATCGAGGGAAAAATACATGTCATCTTTGCTCTGACCTC

>Ot-HAsno56
CGCACCGACTTTCTTACCTGTGACTTCGCTCTTTTTGTTGTGAGCATTGTTGCCTTATAAAGGGTCGG
AGATTAATTTACAGGGCGCCTTTACCAATATCTGGTCGAAACCTTCGGGTCATGGCTAAGTTATAATG
GCTTGGTGTCCACATTCA

>Ot-CDsno57
CGTCGTGTCGACGGATGATGATATTTGTCGCGTCGCGCGCGCGTTGAGAGAGCGAGATCGGCGTCG
CGCGCGAAGCGAGAGAGGAGTCAACTGACGAGAATCGCGCGTTTTGCACACCGCCGCA

>Ot-HAsno58
CAACGCATCTTTCAAAGCTCCGAGGTTAATCTCACGAAATGCCCCTTGGAGACTACAAGAATGCTACA
GAGAAAGGCCTGCGCACTAATACTCGCGGGAGTTTATTCACTCACGTGGGTTACGCTTCGTCGGTAC
ATTC

>Ot-HAsno59
AAAAGCAAGCTACACTCTGTCGTGAGACCTCTTCATGTCACATTGCAATTTTTAGGCTTGCGACATCA
AACAGCGGATCGGATTTGCCGGCGGGGCAGGTGTTTTTAGTCACGGTCTCTCTCTTCCTACCATTTG
ATCCACATTC

>Ot-CDsno60

TCTGTCCTTGATGAATGCGCGAAAACAATAGTACTGAAAGCCTCAGAGCCTTAGGGCTTTGATAGAGT
AAACCGCTTCCTGAGAC

>Ot-CDsno61
GCAAAGCTCCATGACGATTCTTTCACCTTAAGGCGCTGACATCTATGAGGCATCCTTAACCTCCTACT
CATTCCGAGAGCTTTGC

>Ot-CDsno62
GGGCGATGAAGAAAACCAGGCTCCCTCTACTGATATCGGTGAGGACAACGGTCGGCTGAATCGAAC
AATTAACTACCCTGGCCAG

>Ot-HAsno63
AAGGCTGGTGGTATCGTTGCCTGCGCTTGAGCTCCGCGATGGGGCGCATGGCTTGTTCCCCAGCAA
CATCAAAAGAGACTTGGTGACAGGTGCTCATGCTTACCAGCGAGCATACGATCACTAGGACTCGACA
CGCG

>Ot-CDsno64
ACGAGTGATGACGATATACCATACAGTGATTCACACTTCAGTCTGAACACTCTTGTGGACTACTTTTC
AGCCTTGTCTGACGCG

>Ot-HAsno65
CACGGGAGCTAATCGTGAAGCCGGCCCGCGAGAGATGTCGAGGCGTCGGTGATACTACCTCCCAAC
AACAAAACCGAGCGACAATAGAACCGACGATTCTGAGCCATGACCGTCGGTCTACACATCGTTCCAC
ATACA

>Ot-HAsno66
TGCGGGACGAACGACGCGCGCGAGGGCGACGATTGGACGACCCTAAACCTAAAACCTGGATAGGG
CCCGCGCGCGGAGGACAGTGAAAAAGCGATCGCGCACGGTGGACACCCGGATGAAAATCGAGAGA
CTGCGAGAACGCGACGACTGACGTGTGATCGCGCGATATTG

>Ot-CDsno67
TACCGGTGAAGAAGCAAATAAGTTCCAGCTATCGCTGAATATTCCGTGATTACGAAACTTACGCACTC
AAACCCCTGAGGTTTACGCGTCGCAACGGGTCGTGCGAGATGAGACGCTCGAGAGAAGCGAGAAGA
CTGACGACGCGGTTGCGCGCT

>Ot-CDsno68
CAAGTTGAAGAGCGCATCGTCGGCGCCCGCACCGCGACGGAGAGCACTCGAAGCACTCGAACCAT
GATGACACAGATATCACTCACGCTATCGGACGATCTCGCCGCAGAGTCGTCGGAACAGTCGAGTCC
CCTCGCTGATCTCACGAGTGCAATCTCTCAACCGACGCGCTCAAACCGTCAGTG

>Ot-CDsno69
GGTCGGGAGGCCGGCTCCACGGTTTTATCCAATATGATGAGTCGGTTAAGTCGATGATGATCATCGA
GACGTTAGCCCCCGCCAAATACCTTGTTACTTTGCGAGGCGTCAACCAGCTCTGACCCCcttttt

>Ot-CDsno70
GTCGGTGATGAATCCTAATTAATCTTTGAGAACTGATCATCCCCGTGATGTCTCAATGACAAGCATAT
GACTGAGACCACTGCGAGCGGAGACGACGACGGATGGACCGAGTAAATGTGTGGACGGGAAGAGA
GGACGAGAACTGACGCGCGCGATACCTTTTGTGCTCACGACGCAG

>Ot-CDsno71
TGCGTCGATGATCGATCGTTTCGACGCGACGTCGGCGTCGCGCGCGCGTACGGGGGGATAATTCGT
CTCGGACGCGCATCGGCACGCCAACCCTAAGTACTCTGGCTGGAGAAGAGTTGACTGACGCGCCGA
CGCTTCGTCGCGATCGATGAACGCGCag

>Ot-CDsno72

GGGGTTGATGATTATGATACACCAATCACAGACCTGATCTGAATGACCTTGTTGAAACAATTCATGCA
CCACTCTGACTCACGTCT

>Ot-CDsno73
TGTTTCTATGATGAAAGATTCTCTTGTGTCAGGATTCTGATTCCATTGATGAGACATACGAATCTGCTG
CTTTCCTTCTGAGAAACAAGAAGACA

>Ot-HAsno74
CTCCACGAGGATTGGAGCGTCCGACCACGATCGTGGCGAGCACTCGTGCGAAACGCGAAACGGGG
TGGATATCCAATCGTGGTGGTACATTCGATTGATGCCAGCATTGTCGGCGTTCGTGTCTTTTCGCTCG
CAACGTACTGCGCTTCGCAGTGGCTCGAACCGCCTAGAGTGTCGGTGCACATTC

>Ot-CDsno75
GATTCCGGTGACGACAAACCTAAAAATACACCATAGATCTGTTCCGAAAAATCTTGACGACAAAAACA
TGCACCACTAACTGAGGAAT

>Ot-CDsno76
CCGGCATATGAGGATAAACAACAGATAGGGACATCTTACGCGCGAGAGTGATGCGAAATCTGGTCAA
CTCGATGGAGACATCGACGTTCTGCCGATGCCG

>Ot-CDsno77
gaCGGCTATGATTAATGTGTTTTTAACTCTCTTTCTTACCTGGACCGTGGGGAGACACAAATCACCTC
TGACAATATGAGCCGTc

>Ot-CDsno78
AGTGCACTTGACGACATAGGATGAGGCAACGGGCGCGTTCGTGACAAGAGACGACCAAGCGAGTAA
TGACTGACATTACATTTTCAACCATCGCAGAG

>Ot-CDsno79
TGCGTGCGATGCGCGCGCGGTGATGGGCATCGCGGGGGAATGATTATGATGATTGGCCACGGGCG
AATGATTGGAACGCGTGGGACGGACGCGCGACGCGCGCGCGATGCGAACGGGATGACTGACGAAA
CGAATGGTGTGGTGTGGTGACGCAG

>Ot-CDsno80
TCACGAGGTGATGAGCCCGCGGCTCGAGTTGCACCTCGGACCCTCGGACCCGGCGCGCAAGCGCC
TCGAAAACGGGTCACGATCATTCCCCTCGGACGATCTCTGGCTCTGATCGC

>Ot-HAsno81
TCGGTGCTCTATAATCCTAACGCACGAGGCGAAGAGCCACGTCTCGATGCGTCTTGAAGAGCACCTA
CATCGACCGCGGGATTAGGCCCCCGCACCGCGAGAACGCGGGGTGATCGCGTGCGATCGCTTCGG
CGTCGGCTTCGACGCTCGACGCACGTTTGAGCGAGGGAGACGACGACGGCGGGACATCT

>Ot-CDsno82
TCGAGAGTGATGATAACTAATCGAGCCTAAAGCCCGTGATACATTCGTGTCATCACTTTGCGCTGCTG
TGGGCATTATGTCATAGTTACACTGACTCGA

>Ot-HAsno83
TCGCGCCATCGACCTCACCGGAGCATGTGCTCTGTGCCATGTTCCTGATGTCTTTGGCGCTACAACG
ACGGGCCATCTTCCATCTTGCCGCTTCCTCACGTGATGCGTCGAGCCATAATTGGTCTACATTt

>Ot-HAsno84
TTCGCCGCCGCCAGACGCTTCCTCCGAGACGTGCCGGTCGTCTCCGAGGTGTCTGGTGGTGGCGTA
CAGCCAACTCGTCGATCTGTTGAGTCTCTCCCATGGTACAAGTCTTTGGGAGAATAATTTATGTTCGG
CACATCT

>Ot-CDsno85

TTCCGATGACGATCAATGCACATCAATCATTCATGCGTGTCAGCTGAGTTTTGCTGTGATTATGAATCT
AAATCCGAGGAAA

>Ot-CDsno86
ACGCGGGTGATGCGCGATATACAAAACGCAATGATAGGACTGATTCGCGTGAGGACATCCTTTAATG
TGCTGACGCGT

>Ot-CDsno87
TCGGCGATGAAGATTCACACATTCTGACACCTCTTGTACGAGACTTGGTGGCCCTTGTTCGATGAACT
CATTATGCAAAAGACTGAGCCGA

>Ot-CDsno88
GAGGGATGATGTCGAGAGTTGTCGAACTGAAAGCGGCGTCGAGGGATGACGCCGTGTGGACGCGC
GCGACCGCGCTCGAGTCTCGCGACCGATGAGAAACGATGAATTGAATATTTGTACTGACGTTC

>Ot-CDsno89
GCTCGCGGTGATGATCTACCTTTACAAAGCTGGAATTACCAACTGAGGCTAGAATGATTTGGGGCGA
TGGAGCCCGGCCCTCTTACGCAATGCAGAGTATAGGCATCTGAGCGAT

>Ot-CDsno90
tgGCGCGATGATGACTTTCCATATATTCAATCTGTCAATCCCCTGATTTTCAGTGATGACAAATCATATG
GTGCCCTTCCACTGAGCGT

>Ot-CDsno91
tCGTCGCGTTGATGATAAGCCGAATACGCGAACGCTCGGCTTACCGCCGGGCGCCTGTGACGATCTA
AAATTACAGAACATCTAAGGCTGAGCGACG

>Ot-CDsno92
TTCCGATGATGTCTTCAAAGCTTAATCTTTGCTGACCATCCTCATGAAGATGACTAATGACAAAACGAC
TGAGGAA

>Ot-CDsno93
CGTCCGATGACGAGCGCACATCATACTTTCGTTCTTCCGAAGAGCCCATGCGCTTTGTGGAACGCGT
GATCACCTTGGAGAACCGAGGACG

>Ot-CDsno94
ctCCTGTTGATGAGAGACCTTTGTTTGGAGACCTGATCGGACACGAATTCGTGGTGTCGAGAAAACAT
TGGAGACCTGAACTGAGGGAg

>Ot-CDsno95
TTGCGATGATGAGAAAACGCTATCTAGATACTCTTTACGATGAATGTTTCTGGGTTTCGGCCCGCAAT
GTTGATTACTTTACAGCAATGTGGTTTCTCTGAGCA

>Ot-CDsno96
CGGCGATGATGTCATTAATTCATGCTAATCCTGATCAACCGTGATATACGATCGATAGGACTGAGCCG

>Ot-CDsno97
GAAGCTGTGATGATCGCGCTTTGTTTTCGCATAGCGTGGAGAGTTTCGACGATGAATCCCGTAAATA
CCAATGGATCCTTCTGAGCTTC

>Ot-CDsno98
GCGTGTGCGAGCGCGCGCGGCGCGGACGTTGACGCGCGACGGCGACGATGATGATCTCGAAGCGA
CGCGCGCGTGAACGGTTCGACGCGTGACTGACGACGACTGGACGCGCGACGCAG

>Ot-CDsno99
CATCTCGATGATGACAACTGTGACGGGCGGACTGCACGGGGTGATATCCGGGTGAAGATCAGTCCT
AGCGTTAACTCACGAGTGCGCTCCTGAGAGATG

>Ot-HAsno100
TGACCACGACAAGTCAGCTGCCGAGCGTTCACATCTCACCGCTCGGCGCGTTACGCTCGTGGAACA
TTAAATGCTCGAGTCGCAAAATCAACGCTTCGTCGCCGTTTTGGCGATCGTGAGCGTCGATCTCGGG
CGCACGCACCGCCTTGAGGGGCGTCGGGGACGTTTAGAAGGCACTCGTGCCTCGCGCCGCGACGG
GGACCTCAAGGGGTGGGACATTTA

>Ot-CDsno101
CGCGAGTGATGAGAAATTGTCACCTGATAGCATCGCGCCTCCGTCTGAATGGACGGATTCATGCCTA
AAAACCGTGAGGAGACTTATTTCTGACGCAT

>Ot-CDsno102
TCGCGGTGATGACTGGAACTTTCTACGGACTAGAGTTTCTGAAGATTCGAGTCTCGGCTCCGGTGTT
TACACAACGTTTCCCTTGGCTGTCTGAGCGGA

>Ot-CDsno103
GTCGAGGAAGATCAAACCTATACCCAAAAACTTTACCGAATACCCAATGTGGATATTTTGTGACCATA
CTCTCTGAGACAAAAGGAGAGACAACGAGCGTTACGACTGACGACTTTTTTTCTGATCACTTCATCGC
AGT

>Ot-HAsno104
TGGGCGTCACGATTTACGAGGCCGAGCGATCACCATCGTCGGTCGCGTTTCAAAACGCCCATACATT
AACGCTTGGGTAAAGCGGGCTTCGTGCGCTTCCATCGCTAACGGCCTCTAGAACCCCTGCTACACGT
G

>Ot-CDsno105
gtgGATGATGACTTCGTTAAAAATATCTTAGGACACCTTTGACGCGTTTCGGCGCGGTGAAAAAGATTT
ACGAAGCACTGACGCGACGGACACTGATAt

>Ot-CDsno106
CGCGCGTGATGTTTGAATCAAGGCATAGTTCACTTGAGGACTCGGACGTTCAGCCGAGTCCCTTGAA
ACGAGCATCGATTCACTGACGCGATCGGGGAAGGGATCTCTCTCGCGGTGTTTGTC

>Ot-CDsno107
gaGCCGATGACGACAAGCACTTGTCCGTACCATCCGATCGAACGGTGACGAACAAACTTGTGAATCT
CGTTAAACCGAGGCTT

>Ot-HAsno108
CACGGCGGACCGTACACTAGAGTCACCGCCCCGCGCGAAGACGCGATCGCGGGTGCGGCGCTCAG
TCCAGCCGACATTGAACGCACGTGTCTTTGTCTCTCGCGTGTGTTTCGGCGAATCGCGAGGGATGGA
GGACGCGGCGAAGCGCACGGTAAGCGAGCGGACGCGTTCCGCCACGCCTGGGCGCGAGCCGGAC
AGTT

>Ot-MRP
CATCGAGCGAGACTCGCGAAAGAAAGGGTCCGCGCGCGCGGCGGCGACGAATGTTAAAAAATGGAA
CACCGGGCGAAAATCCCCGGGCGCTGAGGCAGAGAGTGCCGAGTCTTGACGACCAGGACCTCGGA
CGCGGAGCGTTGATTTACGATCGACGAATTCCCGCCGAGCACCTCGCCGCGGCACAGTCATGTGTC
CACCGCGGATGCAACGCGGTAACTCAACGGGGTTTACTCAGAGCGCTGAC

>Ot-CDsno110
AGCCGGTGAGGATCACTGCTCACAGTTTGTTTGAAGGGAGGTCAGAGCCCCTGCTGAAACGAATCTG
TCTAGTACAATGCCGTTTACCCATCTGAGGCTTC

>Ot-CDsno111
gcGCGGATGACGAGTGAAACGACCGCGCAAGGGCTCCGACGCTCGACGGTTCGTCGAGCGGTGAAG
AGAGACGTCGAACACTAATCTTCTCAAAATATCTGACGCGt

>Ot-CDsno112
TGGTCTGTGATGATTGAACCATTGGCTGAATTCTGTGACGATTTGTTCTGTCTGCCGACTTCCCACTG
AGACCA

>Ot-CDsno113
ACGCCGGTGATGTTCAATACTGCGTTATCTTCCTTATTAGAGCACTGCGACGGGTCTCTCGGGACTC
GGGCGCGGAGCATTCTGTGAGGCAAACCAACTGAACCTGAGGCGT

>Ot-CDsno114
CTCGCGGTGGATGACAAAAACTCAAGGCTAAGGGGATTCCAAACGTGACCCATACCAAGCCTCCGCT
TAGTACCTGACGCGAG

>Ot-CDsno115
AGAGCCGAGGATGTTCAAATATATTTGCTACTCTCGAGGTTCGACGGTGACGACACTCAATCACCAA
GATCGCTGAGGCTTT

>Ot-CDsno116
TGGGTGGGGATGAAATTTTTTTCAACTGGTCTTCTTTCCACTGAGGCGGTCGCTCTCGGGCGGCCGG
TGAGGAAAACATCCGCAAATTGAACTTTTCTGAACCCA

>Ot-CDsno117
tcaaACTTGATGATGAATCCGGGTGGAAGAGCCGTATGATCCGCATGCTTAGAAACCGACCCTACTTAA
AGTTTGAACGGCGAAGAATGAGAGTCGAAGTTTCTTTGATCCGGGACTGACCGAAta

>Ot-HAsno118
TCGGGCTCCTCTGCCACGGTTTCCTGAGCTTTGCGCTGACGGTTAACTGTCCAAAGAGAGCCCTACA
TAGATTTTCGCCTTCATGATAGGAAGCCGTACTGTCGTCTTCGGGCGTGACTTTGCGGTGCCAACCG
GGCGATTACATTCAC

>Ot-HAsno119
TACGCGTGGGGCCTTCACCCCGGGCGGTGCGGCGTAGACTATCGCCGGACGCGTCCCAAGCCCCG
CGCAACAACCGAAGCGTGCGCCGTGTTGCGCGCCGGGTTCACCGTGCTCGGTCGCGTGTCTTAAAG
TTGCACCGACATTT

>Ot-HAsno120
CGTCGCGTCGAGGCTCACTCGCGTCGGCGGGACGATGGACGTTCGTCTCGTCGCGGGGCTTTGAC
GCGCACATTTGGTTACGCCAGATATAAACCAATGGCGGGTTAACTGAGTACCCGTCGTTGACCTGTC
TCGCTGGTACATTTT

>Ot-CDsno121
AGCGGCGTGACGACACGCACTGCCGCCCCAGTCAGACGACGTTTCGGCGTCCGTGGTGATTGGTTA
CGCATGAATGTCGCGCTCAGGCGCGATCGGATCGCTC

>Ot-CDsno122
CGCTCGCGAGGAAGATCGAACGCTATTAACCCAGCTCGCTTTGAGCCTCGGCCTTGAAGAAAACTTG
TTCCAAACTACACGACTTTTGTCTGAGCGAC

>Ot-CDsno123
TCGGAAATGAGGACTTCTCTTTCCCCAATTGATCGAAGATGAAAAGCATATAGAAGGCCGGTGCCAC
CGGCTATACTTGCCAGTATTATCTGATCCGT

>Ot-CDsno124

GGAGATGAGGAAGTTGATGGTCCGTGTTTTCGATCGACCCAACTGTCATGAGGCGAAAAACATCGGG
TGTGACAACCCGCTTCTGATCTG

>Ot-HAsno125
TTTGCTTCGTTAAGTTGGGCATTTTACTCCGTCAGTGTGCTCTTGCCCCGTTCCCTTGAAGCTACATC
GATAGGTCGGCCCGTTGAATCGAAGCTTCGGGTTCGGTCATGCAACTGGTCTCTCGCTATGTTTGCT
CGCCGCTCACCGCGCGTGAATACACAGAGGCCAGACATCT

>Ot-HAsno126
GTTTGCGGGCTGTTACTTCAGGCAGCCGAGGGCGACATTCCGTTCGTCTTCGGCGTTAATCAACCCG
TACAGTAACGAGCTCGCATTCTGAGCGACCGCGGGAATGGGACTTACTACCCTGTGCCCCATGCAGT
TTGCTCTGGAAACCGGGCTTATACTT

>Ot-CDsno127
TTGCGATGATTAAAAATCTCGAGGCTGTTTGTTAGTAGGTTTCGGCCTGTGCGCTAAACACGATAACA
AAAACGACTTCTCCTTTCTCTGAGCATT

>Ot-CDsno128
GAGCCGATGATGCAGCTTTTTTCTGTGCGGTGCAGTGCGTCGTCGGGGAATCTGGATTTCCAGACGA
ACCTTCCCCGCGAGCGGCGCATGAGAATGCGTTCTCGGCTTGGCACAAACCACGCGGTTACGCCGC
TGTCTGAGGCTT

>Ot-CDsno129
aaGGAAATGACGAAATACCATTGTTTCACGACGGTCTTCTGATGCTTCGGTTTTGATGACCTGTATGG
TGCTAGTACTCCGATCCTT

>Ot-CDsno130
GACAAATGATGAGACTTATGGATTCCCCTTCAAGACGCCCCAAAGGCGCTGTTGATAAATCCGATAAC
AGTCCCTGATGTT

>Ot-CDsno131
gatCCGGATGATGTAAAAACACACGCTGAGAACGTGCGTGGGGTGTGGCGGTAAAATGTCACGCTTG
GCGCTCGAGTGGCTGTGCCGTTCGAAATGAGGGATGGTGAACCGCTCAAATCGATTTTGACGGACA
CATGCGCGAGACGCGAATGCGTCAAGGACCGCCCAGTGTCTGTCGAACCATCGATTTGCGTCCACA
CCGTCTCTAGACGAACGGCTTTGGGGCCCTCCGTGAGAAGCTTTGCTATGAGAGCTGACGGATc

Supplementary File 4 : O. tauri snoRNP protein homologues

CD snoRNP homologues

>Fibrillarin/Nop1 (ostta07g01200)
MRAPMGRGGGRGGFSPGGRGRGGRDGGRGGGRGGGRGGGRGGPGGRGGGRGGGRGGRGGGRGGRGGMKGGSKVVV
EKHRHEGVFVARGKEDALVTRNMVVGESVYGEKRISVDEADGTKTEYRVWNPFRSKIAAGILGGLDNIHIAPGTK
LLYLGGASGTTVSHCSDLVGPEGTVYAVEFSHRSGRDLVNMAKKRTNVIPIIEDARHPQKYRMLIGMVDTIFADV
AQPDQARIVGLNAQYFLKQGGNFMISIKASCIDSTAAPEAVFAREVKKLQTEGFKPKEQLTLEPYERDHAMVTGI
FRAPSKK

>Nop58 (ostta03g00690)
MLLLFETPAGYSLFKVKDEKKLGDVEKLQDAFSTVEGAKKMVSMKAFSKFENTTEALAAAATLVDSKVGKSLKKF
LTKHAEGETLAVADSKLGAAIKEKLGINCVADSGVMELMRGVRYQLNELIGGLTDADLAPMALGLSHSLSRYKLK
FSPDKVDTMVIQAIGLLDELDKELNTYSMRVREWYGWHFPELTKIIADNMQYAKAAKLMGDRANAANLDFSGILD
EDVEQEVKDAAIISMGTEISEEDLSNIGQLADQVIALSEYRAQLYDYLKARMNAIAPNLTVLVGELVGARLISHA
GSLMNLAKHPASTVQILGAEKALFRALKTKHETPKYGLIYHASLIGQAAPKFKGKISRVLAAKCALSIRVDALGE
SSEATIGVDAREKVEARLRQLEGKSLGEASGVKKLSGDIKKHDKDRNADAPALLTAPRSYNPDKDVTKKEKKEKK
DKKDKKEKRERDGDSPKSAKKSKKSD

>Nop56(ostta08g00480)
MAQFVLFESASGYGLFETLDVDVVGQSLEKVQEQTQDAGKFGKMVKLHGFKPFTSAANALEQINCVSEGVASEDL
QNFLEQNLPRQKDSAKAKYQLGVSDSKLGNSIVESTKIPCVCNDSVGEILRGIRQHFTKFVKGFKGGDYEKAQLG
LAHSYSRAKVKFNVNRSDNMIIQAIALIDTLDKDINTFIMRVREWYGWHFPELVKVCNDNYMYAQLALVIKDKAT
LTDEALPALTKITGDEDKAKEVIEAAKASMGQDISPVDMINIEAFAKRVISLAEYRTSLHNYLSNKMNVVAPNLG
ALIGDIIAARLISHAGSLTNLAKYPASTVQILGAEKALFRALKTKGNTPKYGLIFHSTFIGKANARNKGRISRYL
ANKCSIASRIDCFSDFQTTLFGEKLKEQVEERLAFYDKGTAPRKNIAMMQEVIAEIGPQDGGSKKRSADDAATPS
KKVKKDKKDKKEKSAKKAKKSEK

>Snu13/15.5 Kd protein (ostta02g01210)
MADVNPKAYPLADAQLTITILDIVQQASNYKQLKKGANEATKTLNRGISEFVVLAADTEPLEILLHLPLLAEDKN
VPYVFVPSKQALGRACGVSRPVISCSVTTNEGSQLKQQIQNLKDAIEKLLI

H/ACA snoRNP homologues

> Dyskerin/NAP57/Cbf5 (ostta02g00640)
MTRTEELKIEPQAVTPPLDTSSWPLLLKNYDKLMVRTGHYTPIPCGSSPITRPLAEYLRYGVINLDKPANPSSHE
VVAWLKRILRVEKTGHSGTLDPKVTGSLIVCIDRATRLVKSQQGAGKEYVCVCRLHATPAGGKGAVERGIETLTG
ALFQRPPLISAVKRQLRIRAIYKSKLLEFDEARNLVVFWVSCEAGTYIRTLCVHLGLLLGVGGHMQELRRVRSGI
CSEAKNLSTMHDVLDAMWASDNLKDDGYIRRVVMPLEVLLTNYKRIVIKDSAVNAICYGAKLMLPGLLRYEAGIE
VGNECILMTTKGEAIAIGIAEMTTAVMATCDHGSVAKVKRVVMERDTYPRRWGLGPNSQSKRLSAEGTGIKSSVS
PSEVPKKSDKSAPMKDRKEKKEKKEKKRKLDQ

>Gar1(ostta02g01330)
MRPPSYRGARGSAGRTSTGGRSGLRFDGRGRGRGRSGQFRDEGPPSSLEEIGTFLHACEGEIVCLSTNKKVPYFN
GAVYLENKTQVGKVEEIFGPVNDKMFTVKLIEGVNAESYEKGAKFYISPDKLLPVERFINPVSGGRASGRGRAGR
APGMRGGRGGRGTARGAARGRGGRSRF

>Nop10(ostta05g00690)
MNSASRHFFTRLIARSGVGYGGKMYLMYYTDEKGERVYTLKKTAPDGTPTHSAHPARFSPDDKFSKQRVALKKRF
GLLPTQQPAREL

>Nhp2p (ostta13g00670)
MKTPKSEKREKTPKTPSTPRSPSTPGTARTASEGMKACSAIATPLADVKTTKKILKTVKRAAKAKQVRRGVKEVV
KALKKDVKGFAVIAGDISPIDVITHVPILCEEADVPYVYVHSKEELGAAGMTKRPTSVMLVLKEGAKGSVKMSSE
DKKEFDEMYAKCVEKIQAMSK

Supplementary File 5 : Structure elements of *O. tauri* H/ACA box snoRNAs identified by snoGPS (33) including the ASE element in the stem bulge hybridising to the target rRNA or snRNA sequences.

Ot-HAsno1

5'stem target : 25S.U2157

```
UGCCACUGUUAAUCCCUUCACUGGACUUUCAAUGUCCUUGGAGUUUCAAAUGUGGCAAACAUCAACUCGGGAGUUUCGCUGACAGCACUCGCACGUUUCGGCGGGCAAUGAGUGUCAUCUGAUAGUAGCUCUCGCACAUUU
X1234X LLLLLLL I123I                I321I RRRR   X4321X AAAAAA   X123456X         I123I                     I321I           X654321X CCCCCC
               ---
              /   \
              \   /
               A-U
               C-G
               U.G
               U-A
               C-G
               C U
5'-(N6)GUUAAUC     UUCA(N11)ACA
       |||||||     ||||
       CAAUUAG GY AAGU
```

3'stem target : 25s.U2194

```
UGCCACUGUUAAUCCCUUCACUGGACUUUCAAUGUCCUUGGAGUUUCAAAUGUGGCAAACAUCAACUCGGGAGUUUCGCUGACAGCACUCGCACGUUUCGGCGGGCAAUGAGUGUCAUCUGAUAGUAGCUCUCGCACAUUU
X1234X         I123I                I321I          X4321X HHHHHH    XX  LLLLLLL I1  4567I                     I7654  1I RRRRRRRRXX      AAAAAA
                ---
               /   \
               \   /
                G.U
                A C
                C A
                A-U
                G-C
                U U
5'-(N71)GUUUCGC     GAUAGUAG(N8)ACA
        |||||||     ||||||||
        CAAAGCG AY CUAUCAUC
```

Ot-HAsno4

5'stem targets : 18S.U1370

```
CCGUCGUUCACGUUCUCGAGAUCCCAAUUCAUGGACUGCGAGGAUGGGCGUUUUCACGCCAACGACGGCACAACCAUCCGGGAAAACAAUACCUAUUCCAACUCUGAUUUCUGAGGUUUUGGAAGAUGCUUUUUCUACAUUU
X12345X LLLLL1L  I12  56I                    I65  21I RRRRR  X54321X AAAAAA     X1234X          I1 34 67I         I76 43 1I         X4321XCCCCCC
               ---
              /   \
              \   /
               A G
               G.U
               A-U
               G.U
               C U
               U
5'-(N7)CACGUUC     CACGC(N11)ACA
       ||||| |     |||||
       GUGCACG AY GUGCG
```

5'stem target : 18S. U1508

```
GUUCACGUUCUCGAGAUCCCAAUUCAUGGACUGCGAGGAUGGGCGUUUUCACGCCAACGACGGCACAACCAUCCGGGAAAACAAUACCUAUUCCAACUCUGAUUUCUGAGGUUUUGGAAGAUGCUUUUUCUACAUUU
X12  567X      LLLLLI12 4 6I            I6 4 21I RRRRR  X765  21XAAAAAA     X1234X          I1 34 67I         I76 43 1I         X4321XCCCCCC
              ---
             /   \
             \   /
              C-G
              U C
              U.G
              A-U
              A-U
                U
5'-(N14)AUCCC     UCACG(N12)ACA
        |||||     |||||
        UAGGG GY AGUGC
```

3'stem target : 18S.U120

```
CCGUCGUUCACGUUCUCGAGAUCCCAAUUCAUGGACUGCGAGGAUGGGCGUUUUCACGCCAACGACGGCACAACCAUCCGGGAAAACAAUACCUAUUCCAACUCUGAUUUCUGAGGUUUUGGAAGAUGCUUUUUCUACAUUU
X123456X         I1234 6I                        I6 4321I      X654321X HHHHHH    X123XLLLL1LLLI1I                        I1IRRRR    X321X AAAAAA
                 ---
                /   \
                \   /
                 C U
                 U U
                 U U
                 A-U
                 U.G
                 C-G
5'-(N83)AACAAUAC     AAGA(N11)ACA
        ||||  .||    ||||
        UUGUGGUG GY UUCU
```

Ot-HAsno5

5’stem targets : 18S.U1290

```
CGGCCGCUAGUUCGUUAGCCGUCUUCCCUACUCCAUGGGUCUGGGGGCUCGGAAAGCGGCCGAACAUAAACGUGGUACUCUUUCACGUGCGCGUCGCCGAAAGCUGGCGUAGAACCCGGUAACCAGGACGCGCACAGAUUGAGGCCCGAAAUUU
X123456X LLLLLLLI123 5I                   I5 321IRRRRRX654321X AAAAAA   X12   56X      I123456I                               I654321I      X65   21XCCCCCC
               ---
              /   \
              \   /
               U.G
               G G
               C-G
               C-G
               G-C
               A-U
5'-(N8)GUUCGUU    CGGAA(N10)ACA
       |||||||    |||||
       CAAGCAA UY GCCUU
```

3’stem target : 18S.U1370

```
CGGCCGCUAGUUCGUUAGCCGUCUUCCCUACUCCAUGGGUCUGGGGGCUCGGAAAGCGGCCGAACAUAAACGUGGUACUCUUUCACGUGCGCGUCGCCGAAAGCUGGCGUAGAACCCGGUAACCAGGACGCGCACAGAUUGAGGCCCGAAAUUU
X123456X         I12 4I                   I4 21I      X654321X HHHHHH   X12   56X  LLLLLLLI123I                               I321I RRR     X65   21XAAAAAA
                ---
               /   \
               \   /
                C-G
                U-A
                G-C
                C-G
                G-C
                  G
5'-(N82)CACGUGC    CAC(N14)AAA
        |||||||    |||
        GUGCACG AY GUG
```

Ot-HAsno18

5’stem targets : 25S.U2697

```
GCAUCAUCAUAUCGAAGACUCGGGUGUUGUCCUUUCCAUGAUCGUCCGGGGUCAAAAUUGAUGCUACAUCAAUACCGUUCGCAUCGUAAUGCUUGUCGGCGUCGAUAUCGCCGUCGGGCACGCGAAGCGACCGGGACACAC
X1234X    LLLLLLL I123456I                I654321I RRRRRR X4321X AAAAAA    X1 345X        I1234 678I         I876 4321I       X543 1X  CCCCCC
               ---
              /   \
              \   /
               G-C
               G-C
               C-G
               U.G
               C-G
               A G
5'-(N9)AUCGAAG    UCAAAA(N9)ACA
       |||||||    ||||||
       UAGCUUC CY AGUUUU
```

5’stem : 18S.U2700

```
AUCAUCAUAUCGAAGACUCGGGUGUUGUCCUUUCCAUGAUCGUCCGGGGUCAAAAUUGAUGCUACAUCAAUACCGUUCGCAUCGUAAUGCUUGUCGGCGUCGAUAUCGCCGUCGGGCACGCGAAGCGACCGGGACACAC
X 2345X          LLL I 23 56I        I65 32 I RRRRRRRRR X5432 XAAAAAA    X1 345X         I1234 678I         I876 4321I       X543 1X  CCCCCC
            ---
           /   \
           \   /
            U C
            U.G
            G.U
            U C
            G-C
            G G
5'-(N16)UCG    GGGUCAAAA(N9)ACA
        |||    ||.||||||
        AGC UY CCUAGUUUU
```

3’stem : 18S.U1579

```
AUCAUCAUAUCGAAGACUCGGGUGUUGUCCUUUCCAUGAUCGUCCGGGGUCAAAAUUGAUGCUACAUCAAUACCGUUCGCAUCGUAAUGCUUGUCGGCGUCGAUAUCGCCGUCGGGCACGCGAAGCGACCGGGACACAC
X 2345X         I123456I                I654321I        X5432 XHHHHHH   X12 4X    LLLLLLI1234 678I          I876 4321IRRRR     X4 21X AAAAAA
               ---
              /   \
              \   /
               U U
               G-C
               U.G
               U.G
               C-G
               G-C
5'-(N81)CGUAAU    ACGC(N13)ACA
        ||||||    ||||
        GCAUUA GY UGCG
```

Ot-HAsno20

5’stem targets : 18S.U1598 & U1597

```
GGGCGUCUGUGUGUACGGAUCGCGGGGCGGCGCGAGCCGCUAUCCCGCGAGCCCGAAGGGAUUUCGCGCCCGAUAGUGAAGGGAUCGAUCAUUGUCACGCGCGCGAGCGGGAACGUGACUCGCCGUGCGCCCUUAUUCGGUCCCACAUUU
X1234X  LLLLLLL I1 3456I                   I6543 1I  RRRRR     X4321X AAAAAA  X123456X          I12345I                 I54321I      X654321XCCCCCC
               ---
              /   \
              \   /
               U-A
               A G
               G-C
               G-C
               C C
                 G
5'-(N7)GUGUGUA    AAGGG(N13)AUA
       |||||||    |||||
       CACACAU GY UUCCC

GGGCGUCUGUGUGUACGGAUCGCGGGGCGGCGCGAGCCGCUAUCCCGCGAGCCCGAAGGGAUUUCGCGCCCGAUAGUGAAGGGAUCGAUCAUUGUCACGCGCGCGAGCGGGAACGUGACUCGCCGUGCGCCCUUAUUCGGUCCCACAUUU
X1234X LLLLLLL  I1 345I                    I543 1I   RRRR     X4321X AAAAAA  X123456X          I12345I                 I54321I      X654321XCCCCCC
               ---
              /   \
              \   /
               A G
               G-C
               G-C
               C C
               A G
                 A
5'-(N6)UGUGUGU    AGGG(N13)AUA
       |||||..    ||||
       ACACAUG UY UCCC
```

Ot-HAsno23

5’stem : 25S.U2686

```
CCUUGACAAAGCAGAGGGCUGAUCAGGCUUAGGCCGCAUGUGCUGCUCUCCCGUCGUUCUAAGGUACAGCCGAUAGCGUCGGCAUGGGUGAGUAGGGGGCUGCCUUUCCACAGUUCUCUGCUCAAUCGUCGUCGCGACAUUU
X123X  LLLLL I1234I                                I4321I RRRRR   X321X AAAAAA    X12 456X        I1234567I             I7654321I      X654 21X CCCCCC
             ---
            /   \
            \   /
             G-C
             G.U
             G-C
             A-U
             G-C
             A C
5'-(N6)AAAGC    CGUCG(N10)ACA
       |||||    |||||
       UUUCG UY GCAGC
```

3’stem : 25S.U2755

```
UUGACAAAGCAGAGGGCUGAUCAGGCUUAGGCCGCAUGUGCUGCUCUCCCGUCGUUCUAAGGUACAGCCGAUAGCGUCGGCAUGGGUGAGUAGGGGGCUGCCUUUCCACAGUUCUCUGCUCAAUCGUCGUCGCGACAUUU
X1 3 5X            I1 34I                 I43 1I         X5 3 1X  HHHHHH    X12 45X  LLLLLLI12345678I              I87654321IRRRRR X54 21X AAAAAA
                ---
               /   \
               \   /
                G.U
                G-C
                A-U
                U.G
                G-C
                A-U
5'-(N81)UGGGUG    CAAUC(N10)ACA
        ||||||    |||||
        ACCCAC UY GUUAG
```

HAsno25

5’stem : 18S.U1385 & U1384

```
UUGCGACUUUGCCUAAGGCGCUCCUCACUUAACGGUGGAGAUGAUCUUUUUACUUCCGUCGCAAACAUCAAUCUUUUUCGGUCCGAUGACUCCACGACGACCGAGCCACUUCAUCGGCUCGUCCGUCACUACAUUAAGGACUACAUCU
X12345X LLLLLLI12I                            I21IrRRRRRRX54321XAAAAAA          X1X            I12  5678I           I8765  21I              X1X  CCCCCC
              ---
             /   \
             \   /
              G.U
              C C
              G.U
              G.U
              A-U
              A-U
5'-(N7)UUGCCU    UACUUCC(N8)ACA
       ||||||     ||||||
       AACGGA GY UUGAAGG

UUGCGACUUUGCCUAAGGCGCUCCUCACUUAACGGUGGAGAUGAUCUUUUUACUUCCGUCGCAAACAUCAAUCUUUUUCGGUCCGAUGACUCCACGACGACCGAGCCACUUCAUCGGCUCGUCCGUCACUACAUUAAGGACUACAUCU
X12345X LLLLLlI12 4 6I                      I6 4 21I RRRRRRX54321XAAAAAA          X1X            I12  5678I           I8765  21I              X1X  CCCCCC
               ---
              /   \
              \   /
               G-C
               C U
               G.U
               G.U
               A-U
                 U
5'-(N7)UUGCCUA    ACUUCC(N8)ACA
       ||||||     ||||||
       AACGGAG UY UGAAGG
```

3’stem : 18S.1261

```
UUGCGACUUUGCCUAAGGCGCUCCUCACUUAACGGUGGAGAUGAUCUUUUUACUUCCGUCGCAAACAUCAAUCUUUUUCGGUCCGAUGACUCCACGACGACCGAGCCACUUCAUCGGCUCGUCCGUCACUACAUUAAGGACUACAUCU
X12345X        I12 4 6I                    I6 4 21I       X54321XHHHHHH         X123X    LLLLLLLI123  678I             I876  321IRRRR       X321XAAAAAA
                ---
               /   \
               \   /
                C U
                A C
                G-C
                C-G
                A-U
                G-C
5'-(N87)ACUCCAC    ACUA(N12)ACA
        |||||||    ||||
        UGAGGUG GY UGAU
```

HAsno26

5’stem : 25S.U873

```
UGCGCUCUACCGAUACGCCGUAACCGUUUCUCUCGAGAAUUUGUUGGUUGCGGCUCAACUGCGAGCGCAUAUAAAAACGUGAAUGCUUUGAACGGGCGACCAGCCCACCUUGGUUUCGCAGCCGAGUACUUCUGGACCCGCCCUUUGCACGCAUUGUAAAUUC
X12345X  LLLLL  I123456I                        I654321I  RRRRRRX54321X AAAAAA     X123X           I 234I                           I432 I              X321X  CCCCCC
            ---
           /   \
           \   /
            G-C
            C-G
            C-G
            G-C
            C U
            A C
5'-(N8)CCGAU    AACUGC(N9)AUA
       |||||    ||||||
       GGCUA UY UUGACG
```

3’stem : 25S.U772,

```
GCGCUCUACCGAUACGCCGUAACCGUUUCUCUCGAGAAUUUGUUGGUUGCGGCUCAACUGCGAGCGCAUAUAAAAACGUGAAUGCUUUGAACGGGCGACCAGCCCACCUUGGUUUCGCAGCCGAGUACUUCUGGACCCGCCCUUUGCACGCAUUGUAAAUUC
X1234X         I12345678I                    I87654321I        X4321XHHHHHH       X12X    LLLLI123I                                                    I321IRRRRRRRRX21X  AAAAAA
            ---
           /   \
           \   /
            A C
            G-C
            C-G
            G-C
            G-C
            G-C
5'-(N87)GAAC    UUUGCACG(N7)AAA
        ||||    ||||||||
        CUUG CY AAACGUGC
```

Ot-HAsno27

5' stem   25S.U2109 39.22

```
GCCAGGUCCUUAAGAACGCUCUCCGUCGCAUCGCUUCCGUGAUGUAUGGAGAGAGAUCGAGUCAUAACUUGGCAACAUUAAAACGCAUCGCGACACGUAUUGUGUCGCUUCGUGCGUUAGCGUAAUCGCUCGUUUUCGUGAAGUGUGGGAGAUUCGUCGAGCUGACCUGCUUCACUCAGAGCGUGCCCGCGGACAUUU
X1234X LLLLLLI1 3 5678I                         I8765 3 1IRRRRRRRR X4321X AAAAAA                                           X1X           I12345I                          I54321I          X1X  CCCCCC
             ---
            /   \
            \   /
             C-G
             G A
             C-G
             A A
             A-U
             G-C
5'-(N6)CCUUAA    GAGUCAUA(N9)ACA
       ||||||    ||||||||
       GGAAUU CY CUCAGUAU
```

5' stem   25S.U2107 38.72

```
GCCAGGUCCUUAAGAACGCUCUCCGUCGCAUCGCUUCCGUGAUGUAUGGAGAGAGAUCGAGUCAUAACUUGGCAACAUUAAAACGCAUCGCGACACGUAUUGUGUCGCUUCGUGCGUUAGCGUAAUCGCUCGUUUUCGUGAAGUGUGGGAGAUUCGUCGAGCUGACCUGCUUCACUCAGAGCGUGCCCGCGGACAUUU
X12345XLLLLLLLL I1 345I                              I543 1I RRRRRRX54321X AAAAAA                                           X1X           I12345I                          I54321I          X1X  CCCCCC
               ---
              /   \
              \   /
               C-G
               U-A
               C U
               G-C
               C-G
               A A
5'-(N6)CCUUAAGA    GUCAUA(N9)ACA
       ||||||||    ||||||
       GGAAUUCU CY CAGUAU
```

3' stem   25S.U1228 27.20

```
GCCAGGUCCUUAAGAACGCUCUCCGUCGCAUCGCUUCCGUGAUGUAUGGAGAGAGAUCGAGUCAUAACUUGGCAACAUUAAAACGCAUCGCGACACGUAUUGUGUCGCUUCGUGCGUUAGCGUAAUCGCUCGUUUUCGUGAAGUGUGGGAGAUUCGUCGAGCUGACCUGCUUCACUCAGAGCG
X12345X         I1 345I                              I543 1I       X54321X HHHHHH                                          X1X      LLLLLLLLI12I                           I21IRRRR       X1X  AAAAAA
                 ---
                /   \
                \   /
                 U.G
                 U U
                 G-C
                 C-G
                 U-A
                 C-G
5'-(N119)CGUAAUCG    CUGA(N13)AGA
         ||.|||||    ||||
         GCGUUAGC GY GACU
```

3' stem   25S.U742 22.74

```
GCCAGGUCCUUAAGAACGCUCUCCGUCGCAUCGCUUCCGUGAUGUAUGGAGAGAGAUCGAGUCAUAACUUGGCAACAUUAAAACGCAUCGCGACACGUAUUGUGUCGCUUCGUGCGUUAGCGUAAUCGCUCGUUUUCGUGAAGUGUGGGAGAUUCGUCGAGCUGACCUGCUUCACUCAGAGCGUGCCCGCGGACAUUU
X12345X         I1 345I                              I543 1I       X54321X HHHHHH                                                       X123X        LLL1LLLI1 345I      I543 1IRRRRRR      X321XAAAAAA
            ---
           /   \
           \   /
            G-C
            A-U
            G.U
            C C
            U-A
            G-C
5'-(N147)GAGAUUC    UCAGAG(N12)ACA
         ||| .||    .|||||
         CUCGGAG GY GGUCUC
```

Ot-HAsno29

5' stem   25S.U1830 34.28

```
GCGAGUUUACCCCCGCCCUGGCGCUCACAUCGCCUCCGGCGGUCCUCAACACCUCGCUACAAUAAAGAUGGACUUUUUAGGACACCUCCGAGAAACACUUCGGAGCCUUUCCCCGUUAAACACCG
X123X   LLLLI123I                       I321IRRRRRR    X321X AAAAAA    X 23X             I12345I      I54321I          X32 XCCCCCC
          ---
         /   \
         \   /
          C C
          C-G
          C-G
          G-C
          C-G
          C-G
5'-(N7)ACCC     UCCUCA(N11)ACA
       ||||     .|||||
       UGGG UY GGGAGU
```

3' stem   18S.U263 21.18

```
GCGAGUUUACCCCCGCCCUGGCGCUCACAUCGCCUCCGGCGGUCCUCAACACCUCGCUACAAUAAAGAUGGACUUUUUAGGACACCUCCGAGAAACACUUCGGAGCCUUUCCCCGUUAAACACCGGGUCGGCGACGUCUCACGCCUGCGCGUUGUGCCAAGGGGCUACAAUU
X123X          I 234I              I432 I           X321X HHHHHH                                                                   X 2 4X    LLLLLI12I           I21I RrRRRRRX4 2 XAAAAAA
               ---
              /   \
              \   /
               C C
               U.G
               G-C
               C-G
               A-U
                 U
5'-(N127)CGGCG    GUGCCAA(N7)ACA
          |||||   . |||||
         GCCGC GY UCCGGUU
```

Ot-HAsno32

5' stem   18S.U971 48.05

```
UCCCGGCGUAUCUAUAUUACCGUCUCACCCGUGGCGGUCGUACUCAUGAUCCGGGGUACAUCGAACAGCUCGUCGGCUUGCGACGAGCGCGCGGCGCGAGCCACGACGCGCGCUCUACUUUUUGCCACGAACAUUU
X1234X LLLLLL I12 4567I          I7654 21I RRRR   X4321X AAAAAA       X12X           I1234567I             I7654321I            X21XCCCCCC
            ---
           /   \
           \   /
            A-U
            U C
            U.G
            A-U
            U-A
            A C
5'-(N6)GUAUCU     UCAU(N11)ACA
       ||||||     ||||
       CAUAGA UY AGUA
```

3' stem   18S.U1088 46.46

```
UCCCGGCGUAUCUAUAUUACCGUCUCACCCGUGGCGGUCGUACUCAUGAUCCGGGGUACAUCGAACAGCUCGUCGGCUUGCGACGAGCGCGCGGCGCGAGCCACGACGCGCGCUCUACUUUUUGCCACGAACAUUU
X1234X         I1 34567I        I76543 1I         X4321X HHHHHH      X12X   LLLLLLLLI1234567I              I7654321IRRRR        X21XAAAAAAA
                  ---
                 /   \
                 \   /
                  C-G
                  G-C
                  C-G
                  G-C
                  A-U
                  G-C
5'-(N75)CUUGCGAC     UACU(N12)ACA
        ||||||||     ||||
        GAACGCUG GY AUGA
```

Ot-HAsno35

5' stem   18S.U585 36.78

```
GCGCCUCUUUAGAGACUCGACGAGCCCCUCACUCGUUUUGUUUUAUGCUGGGGCGCGAAAUAAACGCGCGAUCGCGAUUGGAUGGUGACCUCUUUCGCGGUCUCUCUUCCGCUCGAGGUCGCUACAAUU
X12345X LLLLLI12  567I            I765  21I RRRRRRX54321X AAAAAA    X12345 7X       I1 345I        I543 1I         X7 54321X CCCCCC
           ---
          /   \
          \   /
           C U
           U U
           C-G
           A-U
           G.U
             U
5'-(N7)UUAGA     UAUGCU(N9)AAA
       |||.|     ||.|||
       AAUUU AY AUGCGA
```

3' stem 18S.U173 41.29

```
GCGCCUCUUUAGAGACUCGACGAGCCCCUCACUCGUUUUGUUUUAUGCUGGGGCGCGAAAUAAACGCGCGAUCGCGAUUGGAUGGUGACCUCUUUCGCGGUCUCUCUUCCGCUCGAGGUCGCUACAAUU
X12345X           I1234I            I4321I       X54321X HHHHHH    X12XLLL1LLLLI12 45 78I          I87 54 21IRRRrRR   X21X AAAAAA
                ---
               /   \
               \   /
                G-C
                G.U
                U C
                A-U
                G.U
                G-C
5'-(N70)UCGCGAUU    CGCUCG(N9)ACA
          ||| ||||    ||| ||
        AGCCCUAA AY GCGUGC
```

Ot-HAsno54

5' stem 25S.U1483 36.95

```
CGCGUGGGACGGGUUCAUUGAUCGAGGCCGGCUUGAUACCCCGGCUGUGCUCUGAGCCAUCAAUUUUUUAAACGCGCGACAUUAACACCUGGGAAUAUAUCGGGGUUCCCGCCGUAUCAGUCGAUGUCGGAACCGUGAGCGAUCCUUGGGACAUUU
X1234X  LLLLLI1 34 6 8I                              I8 6 43 1IRRRR     X4321XAAAAAA   X12345 7X       I1234I                  I4321I       X7 54321XCCCCCC
          ---
         /   \
         \   /
          U C
          U-A
          A-U
          C C
          U-A
          U-A
5'-(N7)ACGGG    UUUU(N12)ACA
        |||||    ||||
        UGCCC UY AAAA
```

3' stem 18S.U1620 39.02

```
CGCGUGGGACGGGUUCAUUGAUCGAGGCCGGCUUGAUACCCCGGCUGUGCUCUGAGCCAUCAAUUUUUUAAACGCGCGACAUUAACACCUGGGAAUAUAUCGGGGUUCCCGCCGUAUCAGUCGAUGUCGGAACCGUGAGCGAUCCUUGGGACAUUU
X1234X          I1234I                                    I4321I        X4321XHHHHHH   X1 345X    LLLLLI12345 7I             I7 54321I rRRRRRRX543 1X AAAAAA
           ---
          /   \
          \   /
           C-G
           U-A
           U-A
           G-C
           G-C
             G
5'-(N97)AUCGG    UGAGCGA(N9)ACA
         |||||     ||||||
         UAGCC AY CCUCGCU
```

Ot-HAsno56

5' stem 18S.U1480 21.00

```
CCGACUUUCUUACCUGUGACUUCGCUCUUUUUGUUGUGAGCAUUGUUGCCUUAUAAAGGGUCGGAGAUUAAUUUACAGGGCGCCUUUACCAAUAUCUGGUCGAAACCUUCGGGUCAUGGCUAAGUUAUAAUGGCUUGGUGUCCACAUUC
X1234X   LLLLLLLI12I                        I21IRRRR      X4321XAAAAAA                X1234X           I1I                  I1I        X4321X    CCCCCC
             ---
            /   \
            \   /
             U U
             U U
             C-G
             A-U
             G.U
             U.G
5'-(N8)UUACCUG    CCUU(N13)AGA
        |||||..    ||||
        AAUGGGU CY GGAA
```

3' stem 25S.U995 38.53

```
CCGACUUUCUUACCUGUGACUUCGCUCUUUUUGUUGUGAGCAUUGUUGCCUUAUAAAGGGUCGGAGAUUAAUUUACAGGGCGCCUUUACCAAUAUCUGGUCGAAACCUUCGGGUCAUGGCUAAGUUAUAAUGGCUUGGUGUCCACAUUC
X123456X       I123I                          I321I       X654321XHHHHHH       X123X   LLLLLLI12 4I                         I4 21IRRRRRR    X321XAAAAAA
             ---
            /   \
            \   /
             U-A
             C-G
             U U
             A-U
             U-A
             A-U
5'-(N84)UUACCA    AAUGGC(N10)ACA
          ||||||    ||||||
         AAUGGU UY UUACCG
```

3' stem 25S.U996 35.9

```
CCGACUUUCUUACCUGUGACUUCGCUCUUUUUGUUGUGAGCAUUGUUGCCUUAUAAAGGGUCGGAGAUUAAUUUACAGGGCGCCUUUACCAAUAUCUGGUCGAAACCUUCGGGUCAUGGCUAAGUUAUAAUGGCUUGGUGUCCACAUUC
X123456X       I123I                          I321I       X654321XHHHHHH       X123X   LLLLLLI12 4I                         I4 21IRRRRRR    X321XAAAAAA
             ---
            /   \
            \   /
             U-A
             C-G
             U U
             A-U
             U-A
             A-U
5'-(N84)UUACCA    AAUGGC(N10)ACA
          ||||||    ||||||
         AAUGGU UY UUACCG
```

Ot-HAsno58

5' stem   18S.U720 46.23

```
GCAUCUUUCAAAGCUCCGAGGUUAAUCUCACGAAAUGCCCCUUGGAGACUACAAGAAUGCUACAGAGAAAGGCCUGCGCACUAAUACUCGCGGGAGUUUAUUCACUCACGUGGGUUACGCUUCGUCGGUACAUUC
X12X LLLLLLLI123456I                  I654321IRRRRR    X21X   AAAAAA  X12 45X        I12345 7I          I7 54321I         X54 21XCCCCCC
               ---
              /   \
              \   /
               A-U
               G.U
               C-G
               C-G
               U-A
               C-G
5'-(N4)UUUCAAAG    ACUAC(N12)AGA
       ||||||||    ||.||
       AAAGUUUC AY UGGUG
```

5' stem   18S.U888 43.46

```
GCAUCUUUCAAAGCUCCGAGGUUAAUCUCACGAAAUGCCCCUUGGAGACUACAAGAAUGCUACAGAGAAAGGCCUGCGCACUAAUACUCGCGGGAGUUUAUUCACUCACGUGGGUUACGCUUCGUCGGUACAUUC
XX  LLLLLLlL I123456I                  I654321I  RrRRRRRRXX    AAAAAA  X12 45X        I12345 7I          I7 54321I         X54 21XCCCCCC
               ---
              /   \
              \   /
               C-G
               C-G
               U-A
               C-G
               G A
                  C
5'-(N3)CUUUCAAA    UACAAGAAU(N6)AGA
       |||||| |    | |||||||
       GAAAGUAU UY AGGUUCUUA
```

3' stem   25S.U882 44.11

```
GCAUCUUUCAAAGCUCCGAGGUUAAUCUCACGAAAUGCCCCUUGGAGACUACAAGAAUGCUACAGAGAAAGGCCUGCGCACUAAUACUCGCGGGAGUUUAUUCACUCACGUGGGUUACGCUUCGUCGGUACAUUC
X123 5X        I1234I                  I4321I        X5 321X   HHHHHH  X12 4X   LLLLLI123456 8I          I8 654321IRrRRRRR X4 21XAAAAAA
              ---
             /   \
             \   /
              C-G
              G.U
              C-G
              U.G
              C-G
              A-U
5'-(N79)CUAAU    UACGCUU(N8)ACA
        |||||    | |||||
        GAUUA GY AAGCGAA
```

Ot-HAsno59

5' stem   18S.U734 46.35

```
GCAAGCUACACUCUGUCGUGAGACCUCUUCAUGUCACAUUGCAAUUUUUAGGCUUGCGACAUCAAACAGCGGAUCGGAUUUGCCGGCGGGGCAGGUGUUUUUAGUCACGGUCUCUCUCUUCCUACCAUUUGAUCCACAUUC
X1234X LLLLLI12 456 8I          I8 654 21IRRRRRR  X4321X AAAAAA      X1234567X        I123 56I                 I65 321I      X7654321XCCCCCC
             ---
            /   \
            \   /
             U-A
             G.U
             C U
             U.G
             G-C
             U-A
5'-(N6)ACACUC    AUUUUU(N10)ACA
       ||||||    ||||||
       UGUGAG AY UAAAAA
```

5' stem   YourSeq.U733 39.08

```
GCAAGCUACACUCUGUCGUGAGACCUCUUCAUGUCACAUUGCAAUUUUUAGGCUUGCGACAUCAAACAGCGGAUCGGAUUUGCCGGCGGGGCAGGUGUUUUUAGUCACGGUCUCUCUCUUCCUACCAUUUGAUCCACAUUC
X1234X LLLLLLL I 234I                 I432 IRRRRR  X4321X AAAAAA      X1234567X        I123 56I                 I65 321I      X7654321XCCCCCC
              ---
             /   \
             \   /
              G.U
              U.G
              G-C
              C A
              U-A
              G
5'-(N6)ACACUCU    UUUUU(N10)ACA
       |||||||    |||||
       UGUGAGA UY AAAAA
```

5' stem   U6.U38 40.84

```
GCAAGCUACACUCUGUCGUGAGACCUCUUCAUGUCACAUUGCAAUUUUUAGGCUUGCGACAUCAAACAGCGGAUCGGAUUUGCCGGCGGGGCAGGUGUUUUUAGUCACGGUCUCUCUCUUCCUACCAUUUGAUCCACAUUC
X12345X   LLLLLI 234I                 I432 IRRRRRRX54321X AAAAAA      X1234567X        I123 56I                 I65 321I      X7654321XCCCCCC
            ---
           /   \
           \   /
            A-U
            G.U
            U.G
            G-C
            C A
            U-A
5'-(N9)CUCUG    UUUUUA(N9)ACA
       |||||    ||||||
       GAGAC AY AAAAAU
```

Ot-HAsno63

5' stem   25S.U1918 39.34

```
GCUGGUGGUAUCGUUGCCUGCGCUUGAGCUCCGCGAUGGGGCGCAUGGCUUGUUCCCCAGCAACAUCAAAAGAGACUUGGUGACAGGUGCUCAUGCUUACCAGCGAGCAUACGAUCACUAGGACUCGACACGC
X1234  6X   LLLLLLLI12345I               I54321I RRR    X6 4321X AAAAAA   X12 4567X      I1234I              I4321I       X7654 21X CCCCCC
                ---
               /   \
               \   /
                C-G
                G-C
                C-G
                G-C
                U-A
                  U
5'-(N9)UCGUUGCC    GGC(N14)ACA
       ||||.|||    |||
       AGCAGCGG AY CCG
```

3' stem   25S.U651 33.75

```
GCUGGUGGUAUCGUUGCCUGCGCUUGAGCUCCGCGAUGGGGCGCAUGGCUUGUUCCCCAGCAACAUCAAAAGAGACUUGGUGACAGGUGCUCAUGCUUACCAGCGAGCAUACGAUCACUAGGACUCGACACGC
X1234  6X        I 234567I               I765432 I      X6 4321X HHHHHH   X12 4X     LLLLI12  5 78I      I87 5  21IRRRRR   X4 21X AAAAAA
             ---
            /   \
            \   /
             C-G
             U C
             C A
             G.U
             U-A
             G-C
5'-(N81)ACAG    GAUCA(N11)ACA
        ||||    |||||
        UGUC UY CUAGU
```

Ot-HAsno65

5' stem   U2.U43 42.08

```
GGGAGCUAAUCGUGAAGCCGGCCCGCGAGAGAUGUCGAGGCGUCGGUGAUACUACCUCCCAACAACAAAACCGAGCGACAAUAGAACCGACGAUUCUGAGCCAUGACCGUCGGUCUACACAUCGUUCCACAUAC
X123X        LLLI1234I                   I4321IRRRRRRRX321X AAAAAA     X1234X          I1234 6I          I6 4321I          X4321X CCCCCC
           ---
          /   \
          \   /
           C-G
           G.U
           G-C
           C-G
           C-G
           G.U
5'-(N12)GAA    GAUACUAC(N7)ACA
        |||    ||||||||
        CUU GY CUAUGAUG
```

5' stem   25S.U2753 36.27

```
GGGAGCUAAUCGUGAAGCCGGCCCGCGAGAGAUGUCGAGGCGUCGGUGAUACUACCUCCCAACAACAAAACCGAGCGACAAUAGAACCGACGAUUCUGAGCCAUGACCGUCGGUCUACACAUCGUUCCACAUAC
X123X      LLLLLI1234I                   I4321I RRrRRRRX321X AAAAAA     X1234X          I1234 6I          I6 4321I          X4321X CCCCCC
             ---
            /   \
            \   /
             G.U
             G-C
             C-G
             C-G
             G.U
               G
5'-(N10)GUGAA    AUACUAC(N7)ACA
        |||||    || ||||
        CACUU GY UAGGAUG
```

3' stem U2.U39 44.87

```
GGGAGCUAAUCGUGAAGCCGGCCCGCGAGAGAUGUCGAGGCGUCGGUGAUACUACCUCCCAACAACAAAACCGAGCGACAAUAGAACCGACGAUUCUGAGCCAUGACCGUCGGUCUACACAUCGUUCCACAUAC
X1234  6X        I123I                   I321I      X6 4321X HHHHHH     X123X  LLLLLLI123456 8I         I8 654321IRRRRRR  X321X AAAAAA
              ---
             /   \
             \   /
              C-G
              A-U
              G-C
              C-G
              C-G
              A-U
5'-(N78)AAUAGA    CUACAC(N9)ACA
        ||.|||    ||||||
        UUGUCU AY GAUGUG
```

Ot-HAsno66

5' stem U1.U91 27.10

```
UGCGGGACGAACGACGCGCGCGAGGGCGACGAUUGGACGACCCUAAACCUAAAACCUGGAUAGGGCCCGCGCGCGGAGGACAGUGAAAAAGCGAUCGCGCACGGUGGACACCCGGAUGAAAAUCGAGAGACUGCGAGAACGCGACGACUGACGUGUGAUCGCGCGAUAUUG
X 2X LLLLLLL I123 5I                                                  I5 321I RRR        X2 XAAAAAA    X12 456X       I12 4I                          I4 21I       X654 21XCCCCCC
              ---
             /   \
             \   /
              C C
              G-C
              C-G
              G-C
              C-G
              A C
5'-(N4)GACGAACG    GCG(N13)AAA
       ||||||||    |||
       CUGCUUGC UY CGC
```

5' stem U1.U95 22.21

```
ACGAACGACGCGCGCGAGGGCGACGAUUGGACGACCCUAAACCUAAAACCUGGAUAGGGCCCGCGCGCGGAGGACAGUGAAAAAGCGAUCGCGCACGGUGGACACCCGGAUGAAAAUCGAGAGACUGCGAGAACGCGACGACUGACGUGUGAUCGCGCGAUAUUG
XX   LLLLLI1234I                                                 I4321IRRRRR    XX   AAAAAA    X12 456X       I12 4I                          I4 21I       X654 21XCCCCCC
            ---
           /   \
           \   /
            G-C
            C-G
            G-C
            C-G
            G-C
            C-G
5'-(N4)CGACG    CGGAG(N10)AAA
       |||||    ||.||
       GCUGC UY GCUUC
```

5' stem 18S.U804 21.56

```
UGCGGGACGAACGACGCGCGCGAGGGCGACGAUUGGACGACCCUAAACCUAAAACCUGGAUAGGGCCCGCGCGCGGAGGACAGUGAAAAAGCGAUCGCGCACGGUGGACACCCGGAUGAAAAUCGAGAGACUGCGAGAACGCGACGACUGACGUGUGAUCGCGCGAUAUUG
X 2X LLL1LLLL I1234I                                                   I4321I RrRRRRRRRX2 XAAAAAA    X12 456X       I12 4I                          I4 21I       X654 21XCCCCCC
              ---
             /   \
             \   /
              C-G
              G-C
              C-G
              G-C
              C-G
              A C
5'-(N4)GACGAACG    GGAGGACAG(N5)AAA
       ||| ||||    . |||||||
       CUGGUUGC UY UAUCCUGUC
```

Ot-HAsno74

5' stem 18S.U1264 30.56

```
AUCGUGGCGAGCACUCGUGCGAAACGCGAAACGGGGUGGAUAUCCAAUCGUGGUGGUACAUUCGAUUGAUGCCAGCAUUGUCGGCGUUCGUGUCUUUUCGCUCGCAACGUACUGCGCUUCGCAGUGGCUCGAACCGCCUAGAGUGUCGGUGCACAUUC
X1234 6X   LLLLLI1I                    I1IRRRR  X6 4321XAAAAAA                                  X123 56X       I12 456I        I654 21I      X65 321X CCCCCC
              ---
             /   \
             \   /
              A G
              G G
              C A
              G.U
              U-A
              G.U
5'-(N10)CACUC    CCAA(N11)ACA
        |||||    ||||
        GUGAG GY GGUU
```

Ot-HAsno81

5' stem 25S.U2737 46.13

```
GGUGCUCUAUAAUCCUAACGCACGAGGCGAAGAGCCACGUCUCGAUGCGUCUUGAAGAGCACCUACAUCGACCGCGGGAUUAGGCCCCCGCACCGCGAGAACGCGGGGUGAUCGCGUGCGAUCGCUUCGGCGUCGGCUUCGACGCUCGACGCACGUUUGAGCGAGGGAGACGACGACGGCGGGACAUCU
X123456X  LLLLLLLI123I                          I321IRRRR X654321X AAAAAA                                          X12 4567X       I 234I            I432 I      X7654 21X CCCCCC
              ---
             /   \
             \   /
              C A
              A-U
              C-G
              G-C
              C-G
              A-U
5'-(N9)AAUCCUA    CUUG(N11)ACA
       |||||||    ||||
       UUAGGAU GY GAAC
```

3' stem 25S.U2509 27.05

```
GGUGCUCUAUAAUCCUAACGCACGAGGCGAAGAGCCACGUCUCGAUGCGUCUUGAAGAGCACCUACAUCGACCGCGGGAUUAGGCCCCCGCACCGCGAGAACGCGGGGUGAUCGCGUGCGAUCGCUUCGGCGUCGGCUUCGACGCUCGACGCACGUUUGAGCGAGGGAGACGACGACGGCGGGACAUCU
X123456X        I12I                    I21I      X654321X HHHHHH                                      X 2 456 8X     LLLLLL I12I              I21I RRRR   X8 654 2 XAAAAAA
               ---
              /   \
              \   /
               A G
               G-C
               C-G
               U-A
               C-G
               G G
5'-(N136)UUCGAC    GAGA(N14)ACA
         |||.||    ||||
         AAGUUG UY CUCU
```

Ot-HAsno83

5' stem 25S.U2421 53.61

```
GCGCCAUCGACCUCACCGGAGCAUGUGCUCUGUGCCAUGUUCCUGAUGUCUUUGGCGCUACAACGACGGGCCAUCUUCCAUCUUGCCGCUUCCUCACGUGAUGCGUCGAGCCAUAAUUGGUCUACAUUU
X1234X   LLLL1LI 2345678I          I8765432 IRRRRRRX4321X AAAAAA  X1234X         I12 456 8I       I8 654 21I        X4321XCCCCCC
              ---
             /   \
             \   /
              G.U
              A-U
              G-C
              G-C
              C U
              C-G
5'-(N8)ACCUCA    AUGUCUU(N8)ACA
       |||| |    |||||||
       UGGACU GY UACAGAA
```

5' stem   25S.U2420 41.65

```
GCGCCAUCGACCUCACCGGAGCAUGUGCUCUGUGCCAUGUUCCUGAUGUCUUUGGCGCUACAACGACGGGCCAUCUUCCAUCUUGCCGCUUCCUCACGUGAUGCGUCGAGCCAUAAUUGGUCUACAUUU
X1234X   LLLL1LLI1I                     I1I RRRRRRX4321X AAAAAA  X1234X         I12 456 8I       I8 654 21I        X4321XCCCCCC
               ---
              /   \
              \   /
               G.U
               A C
               G-C
               G.U
               C-G
                 A
5'-(N8)ACCUCAC    UGUCUU(N8)ACA
       |||| ||    ||||||
       UGGACUG UY ACAGAA
```

3' stem   25S.U2653 32.84

```
GCGCCAUCGACCUCACCGGAGCAUGUGCUCUGUGCCAUGUUCCUGAUGUCUUUGGCGCUACAACGACGGGCCAUCUUCCAUCUUGCCGCUUCCUCACGUGAUGCGUCGAGCCAUAAUUGGUCUACAUUU
X1234X          I123456I          I654321I         X4321X HHHHHH  X1234X         LLLI12 4I       I4 21I RRRRRRR    X4321XAAAAAA
            ---
           /   \
           \   /
            U-A
            U U
            C-G
            G-C
            C-G
              U
5'-(N82)UGC     CGAGCCA(N11)ACA
         |||     |.|||||
        ACG GY GUUCGGU
```

Ot-HAsno84

5' stem   25S.U2613 43.45

```
CGCCGCCGCCAGACGCUUCCUCCGAGACGUGCCGGUCGUCUCCGAGGUGUCUGGUGGUGGCGUACAGCCAACUCGUCGAUCUGUUGAGUCUCUCCCAUGGUACAAGUCUUUGGGAGAAUAAUUUAUGUUCGGCACAUCU
X123456X   LLLLLLLLI12 45678I       I87654 21IRRRRR   X654321X AAAAAA     X123X           I12345I              I54321I           X321XCCCCCC
                ---
               /   \
               \   /
                A-U
                G-C
                C C
                C-G
                U-A
                C-G
5'-(N10)GACGCUUC    GUGUC(N13)ACA
        |||.|.||    |.|||
        CUGUGGAG AY CGCAG
```

3' stem   18S.U1158 49.33

```
CGCCGCCAGACGCUUCCUCCGAGACGUGCCGGUCGUCUCCGAGGUGUCUGGUGGUGGCGUACAGCCAACUCGUCGAUCUGUUGAGUCUCUCCCAUGGUACAAGUCUUUGGGAGAAUAAUUUAUGUUCGGCACAUCU
X123456X      I123 567I           I765 321I       X654321X HHHHHH     X123X  LLLLLLL I12345I              I54321I  RRRR     X321XAAAAAA
                ---
               /   \
               \   /
                C-G
                U-A
                C-G
                U-A
                C A
                  U
5'-(N77)UGUUGAGU    AAUU(N11)ACA
        ||||||||    ||||
        ACAACUCA GY UUAA
```

3' stem   18S.U1157 47.25

```
CGCCGCCAGACGCUUCCUCCGAGACGUGCCGGUCGUCUCCGAGGUGUCUGGUGGUGGCGUACAGCCAACUCGUCGAUCUGUUGAGUCUCUCCCAUGGUACAAGUCUUUGGGAGAAUAAUUUAUGUUCGGCACAUCU
X123456X      I123 567I           I765 321I       X654321X HHHHHH     X123X   LLLLLLLI123456 8I       I8 654321IRR           X321XAAAAAA
                ---
               /   \
               \   /
                C-G
                C-G
                C-G
                U-A
                C-G
                U-A
5'-(N78)GUUGAGUC    AU(N15)ACA
        ||||||||    ||
        CAACUCAG UY UA
```

Ot-HAsno100

5' stem 18S.U177 32.77

```
CCACGACCAAGUCAGCUGCCGAGCGUUCACAUCUCACCGCUCGGCGCGUUACGCUCGUGGAACAUUAAAUGCUCGAGUCGCAAAAUCAACGCUUCGUCGCCGUUUUGGCGAUCGUGAGCGUCGAUCUCGGGCGCACGCACCGCCUUGAGGGGCGUCGGGGACGUUUAGAAGGCACUCGUGCCUCGCGCCGCGACGGGGACCUCAAGGGGUGGGACAUUU
X1X     LLLLL1L I1234567I           I7654321I rRRRRRRrRRX1X AAAAAA          X12X          I123456I                                                                                                            I654321I          X21X CCCCCC
               ---
              /   \
              \   /
               G-C
               C-G
               C-G
               G-C
               U.G
               C C
5'-(N7)AAGUCAG     GUUACGCUCG(N5)ACA
       ||||| |      |||||| ||
       UUCAGCC CY AAAUGCGUGC
```

5' stem U3.U154 34.82

```
CCACGACCAAGUCAGCUGCCGAGCGUUCACAUCUCACCGCUCGGCGCGUUACGCUCGUGGAACAUUAAAUGCUCGAGUCGCAAAAUCAACGCUUCGUCGCCGUUUUGGCGAUCGUGAGCGUCGAUCUCGGGCGCACGCACCGCCUUGAGGGGCGUCGGGGACGUUUAGAAGGCACUCGUGCCUCGCGCCGCGACGGGGACCUCAAGGGGUGGGACAUUU
X1234X       LLL I123456I           I654321IRRRRrRRRRX4321X AAAAAA          X12X          I123456I                                                                                                            I654321I          X21X CCCCCC
            ---
           /   \
           \   /
            A-U
            G-C
            C-G
            C-G
            G-C
            U
5'-(N12)AGC    GCGUUACGC(N8)ACA
        |||    |||. ||||
        UCG AY CGCGCUGCG
```

3' stem 18S.U226 30.63

```
CCACGACCAAGUCAGCUGCCGAGCGUUCACAUCUCACCGCUCGGCGCGUUACGCUCGUGGAACAUUAAAUGCUCGAGUCGCAAAAUCAACGCUUCGUCGCCGUUUUGGCGAUCGUGAGCGUCGAUCUCGGGCGCACGCACCGCCUUGAGGGGCGUCGGGGACGUUUAGAAGGCACUCGUGCCUCGCGCCGCGACGGGGACCUCAAGGGGUGGGACAUUU
X1234X          I1234567I           I7654321I       X4321X HHHHHH          X 2X          LLLLLLLI1234 678I                                                                                                  I876 4321IRRRR          X2 XAAAAAA
                ---
               /   \
               \   /
                U C
                U.G
                U-A
                G-C
                C-G
                C-G
5'-(N91)UUCGUCG    GGAC(N14)ACA
        |||..||    |.||
        AAGUGGC GY CUUG
```

Ot-HAsno104

5' stem 18S.U169 35.33

```
GGGCGUCACGAUUUACGAGGCCGAGCGAUCACCAUCGUCGGUCGCGUUUCAAAACGCCCAUACAUUAACGCUUGGGUAAAGCGGGCUUCGUGCGCUUCCAUCGCUAACGGCCUCUAGAACCCCUGCUACACGU
X1234X   LLLLLLL I1234I             I4321I RRR      X4321XAAAAAA    X1   456X       I1I                      I1I       X654  1X CCCCCC
                  ---
                 /   \
                 \   /
                  G-C
                  C-G
                  C-G
                  G.U
                  G-C
                  A G
5'-(N8)GAUUUACG    CGU(N13)AUA
       ||||||||    |||
       CUAAAUGC GY GCA
```

3' stem 25S.U21 30.12

```
UGGGCGUCACGAUUUACGAGGCCGAGCGAUCACCAUCGUCGGUCGCGUUUCAAAACGCCCAUACAUUAACGCUUGGGUAAAGCGGGCUUCGUGCGCUUCCAUCGCUAACGGCCUCUAGAACCCCUGCUACACGU
X12345X          I1 34567I              I76543 1I       X54321X HHHHHH  XX          LLLLL I1 34I          I43 1I  RRRR             XX AAAAAA
                ---
               /   \
               \   /
                G-C
                C U
                U-A
                U-A
                C C
                  G
5'-(N80)GCGGG    GCCU(N15)ACA
        |||||    ||||
        CGCCC AY CGGA
```

Ot-HAsno108

5' stem 25S.U2251 39.53

```
GACCGUACACUAGAGUCACCGCCCCGCGCGAAGACGCGAUCGCGGGUGCGGCGCUCAGUCCAGCCGACAUUGAACGCACGUGUCUUUGUCUCUCGCGUGUGUUUCGGCGAAUCGCGAGGGAUGGAGGACGCGGCGAAGCGCACGGUAAGCGAGCGGACGCGUUCCGCCACGCCUGGGCGCGAGCCGGACAGUU
X 2 4 6X LLLLLLLL I123 5I                    I5 321I RRRRRX6 4 2 X AAAAAA             X12 456 8X     I 2  567I                                                                    I765  2 I    X8 654 21XCCCCCC
                 ---
                /   \
                \   /
                 C U
                 C-G
                 G-C
                 C-G
                 C-G
                 A C
5'-(N8)CUAGAGUC    GCUCA(N10)ACA
       ||||||||    |||||
       GAUCUCAG UY CGAGU
```

5' stem   25S.U2252 31.58

```
CGGACCGUACACUAGAGUCACCGCCCCGCGCGAAGACGCGAUCGCGGGUGCGGCGCUCAGUCCAGCCGACAUUGAACGCACGUGUCUUUGUCUCUCGCGUGUGUUUCGGCGAAUCGCGAGGGAUGGAGGACGCGGCGAAGCGCACGGUAAGCGAGCGGACGCGUUCCGCCACGCCUGGGCGCGAGCCGGACAGUU
X1X       LLLLLLLI  3456I                     I6543  IrRRRRR      X1XAAAAAA            X12 456 8X     I 2  567I                                                     I765  2 I    X8 654 21XCCCCCC
                ---
               /   \
               \   /
                C-G
                G.U
                C-G
                C C
                A G
                C-G
5'-(N9)ACUAGAGU    CGCUCA(N10)ACA
       ||||||||     |||||
       UGAUCUCA GY UCGAGU
```

3' stem   25S.U2814 26.43

```
GACCGUACACUAGAGUCACCGCCCCGCGCGAAGACGCGAUCGCGGGUGCGGCGCUCAGUCCAGCCGACAUUGAACGCACGUGUCUUUGUCUCUCGCGUGUGUUUCGGCGAAUCGCGAGGGAUGGAGGACGCGGCGAAGCGCACGGUAAGCGAGCGGACGCGUUCCGCCACGCCUGGGCGCGAGCCGGACAGUU
X 2 4 6X          I123 5I                   I5 321I      X6 4 2 X HHHHHH                                X123X       LLLI  345 78I                                 I87 543  IRRRRRR    X321XAAAAAA
             ---
            /   \
            \   /
             G-C
             G-C
             U-A
             A C
             G G
             G-C
5'-(N114)GAG    CUGGGC(N10)ACA
         |||    ||||||
         CUC GY GACCCG
```

Ot-HAsno118

5' stem   18S.U1653 27.39

```
GGGCUCCUCUGCCACGGUUUCCUGAGCUUUGCGCUGACGGUUAACUGUCCAAAGAGAGCCCUACAUAGAUUUUCGCCUUCAUGAUAGGAAGCCGUACUGUCGUCUUCGGGCGUGACUUUGCGGUGCCAACCGGGCGAUUACAUUC
X1234X     LLL I123   67I              I76   321IRRRRRRR  X4321X AAAAAA        X 2X             I123   67I             I76   321I           X2 X    CCCCCC
           ---
          /   \
          \   /
           U U
           U-A
           U-A
           G-C
           G.U
           C
5'-(N10)CCA     GUCCAAA(N10)ACA
        |||     |||||||
        GGU  AY CAGGUUU
```

3' stem 25S.U2704 29.60

```
UCGGGCUCCUCUGCCACGGUUUCCUGAGCUUUGCGCUGACGGUUAACUGUCCAAAGAGAGCCCUACAUAGAUUUUCGCCUUCAUGAUAGGAAGCCGUACUGUCGUCUUCGGGCGUGACUUUGCGGUGCCAACCGGGCGAUUACAUUC
X 23456X       I1234I                        I4321I       X65432 X HHHHHH        XX  LLLLLLLI1234I                        I4321IRRRrRR     XX  AAAAAA
               ---
              /   \
              \   /
               A-U
               U.G
               G-C
               C-G
               C-G
               G.U
5'-(N83)GAUAGGAA     GCCAAC(N10)ACA
        ||||||||     ||| ||
        CUAUCCUU  CY CGGCUG
```

Ot-HAsno119

5' stem   YourSeq.U1074 30.97

```
GUGGGGCCUUCACCCCGGGCGGUGCGGCGUAGACUAUCGCCGGACGCGUCCCAAGCCCCGCGCAACAACCGAAGCGUGCGCCGUGUUGCGCGCCGGGUUCACCGUGCUCGGUCGCGUGUCUUAAAGUUGCACCGACAUUU
X12 4X      LLLLLLI1I                         I1IRRRRR   X4 21X AAAAAA          X 2345 7X       I12 4 6 8I      I8 6 4 21I      X7 5432 XCCCCCC
              ---
             /   \
             \   /
              G A
              U C
              G G
              G-C
              C-G
              G.U
5'-(N11)CCCCGG     CCCAA(N11)ACA
        ||||||     |||||
        GGGGCC  UY GGGUU
```

Ot-HAsno120

3' stem   25S.U32799 43.20

```
CGCGUCGAGGCUCACUCGCGUCGGCGGGACGAUGGACGUUCGUCUCGUCGCGGGGCUUUGACGCGCACAUUUGGUUACGCCAGAUAUAAACCAAUGGCGGGUUAACUGAGUACCCGUCGUUGACCUGUCUCGCUGGUACAUUU
X1234567X        I12 45I                    I54 21I     X7654321X HHHHHH     XX          LLLLI12345678I           I87654321IRRRRRRRR     XXAAAAAA
            ---
           /   \
           \   /
            G.U
            G-C
            U.G
            A-U
            A-U
            C-G
5'-(N86)AAAC     ACCUGUCUC(N7)ACA
        ||||     |||||||||
        UUUG  AY UGGACAGAG
```

Ot-sno125

5' stem   18S.U1146 28.1

```
GCUUCGUUAAGUUGGGCAUUUUACUCCGUCAGUGUGCUCUUGCCCCGUUCCCUUGAAGCUACAUCGAUAGGUCGGCCCGUUGAAUCGAAGCUUCGGGUUCGGUCAUGCAACUGGUCUCUCGCUAUGUUUGCUCGCCGCUCACCGCGCGUGAAUACACAGAGGCCAGACAUCU
X1234XLLLLLLLLI12I                    I21I rRRRRR  X4321X AAAAAA                                                   X1234567X       I1234 67I        I76 4321I     X7654321XCCCCCC
                ---
               /   \
               \   /
                U U
                A-U
                C-G
                G-C
                G-C
                  C
5'-(N5)UUAAGUUG    CGUUCC(N10)ACA
       |||||..|     |.|||
       AAUUCGGC GY CCGAGG
```

5' stem   18S.U343 27.20

```
GUUGGGCAUUUUACUCCGUCAGUGUGCUCUUGCCCCGUUCCCUUGAAGCUACAUCGAUAGGUCGGCCCGUUGAAUCGAAGCUUCGGGUUCGGUCAUGCAACUGGUCUCUCGCUAUGUUUGCUCGCCGCUCACCGCGCGUGAAUACACAGAGGCCAGACAUCU
X 2X         LLLLLL I12I           I21IRRR         X2 X AAAAAA                                                   X1234567X       I1234 67I        I76 4321I     X7654321XCCCCCC
                ---
               /   \
               \   /
                U C
                G-C
                U.G
                G.U
                A-U
                C
5'-(N12)CUCCGU    CCC(N15)AUA
        ||||||    |||
        GAGGCA GY GGG
```

Ot-HAsno126

5' stem   25S.U706 32.47

```
GCUGUUACUUCAGGCAGCCGAGGGCGACAUUCCGUUCGUCUUCGGCGUUAAUCAACCCGUACAGUAACGAGCUCGCAUUCUGAGCGACCGCGGGAAUGGGACUUACUACCCUGUGCCCCAUGCAGUUUGCUCUGGAAACCG
XX       LLLLLLLI12345678I          I87654321IRRRrRRRR    XXAAAAAA             X1 34567X        I123I                 I321I     X76543 1XCCCCCC
                ---
               /   \
               \   /
                G.U
                A-U
                G-C
                C-G
                C-G
                G-C
5'-(N8)UCAGGCA    GUUAAUCA(N7)ACA
       |||||||    ||| |.||
       AGUCCGU AY CAAGUGGU
```

5' stem   U1.U23 27.66

```
GCGGGCUGUUACUUCAGGCAGCCGAGGGCGACAUUCCGUUCGUCUUCGGCGUUAAUCAACCCGUACAGUAACGAGCUCGCAUUCUGAGCGACCGCGGGAAUGGGACUUACUACCCUGUGCCCCAUGCAGUUUGCUCUGGAAACCG
X123X        LLLLL I1 345I            I543 1I  RRRRR       X321XAAAAAA             X1 34567X        I123I                 I321I     X76543 1XCCCCCC
                ---
               /   \
               \   /
                C-G
                C U
                G-C
                A-U
                C U
                  C
5'-(N12)UCAGG    GGCGU(N13)ACA
        |||||    |||||
        AGUCC GY CCGCA
```

3' stem   25S.U839 25.77

```
CGGGCUGUUACUUCAGGCAGCCGAGGGCGACAUUCCGUUCGUCUUCGGCGUUAAUCAACCCGUACAGUAACGAGCUCGCAUUCUGAGCGACCGCGGGAAUGGGACUUACUACCCUGUGCCCCAUGCAGUUUGCUCUGGAAACCGGGCUUAUACUU
X123 5X         I 23 56I             I65 32 I         X5 321X HHHHHH  X123X   LLLLLI1234I                                                       I4321IrRRRRRR  X321XAAAAAA
                ---
               /   \
               \   /
                A-U
                G.U
                C-G
                G-C
                A-U
                G-C
5'-(N78)AUUCU    UGGAAAC(N8)AUA
        ||.||     ||||||
        UAGGA CY CCCUUUG
```

3' stem   U4.U26 26.58

```
CGGGCUGUUACUUCAGGCAGCCGAGGGCGACAUUCCGUUCGUCUUCGGCGUUAAUCAACCCGUACAGUAACGAGCUCGCAUUCUGAGCGACCGCGGGAAUGGGACUUACUACCCUGUGCCCCAUGCAGUUUGCUCUGGAAACCGGGCUUAUACUU
X123 5X         I 23 56I             I65 32 I         X5 321X HHHHHH  X123X  LLLLLLI1234I                                                       I4321IrRRRRRR  X321XAAAAAA
                ---
               /   \
               \   /
                A-U
                G.U
                C-G
                G-C
                A-U
                G-C
5'-(N77)CAUUCU    UGGAAAC(N8)AUA
        ||.|||     ||||||
        GUGAGA GY UCCUUUG
```
